# Rapid range shifts and megafaunal extinctions associated with late Pleistocene climate change

**DOI:** 10.1038/s41467-020-16502-3

**Published:** 2020-06-02

**Authors:** Frederik V. Seersholm, Daniel J. Werndly, Alicia Grealy, Taryn Johnson, Erin M. Keenan Early, Ernest L. Lundelius, Barbara Winsborough, Grayal Earle Farr, Rickard Toomey, Anders J. Hansen, Beth Shapiro, Michael R. Waters, Gregory McDonald, Anna Linderholm, Thomas W. Stafford, Michael Bunce

**Affiliations:** 1 Trace and Environmental DNA (TrEnD) Laboratory, School of Molecular and Life Sciences, Curtin University, Bentley, WA 6102 Australia; 2 Division of Ecology and Evolution, Research School of Biology, ANU College of Science The Australian National University, Canberra, ACT 2600 Australia; 3 Bioarchaeology and Genomics Laboratory, Department of Anthropology, Texas A&M University, College Station, TX 77843 USA; 4 Department of Geosciences, Jackson School of Geological Sciences, The University of Texas at Austin, Austin, TX 78712 USA; 5 Department of Geosciences, Vertebrate Paleontology Laboratory, Jackson School of Geological Sciences, The University of Texas at Austin, Austin, TX 78712 USA; 6 Department of Integrative Biology, The University of Texas, Austin, TX 78712 USA; 7 Winsborough Consulting, Leander, TX 78641 USA; 8 Department of Anthropology, Florida State University, Tallahassee, FL 32310 USA; 9 Mammoth Cave National Park, PO Box 7, Mammoth Cave, KY 42259 USA; 10 Centre for GeoGenetics, Department of Biology, University of Copenhagen, DK-1350 Copenhagen, Denmark; 11 Department of Ecology and Evolutionary Biology, University of California Santa Cruz, Santa Cruz, CA 95064 USA; 12 Howard Hughes Medical Institute, University of California Santa Cruz, Santa Cruz, CA 95064 USA; 13 Center for the Study of the First Americans, Department of Anthropology, Texas A&M University, College Station, TX 77843-4352 USA; 14 Bureau of Land Management, Utah State Office, 440 West 200 South, Salt Lake City, UT 84101-1345 USA; 15 Stafford Research LLC, Lafayette, CO 80026-1845 USA

**Keywords:** Climate-change ecology, Palaeoecology, Next-generation sequencing, Palaeoclimate

## Abstract

Large-scale changes in global climate at the end of the Pleistocene significantly impacted ecosystems across North America. However, the pace and scale of biotic turnover in response to both the Younger Dryas cold period and subsequent Holocene rapid warming have been challenging to assess because of the scarcity of well dated fossil and pollen records that covers this period. Here we present an ancient DNA record from Hall’s Cave, Texas, that documents 100 vertebrate and 45 plant taxa from bulk fossils and sediment. We show that local plant and animal diversity dropped markedly during Younger Dryas cooling, but while plant diversity recovered in the early Holocene, animal diversity did not. Instead, five extant and nine extinct large bodied animals disappeared from the region at the end of the Pleistocene. Our findings suggest that climate change affected the local ecosystem in Texas over the Pleistocene-Holocene boundary, but climate change on its own may not explain the disappearance of the megafauna at the end of the Pleistocene.

## Abstract (web summary)

The impact of late Pleistocene climate change on ecosystems has been hard to assess. Here, the authors sequence ancient DNA from Hall’s Cave, Texas and find that both plant and vertebrate diversity decreased with cooling, and though plant diversity recovered with rewarming, megafauna went extinct.

## Introduction

The cause of the late Quaternary extinctions across the globe has been debated since the beginning of modern science^1^. Although many hypotheses have been suggested, including disease and extraterrestrial impacts, the two main theories implicate human hunting and climate change as the main drivers of these extinctions^2^. In North America, major human immigrations and climate changes occur nearly simultaneously, complicating efforts to disentangle their relative contributions. The situation is particularly challenging, given the rarity of archaeological and palaeontological sites dating to the interval of these extinctions.

Several archaeological sites document that Palaeo-Indian groups targeted at least six of the 36 megafaunal genera^3^ in North America, providing some support for human hunting as a cause of megafaunal extinctions on the continent^4^. However, while people were widespread across the Americas by at least 13,000 cal BP^3,5,6^, their population sizes were small, and it remains unresolved how strongly hunting affected megafauna populations^3,7^. This has led to the widely accepted “one–two punch” hypothesis^8^, whereby the combined effects of climate change and human impacts led to the extinction of the North American megafauna by approximately 13.0–12.5 ka cal BP^9,10^.

Unlike Australia^11^ and New Zealand^12^, where human arrivals and major climate changes are largely decoupled and separated by thousands of years, major climate changes in North America occurred coincidentally with human arrival. By 14,700 cal BP (14.7 ka cal BP)^13^, rising temperatures during the Bølling–Allerød warming event caused the continental ice sheets to retreat, ending the Last Glacial Maximum. Two millennia later, this warming reversed abruptly during the Younger Dryas Cooling Event (12.9–11.7 ka cal BP), when temperatures rapidly dropped in the Northern Hemisphere^14^. Understanding how these dramatic changes influenced biodiversity at a local scale could provide new insights into the causes of the global mass extinction event.

However, current estimates of the amplitude of temperature fluctuations during the late Quaternary rely on data from ice cores in Greenland, which are not readily translated to central North America. For example, the severity of the YD climate change on the Great Plains is debatable, and the event has been described both as “near glacial conditions”^15^ and as a period with mean annual temperatures no more than ~5 °C cooler than present^16^. Furthermore, the effect of seasonality during the Younger Dryas is not accounted for with traditional proxies for mean annual temperature, and the apparent cooling during the YD can represent an increased seasonality with cold winters but relatively warm summers^17^.

To investigate the speed and extent to which an entire ecosystem responded to the climatic fluctuations from the Pleistocene to the Holocene, we sequence ancient DNA from vertebrate fossils and sediment excavated from Hall’s Cave, a limestone cavern located on the Edwards Plateau, Texas (USA)^18^. The Edwards Plateau is a 600–800-m elevation limestone plateau in north–central Texas that consists of hilly grasslands and open woodlands^19^, with current mean annual temperatures ranging from 17 to 20 °C. Our study site, Hall’s Cave, provides an ideal location for this work because it contains a well-dated sedimentary record with chemically and physically well-preserved vertebrate remains deposited from the Last Glacial Maximum through to the present^20^. Hall’s Cave is also one of the few cave sites with finely stratified sediments deposited during the Younger Dryas^21^. To study changes in the Hall’s cave assemblage over time, we use a combination of two ancient DNA approaches: bulk bone metabarcoding (BBM)^22^ and sedimentary ancient DNA (*sed*aDNA)^23^. Our data, in combination with existing palaeoecological studies from the Texas region, provide a detailed chronology of biodiversity turnover against the backdrop of impacts from both human arrivals and climate shifts in central North America.

## Results

### Sample collection and sequencing

**Fig. 1:**
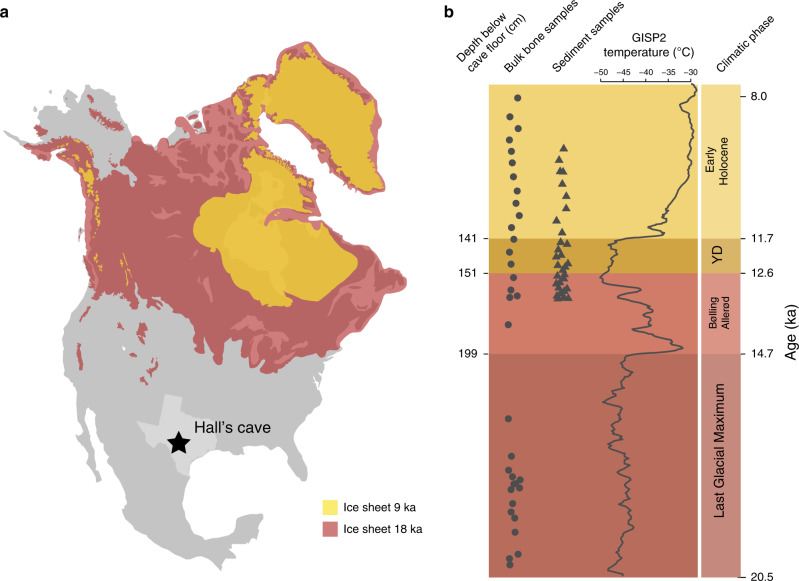
Sampling location and stratigraphy. **a** Location of Hall’s Cave in North America, with the continental ice sheets and mountain glaciers shown at 18,000 and 9000 cal BP^65^. **b** Left panel, sample ages at Hall’s Cave for bulk-bone samples (circles) and sediment samples (triangles) based on calibrated ages (ka cal BP). Middle panel, ambient temperature over Greenland based on δ^18^O values, dated by counting annual accumulation layers (years before Y2k; Greenland Ice Sheet Project 2—GISP2^14^). Right panel, time period sectioning.

We excavated bulk-bone samples from strata dating from the Last Glacial Maximum to the early Holocene at Hall’s Cave (Fig. 1). We used DNA metabarcoding (Methods) to characterise the faunal assemblage across 30 newly excavated bulk-bone samples of approximately 100 small non-diagnostic bones each, as well as from six bulk-bone samples made from large fragmentary fossils excavated by Toomey in 1993^18^ (Fig. 1, Supplementary Table 1). We targeted short mitochondrial barcoding regions of *12S rRNA*^24^ and *16S rRNA*^25^ (Supplementary Table 2), and sequenced a total of 2,313,843 reads from *12S* and 2,315,462 reads from *16S* (Supplementary Tables 3 and 4). We also excavated 32 sediment samples to characterise the floral assemblage across the Pleistocene–Holocene boundary (Fig. 1, Supplementary Table 4), from which we amplified two short chloroplast loci: the P6 loop of *trnL*^26^ and a fragment of *rbcL*^27^(Supplementary Table 2). We generated 2,066,643 reads representing 828,118 and 1,111,635 reads from the *trnL* and *rbcL* genes, respectively (Supplementary Table 5). We explored aDNA preservation throughout the chronological sequence (see Supplementary Note 1, Supplementary Figs. 1 and 2) and found no evidence of systematic changes in DNA preservation over time. Hence, we concluded that our aDNA-derived assemblages reflect temporal shifts in the composition of the community surrounding Hall’s Cave rather than DNA preservation bias, while acknowledging that all fossil deposits can be influenced by taphonomy (see Supplementary Note 1).

Similar to many North American geoarchaeological sites, the profile in Hall’s Cave has a clearly visible “rancholabrean mat” or organic-rich layer associated with the Younger Dryas (Supplementary Fig. 3)^15^. We define the base of this layer (151-cm BD_T_) as the onset of the YD. Based on the current age-depth model (Supplementary Fig. 4), 151-cm BD_T_ dates to 12.6 ka cal BP (12,692–12,396 ka cal BP)—slightly younger than the conventional dates for the onset of the YD at 12.9 ka cal BP. This apparent time lag between the cooling over Greenland and a biological response in North America was also reported for several pollen records in the Great Lakes region^28,29^. However, the difference could also be explained by uncertainties in the age-depth model, or contamination with humic acids from younger overlying layers.

We grouped samples based on four distinct climate intervals for comparative analyses: (1) Last Glacial Maximum (ca. 20–14.7 ka cal BP), a period of cold glacial-era conditions; (2) Bølling–Allerød (14.7–12.6 ka cal BP), a warmer interstadial with oscillating temperatures; (3) Younger Dryas (12.6–11.7 ka cal BP), a short and abrupt cooling in the Northern Hemisphere; (4) Early Holocene (11.7–8.0 ka cal BP), a progressively warming period^30^ (see Fig. 1). We processed sediment samples from the latter three time periods (Bølling–Allerød, Younger Dryas and Early Holocene) and bulk-bone samples from throughout the profile (see Fig. 1).

### Overall biodiversity at Hall’s Cave

**Fig. 2:**
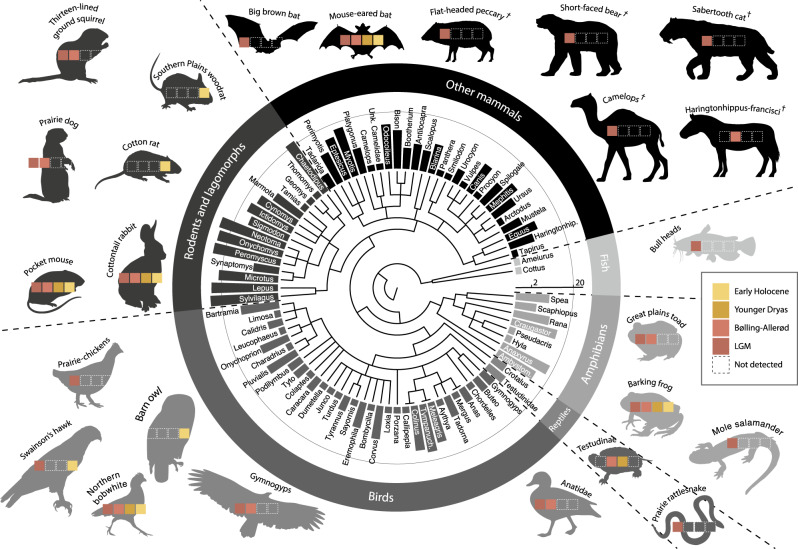
Overall vertebrate diversity derived from bulk-bone metabarcoding (BBM). Dendrogram of genera detected by BBM at Hall’s Cave (Texas), with silhouettes illustrative of some of the detected taxa. Bar heights represent the number of bulk-bone layers (*n* = 36) in which each genus was detected. See Supplementary Tables 6–8 for a complete list of taxa detected. Daggers highlight extinct species.

In agreement with the osteological record^18^, we identified a high level of vertebrate animal diversity at Hall’s Cave using bulk-bone metabarcoding. In total, we detected at least 100 different vertebrate species: 50 mammals, 36 birds, 9 amphibians, 3 reptiles and two fishes (Fig. 2, Supplementary Tables 6–8). Lagomorphs, rodents and bats are ubiquitous throughout the assemblage (Supplementary Table 6). The deer mouse (*Peromyscus* spp.) was the most commonly identified species, followed by cottontail rabbit (*Sylvilagus* spp.), and eastern woodrat (*Neotoma floridana*). Large-bodied herbivores, such as bison (*Bison* spp.) and deer (*Odocoileus* spp.) were also detected frequently, albeit primarily in Pleistocene layers.

Despite sampling only a fraction (2957 bone fragments) of what was screened by Toomey (hundreds of thousands of identifiable subfossils), we report an assemblage that is largely consistent with his results. Using aDNA, we detected 36 of the 56 mammal genera that Toomey reported at Hall’s Cave^18^, plus seven mammal genera not previously identified (see Supplementary Fig. 5). For some genera, such as *Neotoma*, genomic data improved the taxonomic resolution by identifying taxa to species level. In addition, because the main focus of previous morphological analyses was mammal diversity, most records for birds, amphibians, reptiles and fish species reported here are new additions to the faunal assemblage at Hall’s cave.

To compare the DNA assemblage obtained from bulk-bone samples with the sedimentary genetic record, we also analysed 10 sedimentary samples using the vertebrate assays employed for bulk-bone metabarcoding (Supplementary Table 9). In agreement with both the morphological and the bulk-bone record, we found rodents to be abundant. However, we detected DNA from felids in 7 of the 10 sediment samples analysed, whereas we only detected felid DNA (jaguar and saber-toothed cat) in two out of 36 bulk bone samples. Jaguar (*Panthera onca*) is the most abundant felid in the sediment samples (present in five samples), followed by bobcat (*Lynx rufus*) in two samples. This discrepancy could suggest that felids deposited significant amounts of DNA from sources other than bone, such as faeces, urine and the shedding of hair, and may indicate that felids resided in the cave towards the end of the Pleistocene.

Our analysis of plant aDNA in the sediment samples revealed a diverse floral record dominated by hackberry (*Celtis* spp.) and oak (*Quercus* spp.). These two taxa represent the two most abundant ASVs (amplicon sequence variant) detected in each assay (Supplementary Fig. 6), and were present in almost all samples analysed (Supplementary Table 10). However, the high abundance of *Celtis* does not necessarily reflect a dominance of hackberry in the local flora. The abundance could reflect that hackberry trees were growing in the entrance of Hall’s Cave as they do today^18^. The abundance of oak in the record, on the other hand, most likely reflects the vegetation in the area. Similar to hackberry and oak, mulberry (*Morus*) and currant (*Ribes*) occur uniformly throughout the entire sequence, and their abundances do not change substantively over time. Other arboreal taxa, e.g., juniper (*Juniperus*), walnut (*Juglans*) and ash (*Fraxinus*) have disjunct occurrences over time. Our *sed*aDNA plant record generally agrees with previous palynological results from the site^31^ when comparing the most abundant taxa from both approaches. However, the absence of *Pinus* DNA directly contradicts the pollen record, where *Pinus* is the most abundant taxa. As *sed*aDNA is local in origin compared with pollen that can be transported over long distances^32^, this could suggest that *Pinus* was not present in the local area around Hall’s Cave, despite displaying a strong signal from pollen.

### Community composition shifts during the Younger Dryas

**Fig. 3:**
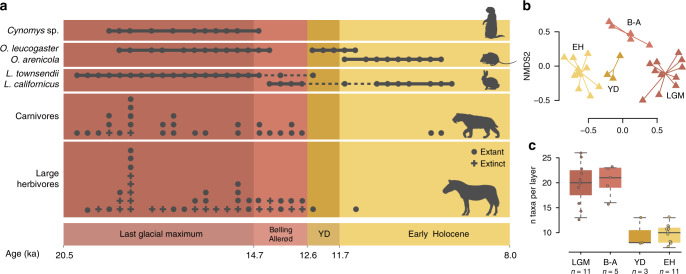
Vertebrate diversity through time tracked via bulk-bone metabarcoding (BBM). **a** The lower two panels represent the number of different extinct and extant species of large herbivores (>30 kg) or carnivores, respectively, that were identified in each layer. The upper two panels indicate population replacement of selected indicator species. **b** Non-metric multidimensional scaling (NMDS) plot based on BBM results from samples excavated for this study (Supplementary Table 1). **c** Number of taxa detected per layer for samples excavated for this study illustrated with boxplots (centre line: median. Box limits: upper and lower quartiles. Whiskers extend to the extremes of the data, no data points were excluded). A total of 30 biologically independent samples from four time periods were compared: LGM (*n* = 11), Bølling–Allerød (*n* = 5), YD (*n* = 3) and Early Holocene (n = 11). Source data are provided as a Source Data file.

Non-metric multidimensional scaling (NMDS) analysis revealed that both the plant and animal assemblages at Hall’s Cave changed significantly across the transition from the Late Pleistocene to the early Holocene. NMDS based on taxa revealed that the vertebrate data formed clusters based on time period (Fig. 3, *P* < 0.001, anova.cca, 999 permutations). This pattern persisted even after taking a taxonomy-independent approach based on ASV diversity, which controls for uneven representation of species in the genomic reference database used (see Methods; Supplementary Fig. 7). For the plants, the Bølling–Allerød time period formed a distinct cluster, while the Younger Dryas and Early Holocene taxa clustered closer together (*P* < 0.001, anova.cca, 999 permutations). However, when taking a taxonomy-independent approach, only the *trnL* assay, which is more variable than *rbcL*, separated the three time periods. The more conserved *rbcL* assay is not able to reliably resolve the different time periods primarily due to the limited taxonomic resolution of the assay (Supplementary Fig. 7).

**Fig. 4:**
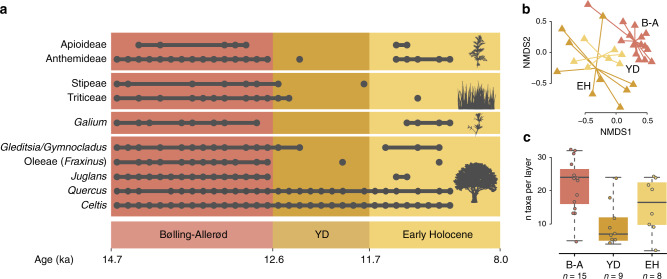
Plant diversity through time. Record is based on *sed*aDNA data of two short chloroplast assays (trnL-gh and rbcL). **a** Detection of select indicator species through time. It includes data from sequences A and B sorted by age (Supplementary Table 4). **b** Non-metric multidimensional scaling (NMDS) plot based on presence/absence data of all taxa detected by *sed*aDNA. **c** Number of taxa detected per layer for different time periods illustrated with boxplots (centre line: median. Box limits: upper and lower quartiles. Whiskers extend to the extremes of the data, no data points were excluded). A total of 32 biologically independent samples from three time periods were compared: Bølling–Allerød (*n* = 15), YD (*n* = 8) and Early Holocene (*n* = 9). Source data are provided as a Source Data file.

The alpha-diversity of plants and animals within each layer displayed different patterns of species loss and recovery over time (Figs. 3c and 4c). For vertebrates, diversity significantly declined (*t* test, *P* = 1.739e−09) from the LGM and Bølling–Allerød time periods (mean = 20.0, SD = 3.8) to the YD and Early Holocene (mean = 9.8, SD = 2.0; Supplementary Fig. 8). This pattern of species loss is also present when characterising different taxonomic subgroups separately. Alpha-diversity loss over the B–A/YD boundary is present in birds, reptiles and frogs, mammalian carnivores and large mammalian herbivores, but is absent in small mammals (Supplementary Fig. 9). Similarly, for plant species, diversity significantly declined (*t* test, *P* = 0.0005) from the Bølling–Allerød (mean = 21.7, SD = 7.9) to the Younger Dryas (mean = 10.1, SD = 6.8). However, plant diversity increased at the end of the Younger Dryas to the Early Holocene (mean = 15.5, SD = 8.2; Supplementary Fig. 10). This post-Younger Dryas increase in diversity is due to a combination of some species returning as temperatures rose and the appearance of new immigrants, including dayflowers (*Commelina*) and red bud (*Cercis*, Fig. 4).

### Biotic shifts in response to temperature fluctuations

Changes in small-mammal diversity detected in our data closely mirror Greenland temperature fluctuations during the Pleistocene–Holocene transition. For example, we detect northern species such as bog lemming (*Synaptomys cooperi*) and least weasel (*Mustela frenata*) in LGM layers. Furthermore, the cold-adapted northern grasshopper mouse (*Onychomys leucogaster*), is present in the LGM, disappears temporarily during the Bølling–Allerød warming, returns during the cold Younger Dryas and then disappears permanently as the early Holocene warming begins (Fig. 3). Similarly, the warm-adapted black-tailed jackrabbit (*Lepus californicus*) is present during the warm Bølling–Allerød and early Holocene periods, but is absent during the Younger Dryas. These range shifts illustrate the patterns of oscillating geographical distributions that many species exhibited during the glacial and interglacial cycles of the Quaternary^33^.

Based on the current realised climatic niches of species present in each time period, we are able to infer past temperatures around Hall’s Cave. In LGM strata, the presence of cold-adapted species, such as northern grasshopper mouse (*Onychomys leucogaster*) and white-tailed jackrabbit (*Lepus towsendii*), suggests a significantly colder climate than today’s 17–20 °C mean annual temperature on the Edwards Plateau. Based on the current range of the white-tailed jackrabbit, we estimate an upper temperature limit of 10.6 °C for this species (Supplementary Fig. 11). This suggests that the mean annual temperature in central Texas was below this level during the LGM. The disappearance of the big brown bat (*Eptesicus fuscus*) at the beginning of the Bølling–Allerød, on the other hand, suggests a substantial warming in central Texas because big brown bat colonies require an ambient cave temperature of <5 °C to hibernate^34^. In addition, the warm-adapted Mearns’s grasshopper mouse (*Onychomys Arenicola*; Supplementary Fig. 12) and cotton rat (*Sigmodon* spp.; Supplementary Fig. 13) move into the region for the first time at the onset of Holocene warming. Given the present-day temperature niche limits of cotton rat (>10.9 °C) and Mearns’s grasshopper mouse (>11.8 °C), this indicates that the mean annual temperature in Texas had risen to above 11 °C in the early Holocene^35^.

### Decreasing rainfall and the denudation of Central Texas

The disappearance of wetland and burrowing taxa after the LGM suggests that the climate of the Edwards Plateau changed from wet conditions during the LGM to drier conditions in the Holocene. One of the strongest drivers separating LGM and Bølling–Allerød strata from YD and early Holocene layers in our ordination analyses (Fig. 3b) is the abundance of burrowing mammals during the LGM and B–A. These fossorial taxa comprise pocket gophers (*Geomys texensis* and *Thomomys bottae*) and prairie dog (*Cynomys* sp.), which are found exclusively in LGM layers, and additional species that persist into the Bølling–Allerød time period, including thirteen-lined ground squirrel (*Ictidomys tridecemlineatus*) and marmot (*Marmota* spp.). Of particular interest for palaeoenvironmental reconstruction is the prairie dog, which disappears at the onset of the Bølling–Allerød (Fig. 3a). With a present-day mean burrowing depth of 140 cm^36^, the presence of prairie dog indicates that soil thicknesses in central Texas was dramatically deeper than today (probably >100 cm) up until ca. 14,700 years cal BP, but decreased soon after. In addition, the disappearance of marmot, which requires soil depths of 40–140 cm^36^, approximately a thousand years later, indicates further decreases in soil thickness. Lastly, by the onset of the Younger Dryas, the eastern mole (*Scalopus aquaticus*) disappeared from the region, indicating that soil depth was <25 cm^18^ by this time. These soil-depth decreases are consistent with previous estimates based on strontium isotope studies at Hall’s Cave^20^. By the end of the Younger Dryas in Central Texas, soils were very thin and similar to today’s few-centimetre thick pedogenic horizons. This trend of decreasing soil cover and regional drying is also reflected by the aDNA record of birds and amphibians, which details the disappearance of wetland-adapted species such as ducks, swans and geese (*Anatidae* sp.) and mole salamander (*Ambystoma* sp.) at the end of the LGM (Supplementary Tables 7 and 8).

### Ancient plant DNA compared with other palaeoecological records

Our *sed*aDNA plant record generally agrees with previous palynological results from Hall’s Cave^31^ when comparing the most abundant taxa from both approaches. All of the taxa detected by the most common ASVs in this study (Supplementary Fig. 6) were also detected by pollen, although at different taxonomic levels. *Stenaria*, for example, which was commonly detected in our study, was identified at family level (Rubiaceae) in the pollen data. Similarly, of the ten most abundant taxa identified by pollen, only *Pinus* and Chenopodiaceae were not identified in the DNA data (Supplementary Table 10). However, the abundances of taxa identified vary widely between the two approaches. Hackberry (*Celtis*), for example, is identified as the most common read in all sedaDNA samples, but is rare in the pollen record. In addition, the absence of *Pinus* DNA contradicts the pollen record, where *Pinus* is the most abundant taxa. This discrepancy is likely a result of the different nature of the proxies examined. *Pinus* pollen is known for being overrepresented in fossil pollen records due to its high pollen productivity and dispersability^37^, while *sed*aDNA is local in origin^32^. Furthermore, previous results have demonstrated that pollen is essentially devoid of chloroplast DNA^23^, explaining the absence of *Pinus* DNA, despite the presence of its pollen. Hence, although pollen records from Hall’s Cave and other parts of central Texas suggest that the region was covered in conifer forests during full-glacial times, the lack of conifer aDNA in our samples suggests that coniferous taxa were not present in the local area, but were aeolian-derived from distant sources. Nevertheless, as discussed below, the overall patterns of vegetational change from the Pleistocene to the Holocene are consistent between the two approaches.

### Vegetational change on the Edwards Plateau

Turnover in fauna detected in our bulk-bone record supports a change in vegetation around Hall’s Cave from a prairie grassland to an open woodland by the end of the LGM. One of the most notable species changes is in prairie chickens, which are ubiquitous during LGM (detected in 13 of 17 LGM layers), but disappear during the Bølling–Allerød warming (Supplementary Table 7). Present-day prairie chickens are known to actively avoid trees^38^; therefore, their disappearance suggests an increase in woody plant cover. This aligns with the appearance of two open woodland-adapted species, the northern bobwhite (*Colinus virginianus*) and wild turkey (*Meleagris gallopavo*), both of which appear in the Bølling–Allerød strata as prairie chickens disappear. Other grassland-adapted species such as horned lark (*Eremophila alpestris*), upland sandpiper (*Bartramia longicauda*) and plover (*Pluvialis* sp.) are also abundant in LGM layers, but disappear from the record during the Bølling–Allerød warming. In the amphibian community, this vegetational change is supported by an increase in the relative abundance of barking frog (*Craugastor augustii*) from LGM layers (2/17 layers) to the Bølling–Allerød period (4/5 layers). The barking frog depends on trees, as they typically inhabit leaf litter, where they feed on insects and use moisture trapped in the humus^39^.

The change in vegetation inferred from the bulk-bone aDNA record is consistent with that reconstructed using *sed*aDNA, which indicates that mesic open woodland during the Bølling–Allerød period changed progressively into dry vegetation with a decrease in tree cover during the Younger Dryas. In the Bølling–Allerød, ash (*Fraxinus*) and walnut (*Juglans*) are more common, which, together with the higher abundance of grasses (e.g., Triticeae and Stipeae) and sumac shrubs (*Toxicodendron*), indicate a live oak woodland. The Younger Dryas is characterised by the absence of many key warm-climate species such as sagebrush (*Anthemideae*), bedstraw (*Galium*) and walnut. These three species disappear during the Younger Dryas, but reappear in the Early Holocene as temperatures increased (Fig. 4a). Loss of diversity during the Younger Dryas is also reflected in the alpha-diversity for both plants and animals, which decreases during the Younger Dryas (Figs. 3c and 4c). The Holocene is characterised by an increase in abundance of juniper (*Juniperus* spp.) and diamond flowers (*Stenaria* spp.), which, in combination with the absence of many grasses (e.g., Stipeae, *Carex* and Hordeinae) suggests a slight change in habitat type to a drier live oak–juniper woodland, with an increase in tree cover.

Despite differences in proxies analysed, our ancient DNA results generally agree with the trends observed in other palaeovegetational records from Hall’s Cave (e.g., faunal remains^18^, pollen^31^, phytoliths^40^ and strontium isotopes^20^). The pollen record from Hall’s Cave indicate that the vegetation during full-glacial conditions was characterised by scattered trees with herbaceous vegetation dominated by C_3_ grasses. The pattern of increased woody plant cover during the Bølling–Allerød discussed above is also reflected in the pollen record with a peak in arboreal pollen at 14 ka cal BP followed by a sharp drop at the beginning of the Younger Dryas^31^. In agreement with these results, Joines (2011)^40^ found evidence of open woodlands or savannahs during the LGM that transitioned into forests during the Bølling–Allerød. Lastly, although not precisely dated, Boriack bog and Gause Bog^41^ in central Texas show a similar trend of decreasing arboreal pollen during the end of the Pleistocene. As noted by Cordova and Johnson, these patterns of a landscape transitioning from an open grassland to a vegetation with an increase in broadleaf trees, suggest an increase in effective moisture during the Bølling–Allerød^42^.

In the pollen data, the YD is characterised by an increase in sagebrush (*Artemisia*) and a decrease in arboreal pollen. In our data, the decrease in arboreal taxa is reflected by the disappearance of Fraxinus and Juglans during this period. However, the increased abundance of sagebrush contradicts the DNA record, where sagebrush disappears in the YD. Nevertheless, the general trend of increased denudation on the Edwards plateau during this period is evident using both proxies. For example, Cordova and Johnson^31^ suggested that the disappearance of *Juglans* sp. that grows in deep soil, could reflect increased erosion in the area at the onset of the YD, in agreement with our interpretation of soil cover thickness.

For large mammals (>30 kg), the transition of the landscape into woodland and the loss of grasslands is accompanied by the disappearance of both extant taxa such as horse (*Equus lambei/scotti/caballus*) and pronghorn (*Antilocapra americana*), and extinct taxa, such as camel (*Camelops* sp.) and flat-headed peccary (*Platygonus compressus*). The disappearance of large herbivores is accompanied by the loss of their predators, e.g., saber-toothed cat (*Smilodon* spp.) and short-faced bear (*Arctodus simus*). These patterns suggest that central Texas experienced a trophic collapse towards the end of the Pleistocene that altered the entire ecosystem. Similar collapses have been documented in modern ecosystems^43,44^. In central Texas, the disappearance of grassland by the end of full-glacial conditions could be explained by the disappearance of the large grazers that maintained this type of vegetation^44,45^.

Further supporting an ecosystem collapse by the end of the Younger Dryas is the detection of non-analogous faunas in Pleistocene strata^45–47^. This suggests that the flora and fauna in the Pleistocene were not just shifted ecosystems compared with the present day, but instead different ecosystems containing communities of species that are not found together today. This is exemplified in our data by detection of pairs of species that do not co-occur in any present fauna of North America, e.g., desert woodrat (*Neotoma lepida*) and shrew (*Blarina* sp.), white-toothed woodrat (*Neotoma leucodon*) and eastern woodrat (*Neotoma floridana*) and white-tailed jackrabbit (*Lepus townsendii*) with barking frog (*Craugastor augustii*).

## Discussion

By comparing bulk-bone metabarcoding with *sed*aDNA excavated from Hall’s Cave, we find multiple lines of evidence supporting dramatic ecological change in central Texas between the LGM and Holocene. Our data indicate that, during the LGM, the Edwards Plateau was a mesic grassland with few to no trees and with thick soils that sustained a diverse population of burrowing mammals (Supplementary Fig. 14). This habitat was maintained by large grazers including bison, helmeted muskox, horses and camels, and the climate was significantly colder than today, with a mean annual temperature below 10.6 °C. With increasing temperatures and changing fauna during the Bølling–Allerød, the grassland transformed into open woodland dominated by live oak, ash and an understory of walnut and sumac shrubs. The loss of grassland habitat in the area at this time coincides with the disappearance of many of the largest grazers from the region, including camel, helmeted muskox and caballine horses. At ~12.6 ka cal BP, the abrupt change in climate to dry and cool conditions during the Younger Dryas coincides with a decline in both plant and animal diversity. Many animal species, such as burrowing mammals, wetland taxa and large mammals disappeared permanently from the area during this time, and the last of the now extinct species to disappear from the record (*Haringtonhippus francisci*) was detected at 153 cm (12.7 ka cal BP), at the onset of the YD. As temperatures rose at the beginning of the Holocene (mean annual temperature > 10.9 °C), plant diversity recovered, and the vegetation transitioned to a live oak–juniper woodland with increased tree cover. The faunal diversity, decimated by megafaunal extinctions, did not recover. Although some previously undetected warm-adapted species appeared in the area in the Holocene, including racoon (*Procyon lotor*), barn owl (*Tyto alba*) and Mearns’s grasshopper mouse, the rich species diversity of mammals was lost.

The significant differences in community responses between terrestrial plants and vertebrate animals during the Younger Dryas have implications for our understanding of megafaunal extinctions in North America. While 35 genera of large mammals went extinct during the late Quaternary^2^, there is only one documented example of extinction in plants. Similarly, our data show that plant diversity recovered after the Younger Dryas, while the diversity in large mammals did not. The fact that plant diversity recovered after the Younger Dryas, but large vertebrates did not, suggests that factors other than climate, including the appearance of humans in the region, may have contributed to the permanent local loss of large mammal diversity. This hypothesis is supported by data from rodents, which like plants, were affected by climate, but not directly by human predation. We show that populations of rats and mice responded rapidly to the changing climate. This is best exemplified by the northern grasshopper mouse population, which disappears, reappears and then disappears again in concordance with its preferred climate niche. This suggests that rodent populations, similarly to plants, adapt to climate changes by migrating with the fluctuating temperature and rainfall regimes. This pattern is not mirrored in the large mammals, which were exposed to the combined effects of climate change and human hunting. Hence, these data suggest that human hunting of large mammals, likely together with climate change at the end of the Pleistocene, led to the extinction of megafauna in North America.

The high degree of biomolecular preservation at Hall’s Cave is rare in North America and contrasts with sites as the Rancho La Brea tar pits, which have failed to yield aDNA despite numerous attempts. Moreover, successful recovery of ancient DNA from sediments in North America has been limited mainly to sites north of the maximum ice sheet extent (Supplementary Fig. 15). The resolution of the Hall’s Cave aDNA data provides the impetus for more expansive follow-up studies using single-bone (mitogenomes or whole genomes) and bulk-bone aDNA, alongside light stable isotopes, ^14^C dating, archaeology, pollen, diatoms and microstratigraphy. Such multidisciplinary approaches are increasingly employed to provide suites of complementary proxies that better quantify our reconstructions of ancient climates, past biodiversity, extinctions and biotic shifts over centuries to millennia.

## Methods

### Study site

Hall’s Cave (30°08′06.3″ N, 99°32′16.4 W) is located in the centre of the Edwards Plateau, a limestone plain in central Texas. Today, the Edwards Plateau consists of hilly grasslands and open woodlands^19^, with a climate classified as arid to semiarid, and mean annual temperatures ranging from 17 to 20 °C (Supplementary Fig. 11). The woody component of the vegetation in the area is dominated by species such as Texas oak (*Quercus texana*), live oak (*Quercus virginiana*), ashe juniper (*Juniperus ashei*) and neatleaf hackberry (*Celtis reticulata*), with a grassland component dominated by C_4_ grasses^18^.

### Dating

An age-depth model was built using Clam (2.3.2)^48^ in R with 23 previously published radiocarbon dates from Hall’s Cave pit 1d/E^20^ (Supplementary Table 11). Based on an initial iteration of the age-depth model, two samples were marked as outliers and removed (TMM 41229-12179 and TMM 41229-12073). The age-depth model was built using a smooth spline with default smoothing (0.3). This model (Supplementary Fig. 4) was used to estimate ages of all samples analysed in this study, except from sample 1C_240_245, which was excavated from pit 1c and not composite pit 1d/E. However, as this sample was excavated from 240 to 245 cm below datum (cm BD_T_), approximately 100 cm below the onset of the Younger Dryas, we are confident that this sample is older than 14.7 ka cal BP, and hence it has been assigned to “Last Glacial Maximum”. All ages reported in this paper are in calibrated years before present (1950 AD); “ka cal BP” represents 10^3^ calendar years before present (1950 AD).

### Sampling

Sediments and bulk-bone samples were excavated from a 4-m-thick section of the eastern face of composite pit 1d/e (Supplementary Fig. 16) in Hall’s Cave in August and September 2016. Sample depths were recorded as cm below the zero datum established by Toomey (cm BD_T_). To better enable future researchers to correlate new and old data collected at the cave, we have included absolute elevation for each sample (Supplementary Tables 1 and 4) from UTM Benchmark data established by Urban Civil, LLC, in August 2016. Bulk-bone samples were collected by excavating sediment in approximately 3-cm levels, which were subsequently dry-sieved through 3- and 1.5-mm sieves to obtain bulk-bone material. In total, 110 levels were excavated for bulk-bone material, yielding from 20 to over 300 bone fragments each. After excavation and sieving, levels yielding fewer than 100 bones were merged, to ensure that all samples could be subsampled to 100 bone fragments (Supplementary Table 1). Sediment samples were collected after bulk-bone sampling by inserting irradiated 50-mL Falcon tubes into the newly exposed excavation face. Samples were collected from two sequences: A and B, in 3- and 1-cm intervals, respectively, both covering the transition between the Bølling–Allerød warming and the Younger Dryas intervals (Supplementary Table 4). All the excavation work, and subsequent handling of the samples, was carried out following ancient DNA guidelines^49^: gloves, hair net, face mask and plastic arm sleeves were worn by excavators, and excavation tools were cleaned with bleach between each excavation unit.

### Extractions

Amplification and sequencing were carried out following the workflow described in Murray et al.^22^ and Seersholm et al.^50^. Briefly, upon import to Australia, samples were transferred to the TRACE (Trace Research Advanced Clean Environment) *a*DNA facility at Curtin University, where all molecular work on the samples was conducted. First, bulk-bone samples were washed in Invitrogen ultrapure distilled water to remove surface sediment from the samples. After drying the samples at room temperature overnight, each bone was weighed and subsampled to less than 100 mg to ensure that all bone fragments were represented by approximately the same mass of bone material. Next, samples were each split into two replicates of 50 bone fragments that were ground to a fine bone powder on a Retsch PM200 planetary ball mill. After grinding, bone powder was extracted using a modified version of the extraction protocol described by Dabney et al.^51^, including extraction blanks for each batch of sample preparation (Supplementary Table 9, Supplementary Fig. 17 and Supplementary Note 2). Briefly, 100–110 mg of bone powder was digested overnight at 55 °C in 1 mL of 0.25 mg/mL Proteinase K in 0.5 M EDTA. Next, samples were centrifuged to pellet undigested debris, and the supernatant was concentrated to 50 µL in a MWCO 30-kDa Vivaspin 500 column (Sigma-Aldrich). Lastly, the concentrate was purified using MinElute silica spin columns (Qiagen) as per the manufacturer’s instructions, except for the use of a modified binding buffer consisting of 40% isopropanol, 0.05% Tween 20, 90 mM NaAc and 5 M guanidine hydrochloride^51^.

Sediment samples were extracted using an approach similar to the bulk-bone extraction protocol described above, but with a few modifications to optimise DNA yield for sediment. First, 2 × 500 mg of sediment for each sample was incubated overnight at 55 °C in a digestion buffer of 0.47 M EDTA, 20 mM TRIS-HCL, 1% Triton X-100 and 1 mg/mL Proteinkinase K. Next, samples were centrifuged, and the supernatant was concentrated to 50 µL in a MWCO 30-kDa Vivaspin 500 column (Sigma-Aldrich). Lastly, subsamples were combined, and the concentrate was cleaned using Qiagen MinElute columns as described above.

All unique sample materials described above (e.g., bulk-bone power, sediment samples and DNA extracts) are available upon request.

### Amplification and sequencing

Vertebrate mitochondrial DNA was amplified from all bulk-bone extracts (Supplementary Table 3) and some sediment extracts (Supplementary Table 9) using the Mam16S and 12SV5 assays (Supplementary Table 2); plant chloroplast DNA was amplified from all sediment extracts using rbcL and trnL-gh assays. All amplifications were carried out in duplicate using 2 µL of DNA extract in a 25-µl reaction containing final concentrations of 2 mM MgCl_2_, 1× Gold PCR buffer, 0.25 mM dNTPs, 0.4 mg/ml bovine serum albumin, 0.6 µL of 0.12× SYBR green in DMSO, 1 U of AmpliTaq Gold DNA polymerase and 0.4 µM forward and reverse primers. Each reaction was amplified with the primers described in Supplementary Table 2, fused to Illumina sequencing adaptors and tagged with a unique combination of 6–8-bp indexes on each primer. PCR cycling conditions consisted of an initial denaturation step of 10 min at 95 °C, followed by 50 cycles of 30 s at 95 °C, 30 s at the annealing temperature (Supplementary Table 2) and 72 °C for 45 s, followed by a final extension step of 72 °C for 10 min. After amplification, duplicates were combined, and samples were pooled in equimolar concentrations and size-selected to 160–450 bp on a Pippin prep (Sage Sciences). Lastly, libraries were cleaned using the Qiagen PCR purification kit (Qiagen) following the manufacturer’s instructions and sequenced with custom- sequencing primers on the Illumina MiSeq platform in single-end mode.

### Sequence analysis

Sequence demultiplexing, filtering and denoising were carried out using a custom-made pipeline based on OBItools^52^ (http://www.grenoble.prabi.fr/trac/OBITools), first described in Seersholm et al.^50^. For the vertebrate assays, demultiplexed and dereplicated fasta files were filtered with the obigrep command, only retaining sequences over 80 bp, and represented by more than 10 reads per sample. Each file was denoised with obiclean using a ratio of 0.2 and an error distance of 2. Denoised sequences were further cleaned with sumaclust using a ratio of 0.5 and a similarity of 95%, followed by a second step with a ratio of 0.01 and a similarity of 93%. Chimeras were filtered out using uchime_denovo from vsearch^53^. For taxonomic assignments, filtered unique reads (ASVs) were queried against the NCBI nt database^54^ (ftp://ftp.ncbi.nlm.nih.gov/blast/db/nt*gz) downloaded on the 25th of August 2018, using megablast^55^. Blast files were parsed using the getLCA blast scripts (https://github.com/frederikseersholm/blast_getLCA) described by Seersholm et al.^56,50^, to automatically assign each read to the lowest common ancestor of the best hit(s) to the database. Automatically assigned taxonomic nodes were investigated and compared with the literature to account for relevant species not present in the database (Supplementary Datas 1 and 2).

For the two plant assays, filtering and taxonomic assignments were carried out as described above, with a few modifications to account for the different nature of assays targeting the chloroplast genome of plants. Using the obigrep command, sequences below 20 bp and represented by fewer than 5 reads per sample were filtered out. Furthermore, we focused on ASVs common across multiple samples by only retaining ASVs detected in three or more samples after filtering. Sequences were queried against the reference database using blastn^55^, and to account for the lower coverage of plant species in Genbank compared with vertebrates, and the high similarity between many plant taxa, we limited the database search to genera present in the contiguous 48 states today (https://plants.sc.egov.usda.gov/adv_search.html). Lastly, we set the lowest possible taxonomic assignment level to genus.

### Statistical analyses

Alpha-diversity analyses were carried out on the datasets of identified taxa (Supplementary Data 3 and 4), excluding contaminants and redundant taxa (e.g., *Onychomys* sp. was excluded as *Onychomys arenicola*, and *Onychomys* *leucogaster* was detected). NMDS analyses were carried out in R using the Vegan package (https://cran.r-project.org/web/packages/vegan/index.html), based on the same datasets as the alpha-diversity estimates. One sample (HCS3) was excluded from the NMDS plot for plants as this sample only contained a single species (*Celtis*). Ordination analyses using a taxonomy-independent approach (Supplementary Fig. 7) were carried out as described above using the ASV tables as input.

### Habitat modelling

Climatic-realised niche limits were based on geographic ranges for relevant species from the The IUCN Red List of Threatened Species (https://www.iucnredlist.org) and precipitation and temperature data at a resolution of 10-min longitude and latitude from WorldClim version 2 (https://biogeo.ucdavis.edu/data/worldclim/v2.1/base/wc2.1_10m_bio.zip)^57^. Temperature and precipitation data were extracted for the geographic ranges of the species *L. californicus*^58^, *Lepus townendii*^59^, *O. arenicola*^60^, *O. leucogaster*^61^, *Sigmodon fulviventer*^62^, *Sigmodon hispidus*^63^ and *Sigmodon ochrognathus*^64^ using the *raster* package in R. Next, mean annual temperature and precipitation were calculated by averaging the monthly values for each data point. For Supplementary Figs. 11–13, 2D kernel density estimates of temperature/habitat were plotted for each species using the geom_density_2d command (MASS) in ggplots2. Temperature niche limits reported in the paper, are the upper and lower limits of temperature data encompassing 95% of the data.

### Reporting summary

Further information on research design is available in the Nature Research Reporting Summary linked to this article.

## Supplementary information

Supplementary Information

Peer Review File

Reporting Summary

Description of Additional Supplementary Files

Supplementary Data 1

Supplementary Data 2

Supplementary Data 3

Supplementary Data 4

## Data Availability

Fastq files for all DNA sequencing data reported in this paper were deposited in the European Nucleotide Archive under study accession number “PRJEB37627 [http://www.ebi.ac.uk/ena/data/view/PRJEB37627]”. The source data underlying Figs. 3c and 4c, and Supplementary Figs. 6, 9 and 17 are provided as a Source Data file. All databases used in this study are publicly available online: “NCBI nt database” [ftp://ftp.ncbi.nlm.nih.gov/blast/db/nt*gz], “UCSD Plants database” [https://plants.sc.egov.usda.gov/adv_search.html], “The IUCN Red List of Threatened Species” [https://www.iucnredlist.org] and “WorldClim version 2” [https://biogeo.ucdavis.edu/data/worldclim/v2.1/base/wc2.1_10m_bio.zip].

## Source data

Source Data

## Code availability

The “getLCA” code used for taxonomic assignments in this study is available at https://github.com/frederikseersholm/blast_getLCA.
