# Supplementary material for: Rapid range shifts and megafaunal extinctions associated with late Pleistocene climate change
Source: Nat Commun. 2020 Jun 2;11:2770. doi: 10.1038/s41467-020-16502-3 (PMC7265304; doi:10.1038/s41467-020-16502-3)
Supplement: Supplementary file 1 — Supplementary Information [file 41467_2020_16502_MOESM1_ESM.pdf]

Supplementary Information for:

Rapid range shifts and megafaunal extinctions associated with late Pleistocene climate change

Seersholm et al. 2020

Supplementary Figures

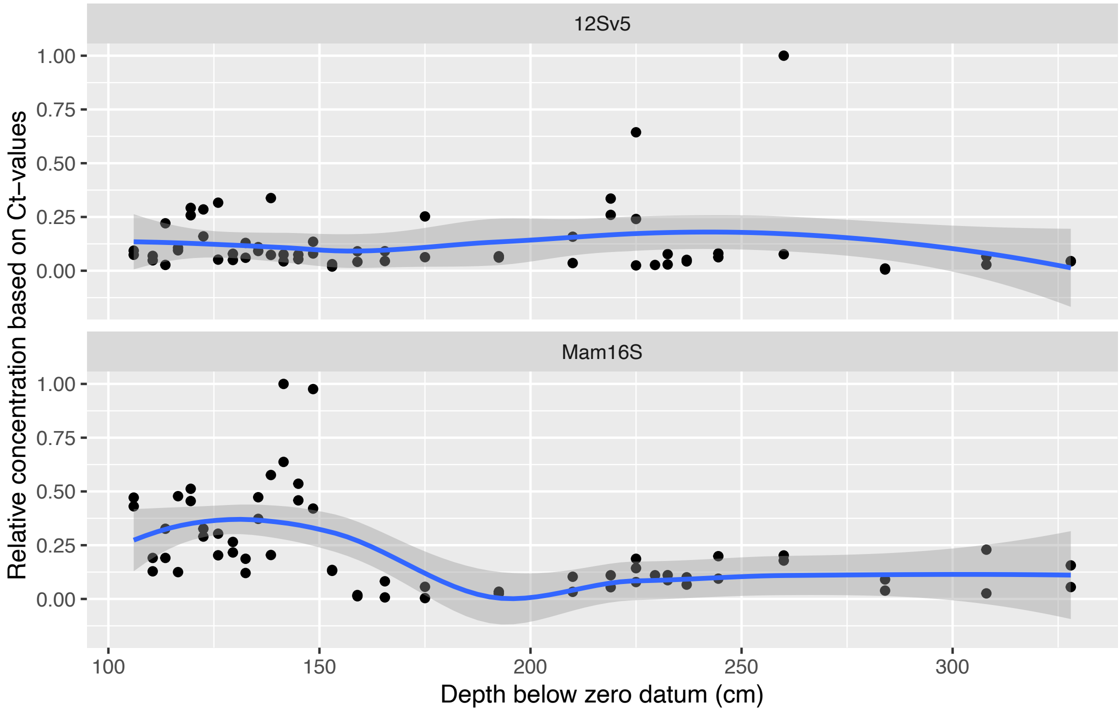

Supplementary Figure 1. DNA concentration in Bulk Bone samples. Concentrations were estimated from Ct-values using the Mam16S and 12Sv5 assay.

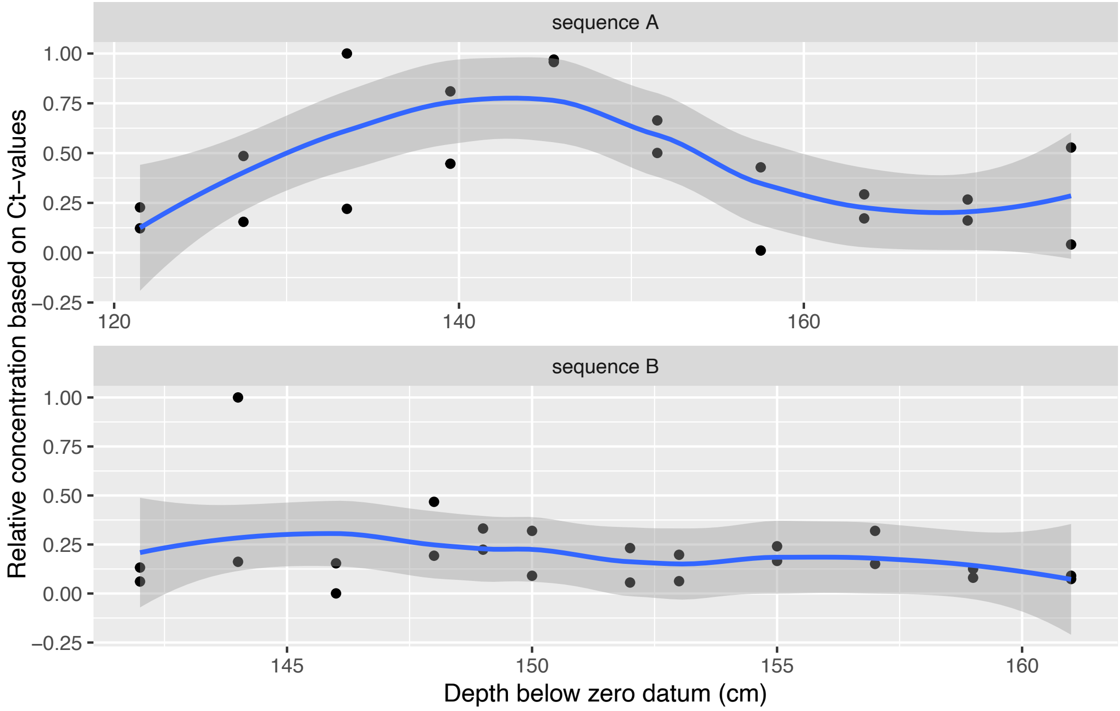

Supplementary Figure 2. DNA concentration in sediment samples. Concentrations were estimated from Ct-values using the trnL assay, for the two excavated sequences, sequence A and sequence B.

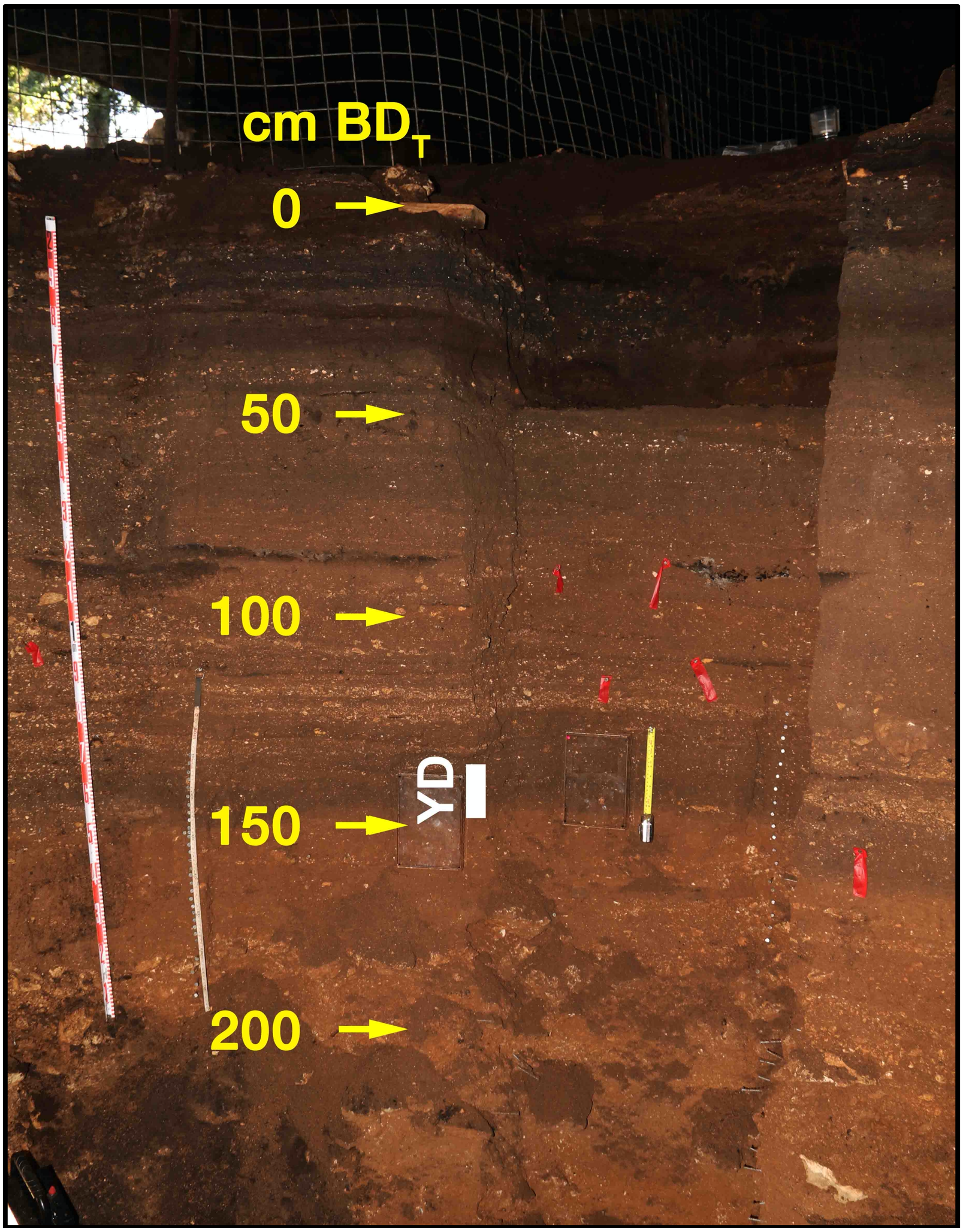

Supplementary Figure 3. 2016 Hall's Cave Excavation for aDNA. View looking East. Depths are relative to the Toomey Datum (cm BDT). The modern cave floor at the excavation square is 0 cm. Younger Dryas (YD) sediments dating ca. 10,900 to 10,200 RC yr BP are designated by the white bar. Photo by T.W. Stafford, Jr., September 8, 2016

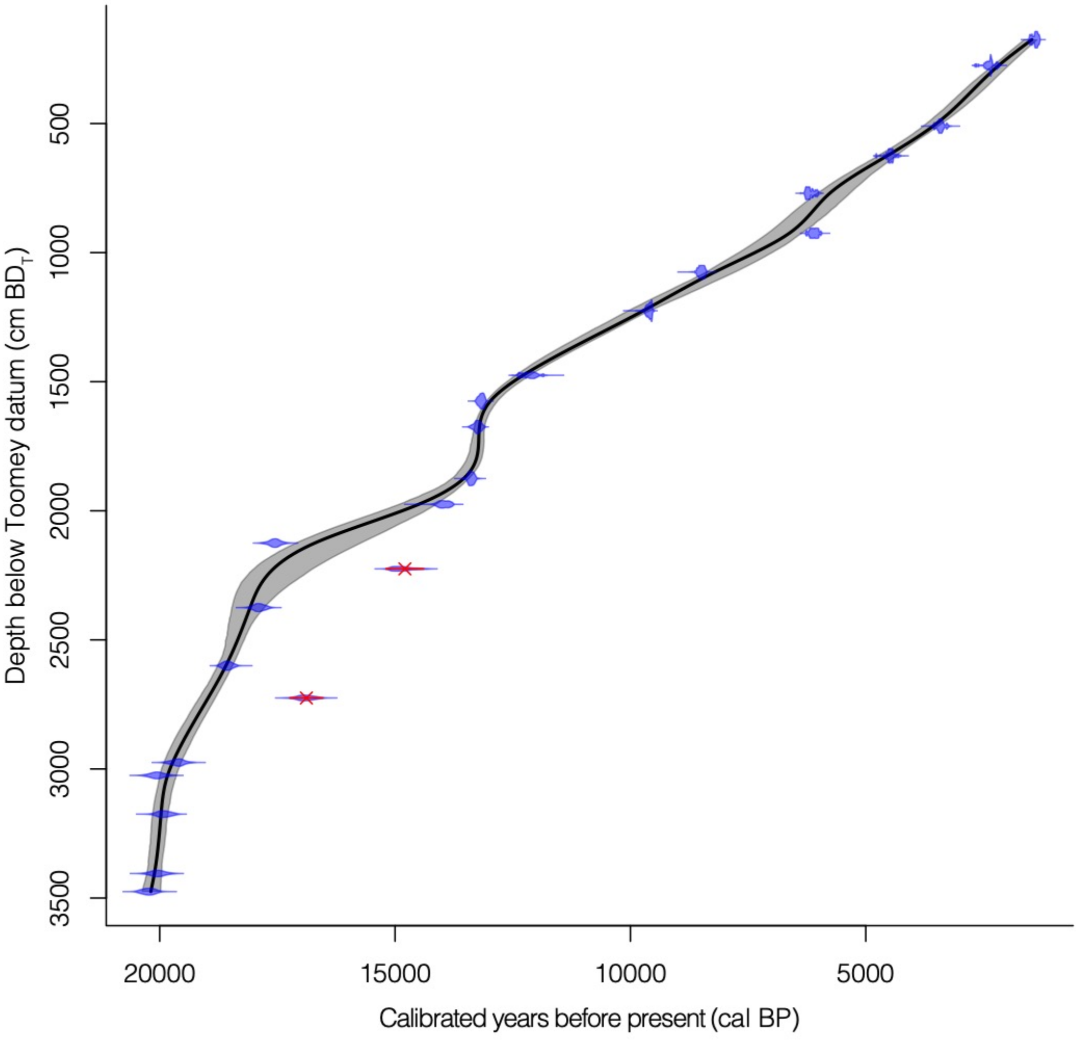

Supplementary Figure 4. Age-depth model.

Total number of mammal genera: **63**

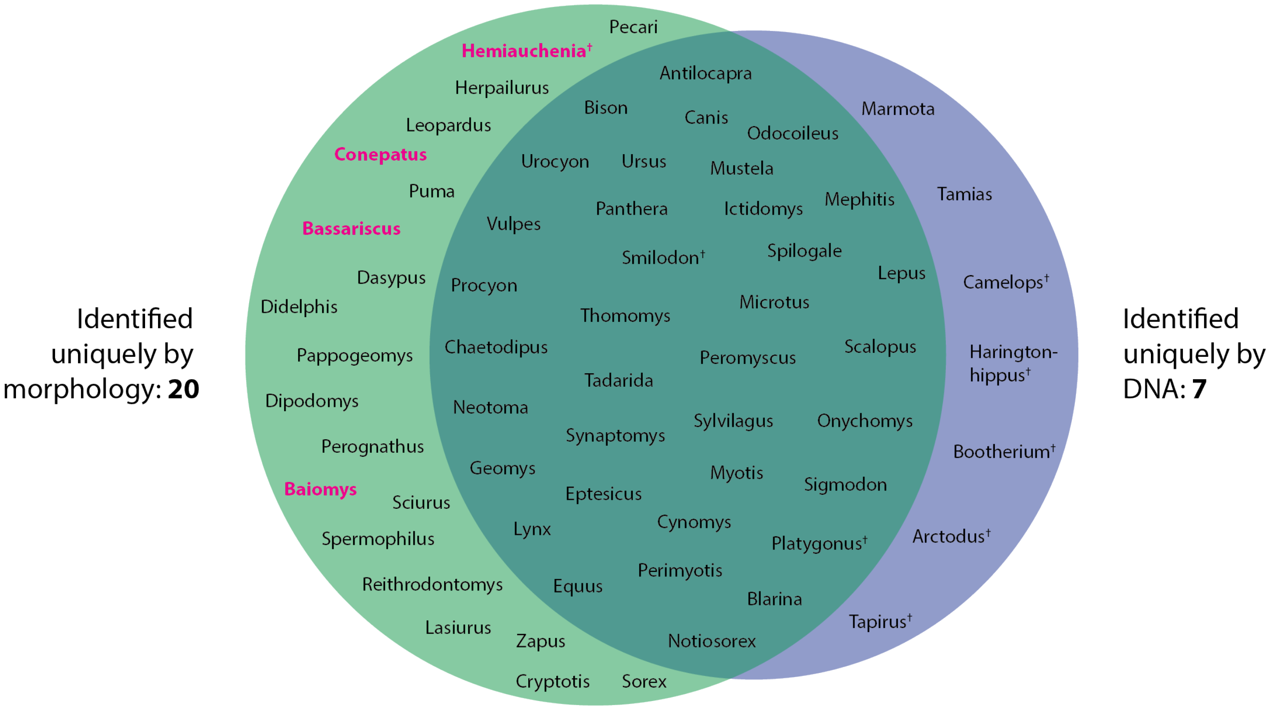

Identified by both morphology and DNA: **36**

Supplementary Figure 5. Comparison of mammal genera found by morphology and DNA. Pink colour indicates genera that cannot be detected by our assay because no relevant reference sequence is available. Crosses (†) indicate extinct genera.

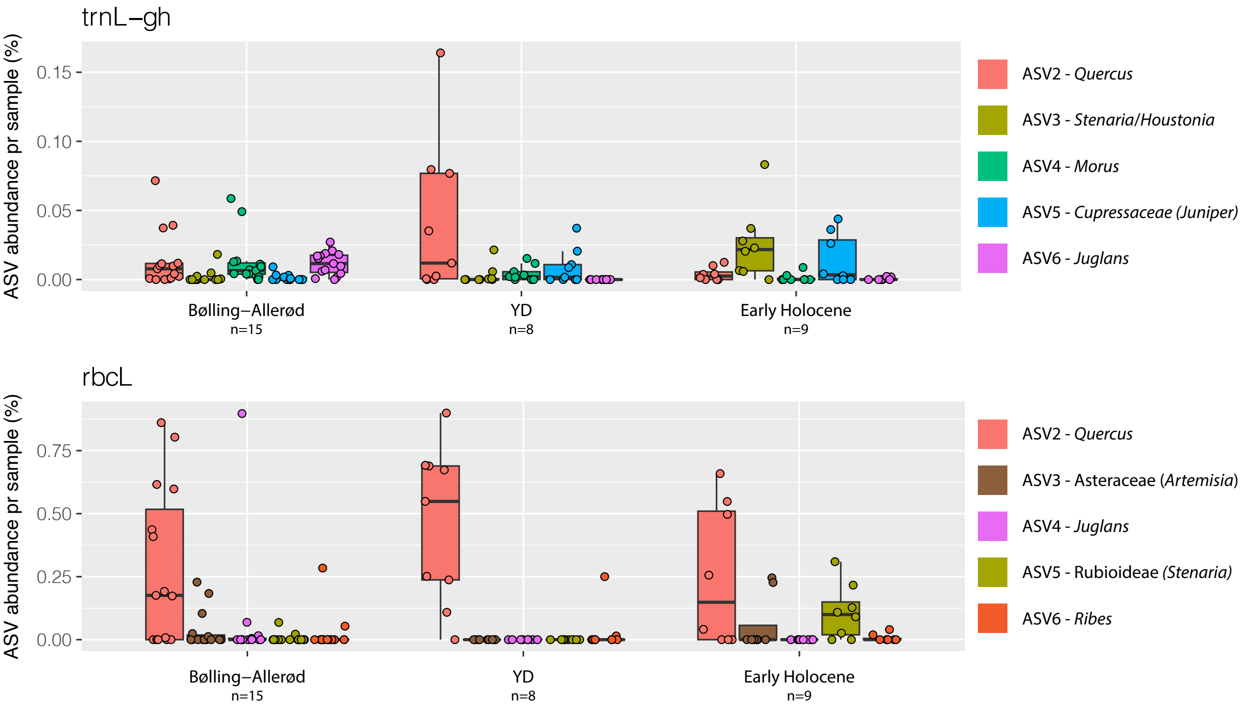

Supplementary Figure 6. Most common plant ASV's. Comparison of the top two-to-six most abundant ASV's for each plant assay excluding the highly abundant *Celtis* read (ASV1). Legend is sorted with the most abundant ASV at the top. The label is the taxonomic assignment of the given ASV. For each assay, biologically independent samples are compared between three time periods: Bølling-Allerød (n=15), YD (n=8) and Early Holocene (n=9). Center line: median. Box limits: upper and lower quartiles. Whiskers extends to 1.5xIQR (inter quartile range), no data points were excluded. Source data are provided as a Source Data file.

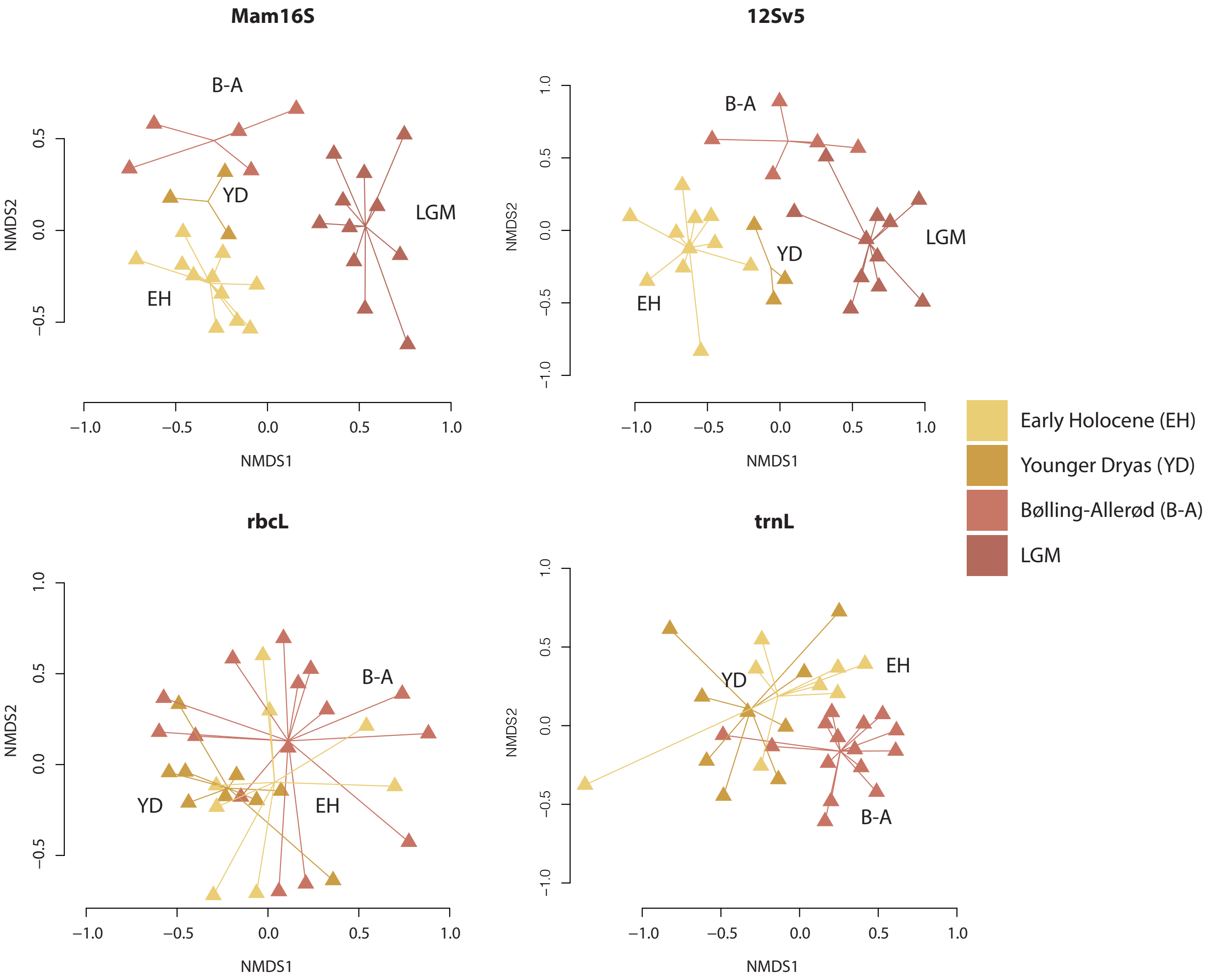

Supplementary Figure 7. Taxonomy-independent ordination analysis based on ASV diversity. The two upper panels represent bulk bone data analysed with mitochondrial assays targeting vertebrates (Mam16S and 12Sv5; subsampled to 7247 reads per sample), whereas the two lower panels represent sediment samples analysed with chloroplast assays targeting plants (rbcL and trnL; subsampled to 5374 reads per sample). As opposed to figures 3b and 4b in the main text which are based on the taxonomic record inferred from the DNA data, these ordination analyses are based on ASVs (amplicon sequence variants). Hence, this approach compares DNA sequences across samples without assigning these to taxa first. Accordingly, unknown sequences without matches in the database can be taken into account.

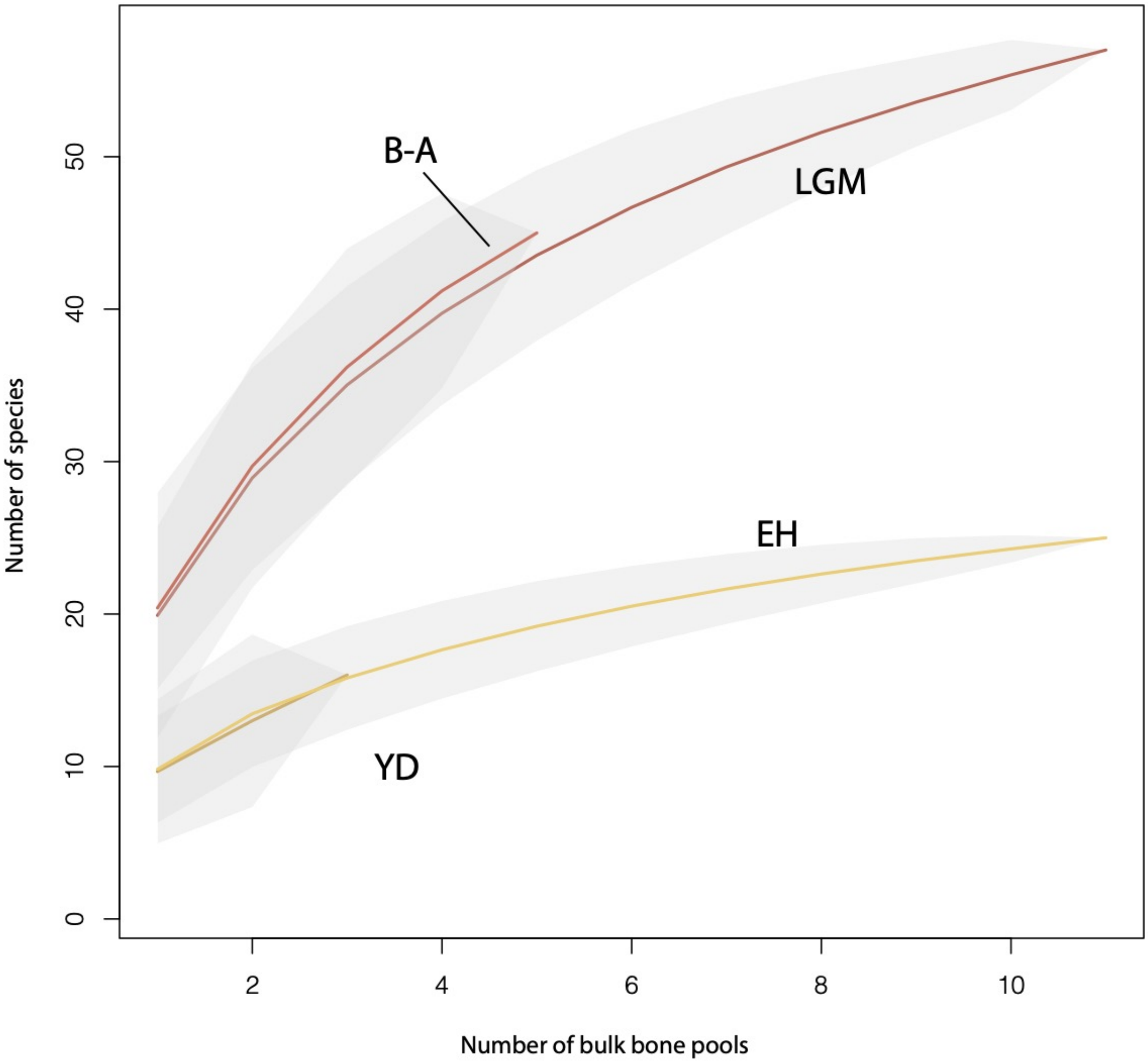

Supplementary Figure 8. Species accumulation curves for bulk bone data in each of the analysed time periods. One bulk bone pool represents 2x50 bone fragments.

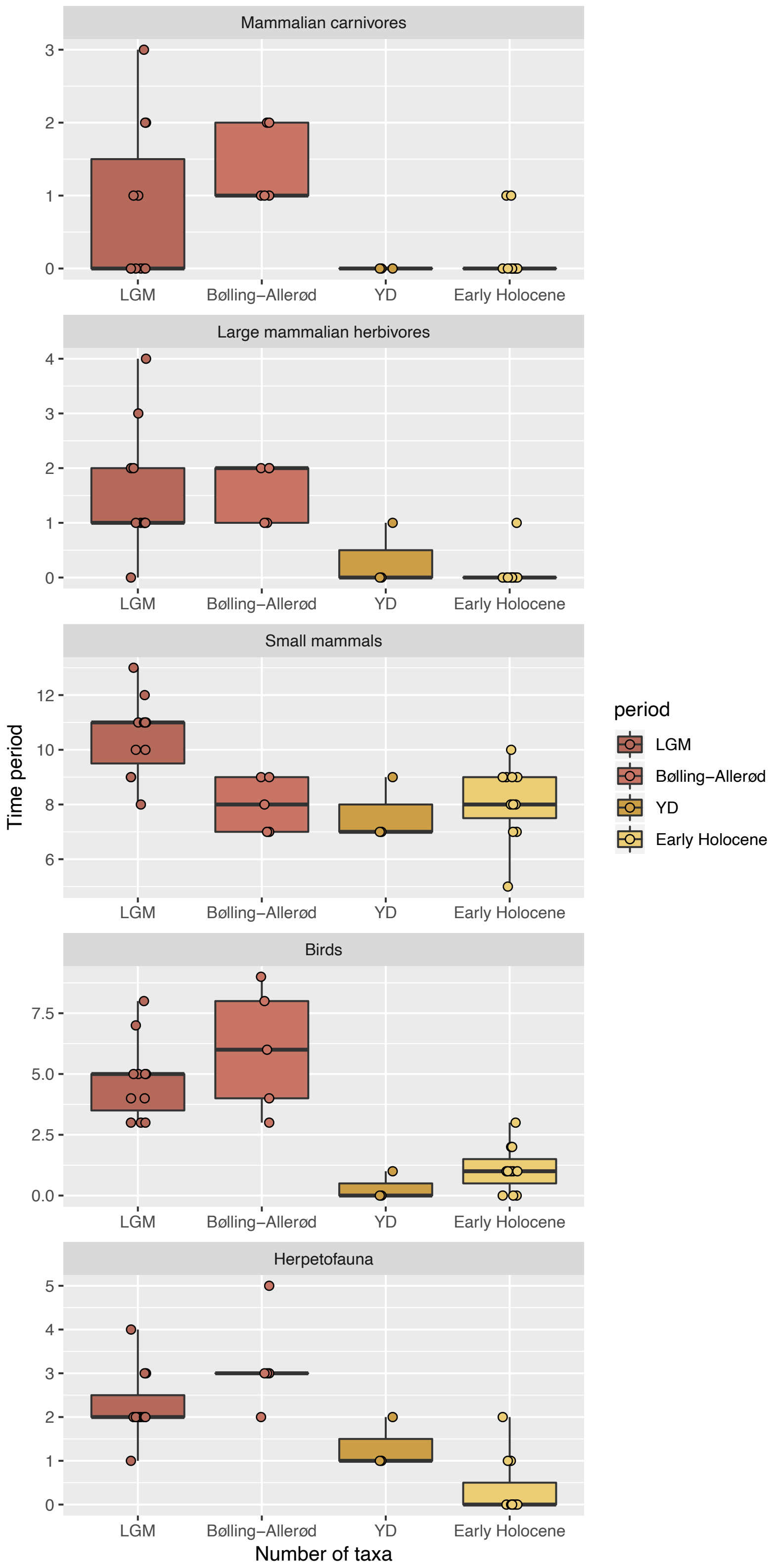

Supplementary Figure 9. Alpha diversity compared between different groups of species. Biologically independent samples compared between the four time periods: LGM (n=11), Bølling-Allerød (n=5), YD (n=3) and Early Holocene (n=11). Center line: median. Box limits: upper and lower quartiles. No data points were excluded. Source data are provided as a Source Data file.

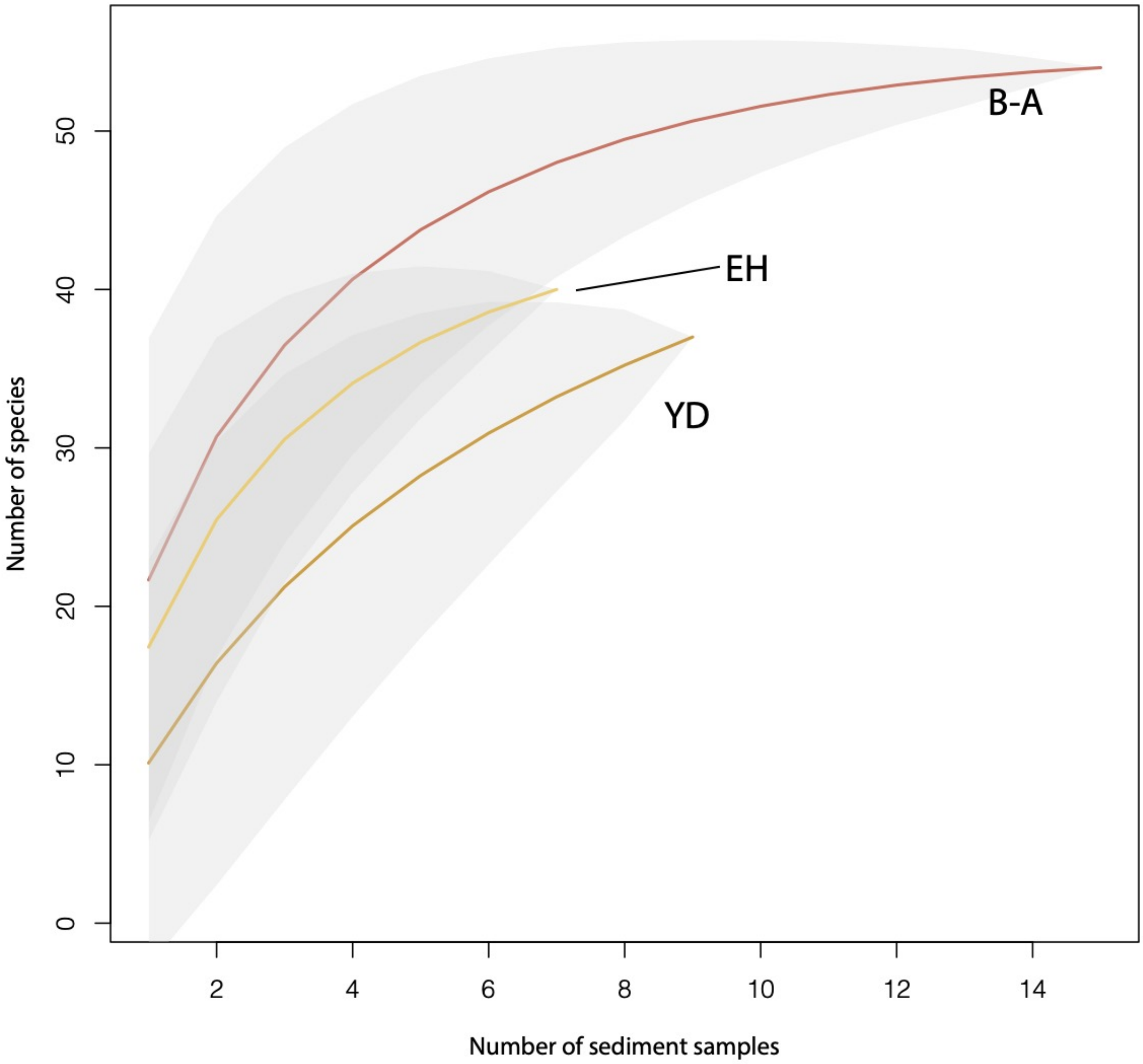

Supplementary Figure 10. Species accumulation curves for plant aDNA data in each of the analysed time periods.

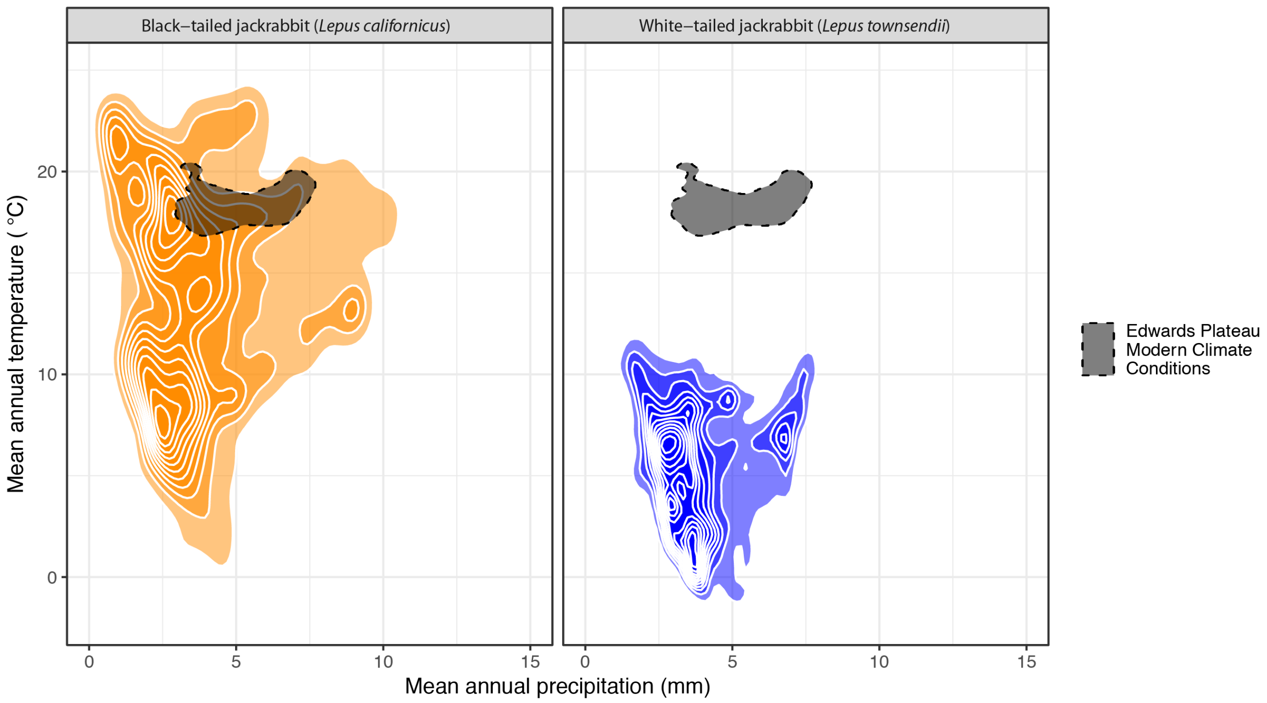

Supplementary Figure 11. *Lepus* spp. climate niche limits based on their modern habitats. Climatic niche limits levels were based on geographic ranges for relevant species from the *The IUCN Red List of Threatened Species* (https://www.iucnredlist.org) and precipitation and temperature data in a resolution of 10 minutes from WorldClim version 2 (http://worldclim.org/version2)[1].

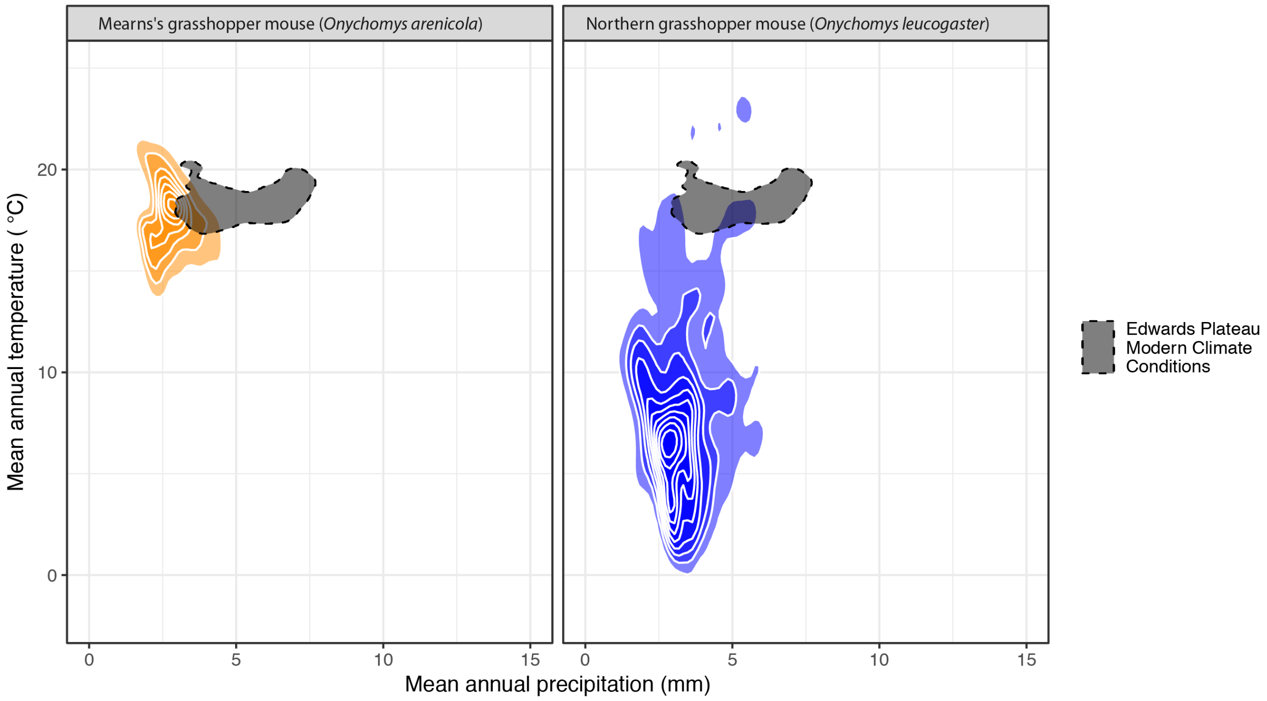

Supplementary Figure 12. *Onychomys* spp. climate niche limits based on their modern habitats. Climatic niche limits levels were based on geographic ranges for relevant species from the *The IUCN Red List of Threatened Species* (https://www.iucnredlist.org) and precipitation and temperature data in a resolution of 10 minutes from WorldClim version 2 (http://worldclim.org/version2)[1].

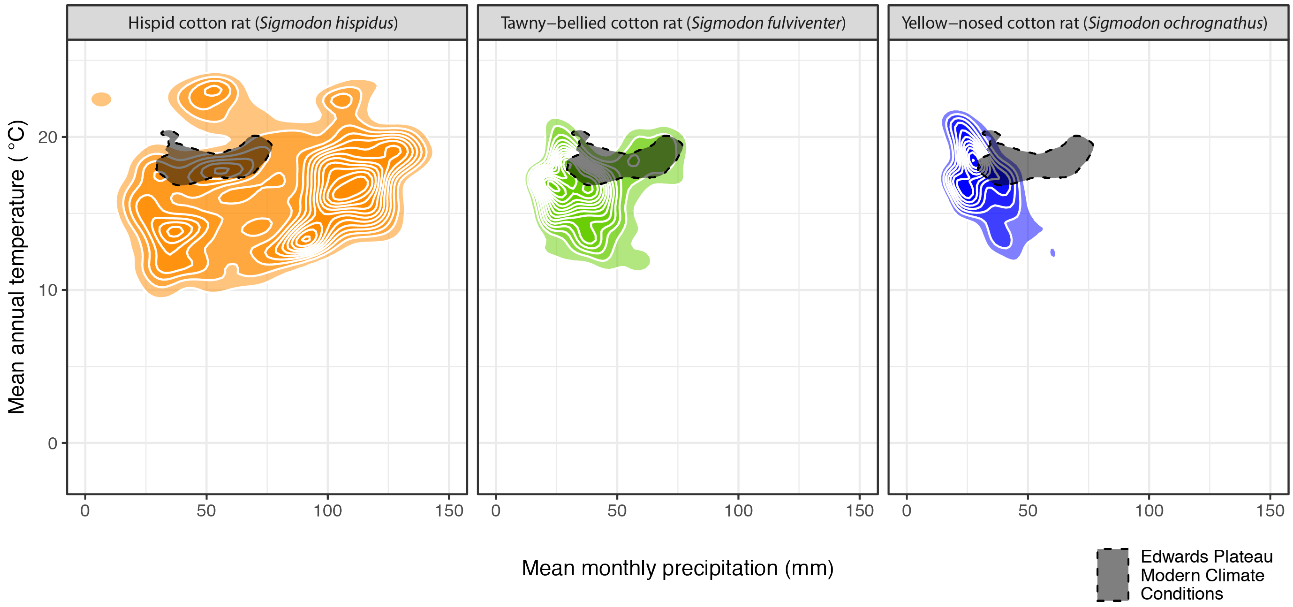

Supplementary Figure 13. *Sigmodon* spp. climate niche limits based on their modern habitats. Climatic niche limits levels were based on geographic ranges for relevant species from the *The IUCN Red List of Threatened Species* (https://www.iucnredlist.org) and precipitation and temperature data in a resolution of 10 minutes from WorldClim version 2 (http://worldclim.org/version2)[1].

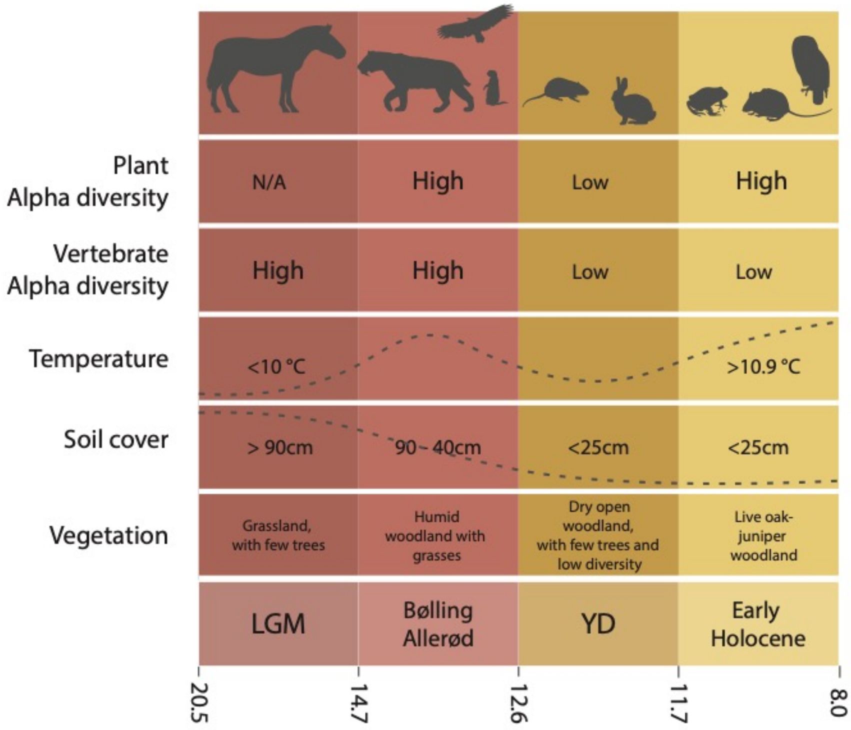

Supplementary Figure 14. Interpretation of past climate and ecology around Hall's Cave.

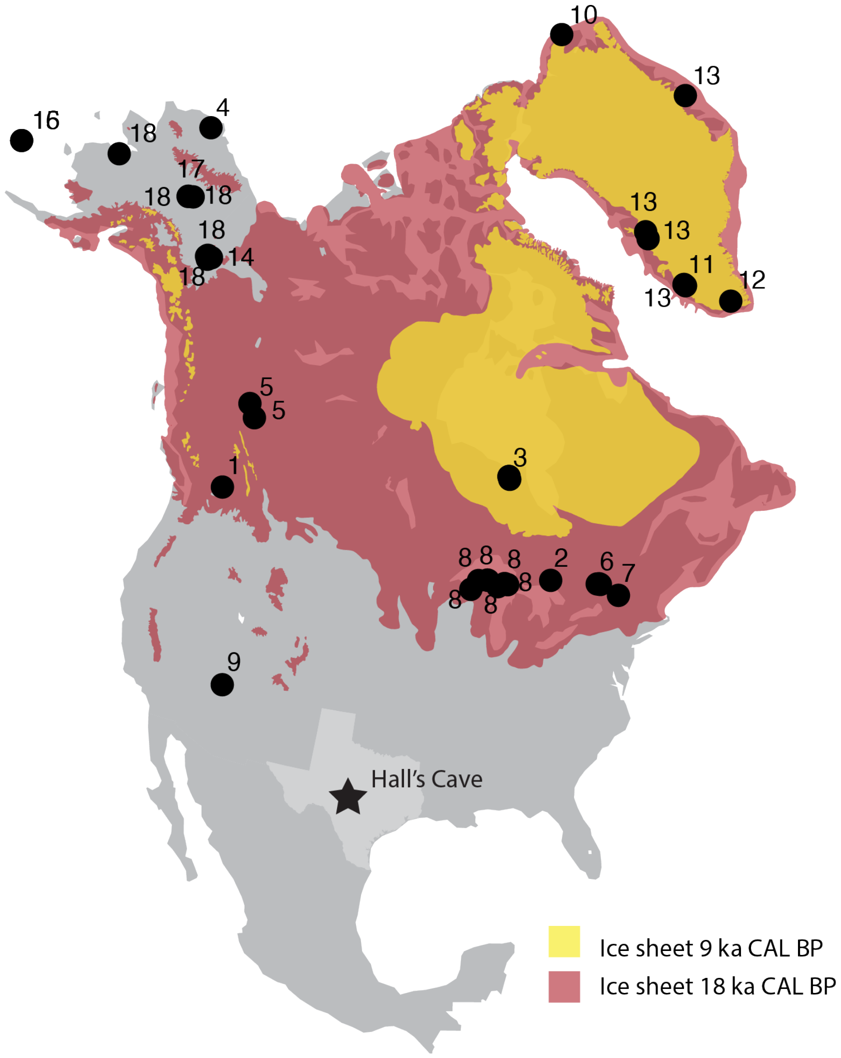

1 - Coolen and Overmann 1998
2 - Pollard et al. 2003
3 - Poulain et al. 2015
4 - Wooler et al. 2015
5 - Pedersen et al. 2016
6 - Pal et al. 2015
7 - Stager et al. 2015
8 - Anderson-Carpenter et al. 2011
9 - Hofreiter et al. 2003
10 - Epp et al. 2015
11 - Hebsgaard et al. 2009
12 - Pedersen et al. 2013
13 - Seersholm et al. 2016
14 - Porter et al. 2013
15 - Wang et al. 2017
16 - Graham et al. 2016
17 - Haile et al. 2009
18 - Willerslev et al. 2014

Supplementary Figure 15. *sed*aDNA studies from North America. Coolen and Overman 1998[2], Pollard et al. 2003[3], Poulain et al. 2015[4], Wooler et al. 2015[5], Pedersen et al. 2016[6], Pal et al. 2015[7], Stager et al. 2015[8], Anderson-Carpenter et al. 2011[9], Hofreiter et al. 2003[10], Epp et al. 2015[11], Hebsgaard et al. 2009[12], Pedersen et al. 2013[13], Seersholm et al. 2016[14], Porter et al. 2013[15], Wang et al. 2017[16], Graham et al. 2016[17], Haile et al. 2009[18], Willerslev et al. 2014[19]

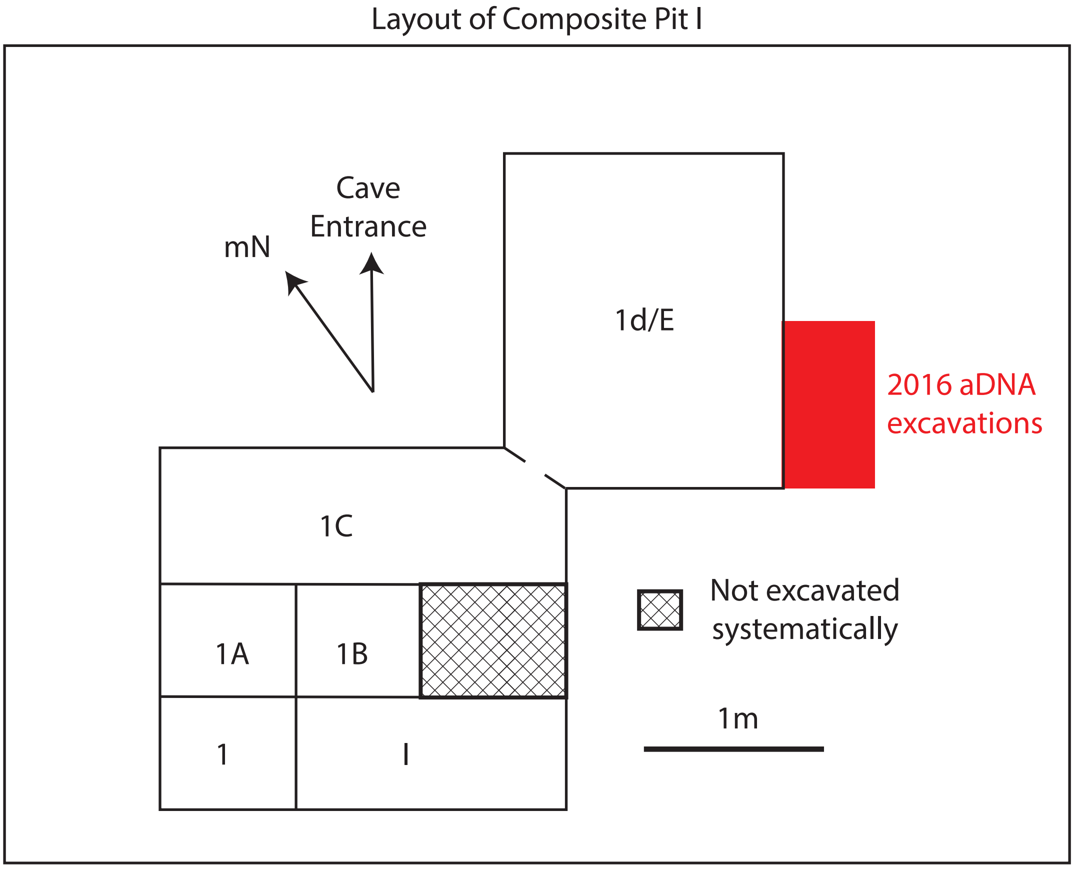

Supplementary Figure 16. Schematic layout of the existing pit. Samples for this study were excavated from the eastern face of excavation pit 1d/E (highlighted in red).

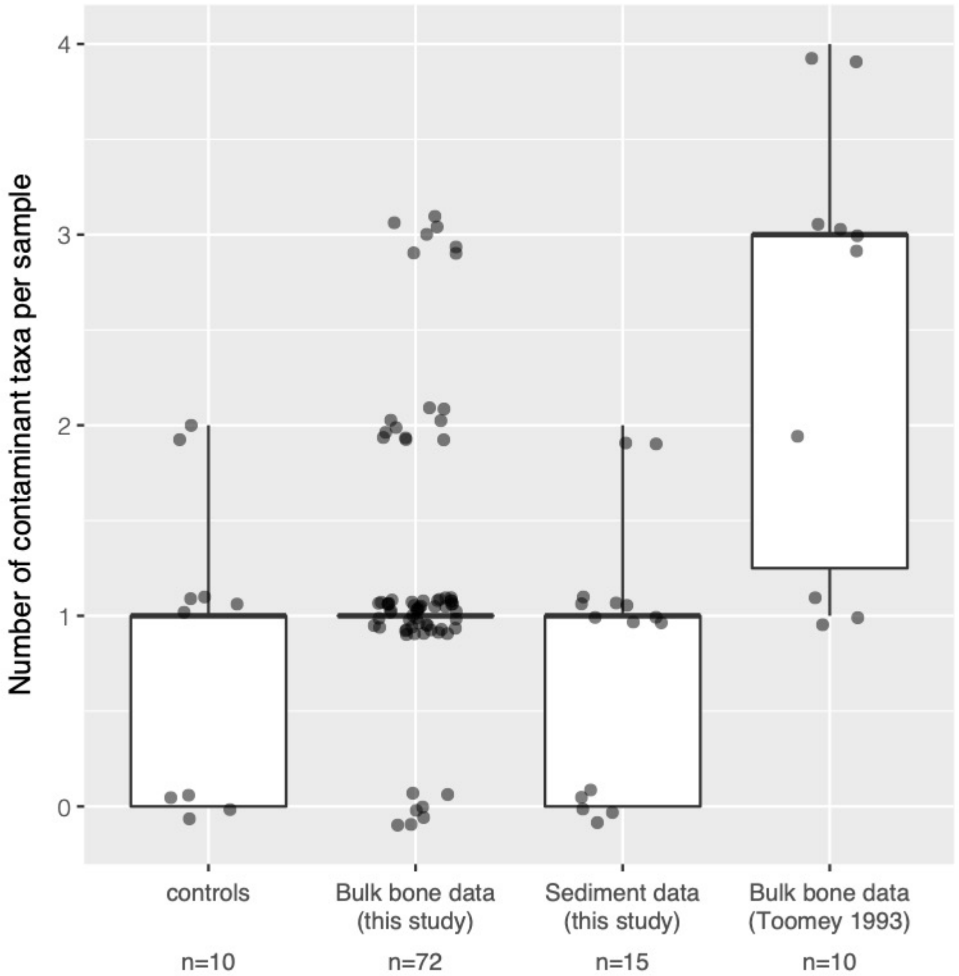

Supplementary Figure 17. Contamination. Number of contaminant taxa in samples excavated following ancient DNA guidelines (bulk bone data from this study, n=72, and sediment data from this study, n=15) compared with laboratory controls (n=10) and material that was excavated for morphological analyses by Toomey (n=10). Contaminant taxa include human (*Homo* sp.), dog/wolf (*Canis* sp.), sheep (*Ovis* sp.), cattle (*Bos* sp.), goat (*Capra* sp.), cat (*Felis* sp.), chicken (*Gallus gallus*) and pig (*Sus scrofa*). Center line: median. Box limits: upper and lower quartiles. Whiskers extends to 1.5xIQR (inter quartile range), no data points were excluded. Source data are provided as a Source Data file.

Supplementary Tables

Supplementary Table 1. Bulk bone sample information. cm BD_S: cm below datum (this study). cm BD_T: cm below datum established by Toomey. Elev. m. ASL: Elevation in meters above sea level. The last six samples represent large fragmentary fossils excavated by Toomey in 1993. As these samples were not excavated as part of this study there is no information on excavation interval or depth measured from the datum line of this study. Furthermore, the exact number of bones analysed for these samples is not known as they were recorded as 'approximately 100 bone fragments'. As 1C_240_245 was excavated from pit 1c and not composite pit 1d/E, it does not have a precise date. Similarly, sample 1E_350_355 (excavated from 352.5 cm BD_T) does not have precise dates as the earliest date from the age-depth model is from 347.5 cm BD_T.

Sample name	Number bones analysed	Excavation Interval	Depth (cm BD_T)	Median z (cm BD_T)	Median Elev. (m ASL)	Best modelled age (cal BP)	95% uncertainty ranges (cal BP)	Time period
**HCB1**	100	133,134	103-109	106	664.830	8,038	8,233-7,646	EH
**HCB2**	100	135	109-112	110.5	664.785	8,529	8,700-8,153	EH
**HCB3**	100	136	112-115	113.5	664.755	8,835	9,047-8,500	EH
**HCB4**	100	137	115-118	116.5	664.725	9,132	9,387-8,857	EH
**HCB5**	100	138	118-121	119.5	664.695	9,427	9,714-9,225	EH
**HCB6**	100	139	121-124	122.5	664.665	9,726	10,040-9,583	EH
**HCB7**	100	140	124-128	126	664.630	10,088	10,419-9,963	EH
**HCB8**	100	141	128-131	129.5	664.595	10,459	10,775-10,329	EH
**HCB9**	100	142	131-134	132.5	664.565	10,779	11,073-10,639	EH
**HCB10**	100	143	134-137	135.5	664.535	11,098	11,367-10,952	EH
**HCB11**	100	144	137-140	138.5	664.505	11,411	11,652-11,260	EH
**HCB12**	100	145	140-143	141.5	664.475	11,714	11,925-11,556	YD
**HCB13**	100	146	143-147	145	664.440	12,050	12,236-11,894	YD
**HCB14**	100	147	147-150	148.5	664.405	12,362	12,517-12,203	YD
**HCB15**	100	148, 149	150-156	153	664.360	12,710	12,821-12,538	B-A
**HCB16**	100	150,151	156-162	159	664.300	13,035	13,112-12,856	B-A
**HCB17**	100	152,153	162-169	165.5	664.235	13,188	13,272-13,067	B-A
**HCB18**	100	154,155	169-181	175	664.140	13,228	13,399-13,125	B-A
**HCB19**	100	156-162	181-204	192.5	663.965	13,937	14,251-13,747	B-A
**HCB20**	100	163-166	204-216	210	663.790	16,385	16,797-15,475	LGM
**HCB21**	100	167-168	216-222	219	663.700	17,360	17,759-16,421	LGM
**HCB22**	100	169-170	222-228	225	663.640	17,722	18,168-16,945	LGM
**HCB23**	100	171	228-231	229.5	663.595	17,892	18,356-17,316	LGM
**HCB24**	100	172	231-234	232.5	663.565	17,974	18,437-17,532	LGM
**HCB25**	100	173-174	234-240	237	663.520	18,072	18,515-17,797	LGM
**HCB26**	100	175-177	240-249	244.5	663.445	18,226	18,580-18,081	LGM
**HCB27**	100	178-184	249-271	260	663.290	18,592	18,757-18,454	LGM
**HCB28**	100	185-192	271-297	284	663.050	19,336	19,525-19,169	LGM
**HCB29**	75	193-199	297-319	308	662.810	19,908	20,062-19,716	LGM
**HCB30**	82	200-205	319-337	328	662.610	20,025	20,198-19,883	LGM
**1C_240_245**	~100	-	-	242.5	663.465	~18,000	N/A	LGM
**1D_235_240**	~100	-	-	237.5	663.515	18,082	18,520-17,821	LGM
**1D_265_270**	~100	-	-	267.5	663.215	18,811	18,978-18,670	LGM
**1D_270_275**	~100	-	-	272.5	663.165	18,968	19,142-18,818	LGM
**1D_345_350**	~100	-	-	347.5	662.415	20,185	20,375-19,990	LGM
**1E_350_355**	~100	-	-	352.5	662.365	~20,000	N/A	LGM

Supplementary Table 2. Primers

	Forward Primer	*Reverse Primer*	*Annealing Temp*	*ref*
12SV5	TAGAACAGGCTCCTCTAG	TTAGATACCCCACTATGC	57°C	Riaz et al. (2011) [20]
Mam16S	CGGTTGGGGTGACCTCGGA	GCTGTTATCCCTAGGGTAACT	57°C	Taylor (1996) [21]
rbcL	GGCAGCATTCCGAGTAACTCCTC	CGTCCTTTGTAACGATCAAG	52°C	Poinar et al. (1998) [22]
trnL-gh	GGGCAATCCTGAGCCAA	CCATTGAGTCTCTGCACCTATC	52°C	Taberlet et al. (2007) [23]

Supplementary Table 3. Sequencing counts for bulk bone samples. Roman numerals represent different PCR amplifications from the same extract.

sample name	12S						16S					
	Replicate A			Replicate B			Replicate A			Replicate B		
	raw count	filtered count	unique reads	raw count	filtered count	unique reads	raw count	filtered count	unique reads	raw count	filtered count	unique reads
**HCB1**	11588	6310	12	27849	13275	13	11980	7798	12	11752	7328	11
**HCB2**	11291	5547	12	12625	6312	12	19374	12121	12	14081	8261	13
**HCB2-II**	27077	14707	11	-	-	-	-	-	-	49550	33996	16
**HCB3**	9186	5339	14	12570	6946	7	14848	9054	18	14370	9027	13
**HCB3-II**	65125	35445	14	-	-	-	-	-	-	-	-	-
**HCB4**	15551	6906	14	9792	6195	7	29206	16682	18	9524	6572	13
**HCB5**	11684	6930	8	10335	5257	10	11759	7842	12	10935	6857	15
**HCB6**	9750	4429	8	10536	5616	6	13362	6583	15	10235	6364	10
**HCB7**	7247	3715	6	8207	4314	9	9952	4641	11	11747	6480	12
**HCB8**	14583	7330	10	9937	4894	8	12151	6442	13	13292	7644	14
**HCB9**	9056	5147	7	13690	7271	7	11786	7228	7	16648	8681	15
**HCB10**	12031	6077	9	10698	5222	13	13044	8661	9	12587	8350	12
**HCB11**	8353	4870	5	14034	6281	5	12616	9258	7	12547	7648	10
**HCB12**	10050	5073	3	10138	4713	10	10411	5718	14	10783	6716	10
**HCB13**	11359	5474	7	9492	5067	8	13160	5184	18	10596	6912	8
**HCB13-II**	97453	52180	9	-	-	-	88180	43808	19	-	-	-
**HCB14**	9006	3772	9	9942	5240	7	12111	6850	11	11280	7161	15
**HCB14-II**	-	-	-	84774	46142	11	-	-	-	-	-	-
**HCB15**	9113	5414	8	13124	7752	11	11408	7456	8	11470	6517	13
**HCB16**	7584	4711	10	9320	5604	17	11425	6937	10	11537	7662	16
**HCB16-II**	-	-	-	-	-	-	-	-	-	73817	48313	23
**HCB17**	12404	6042	25	10110	5697	16	15154	9768	13	13475	7981	16
**HCB17-II**	77789	38569	32	67824	33587	21	59596	33452	26	-	-	-
**HCB18**	8867	5762	14	9863	5845	13	13496	9193	12	14376	9587	7
**HCB18-II**	-	-	-	-	-	-	110285	66318	18	64693	41831	15
**HCB19**	11347	5994	16	11514	5550	14	19386	13255	19	11917	6433	18
**HCB19-II**	-	-	-	30271	13221	31	-	-	-	-	-	-
**HCB20**	11550	6601	14	9516	5322	20	14081	7996	17	10797	6181	12
**HCB21**	9292	5329	15	9599	4808	18	11141	6919	12	14767	9721	23
**HCB22**	9138	4462	20	9660	4423	15	13200	7675	20	11484	6817	18
**HCB22-II**	106476	46642	25	-	-	-	-	-	-	-	-	-
**HCB23**	39932	15074	12	43296	21742	11	41604	20295	27	36174	23411	13
**HCB24**	14235	6398	15	17246	9713	20	14957	9502	17	13683	8160	20
**HCB25**	13018	6053	12	12122	6733	17	12076	6621	18	15095	10557	23
**HCB26**	12872	6128	22	12975	7731	18	16774	10426	20	14306	7888	20
**HCB27**	13603	6629	18	12810	6566	18	8907	5039	15	9184	5300	14
**HCB28**	9890	5318	21	12669	7051	15	10392	6868	16	14700	9986	11
**HCB29**	11760	6586	14	9287	5906	7	15918	9906	16	10307	6459	9
**HCB30**	10584	5356	11	11798	6378	9	7832	4388	12	8134	4119	11
**1D_235_240**	16222	8608	38	-	-	-	11775	7501	26	-	-	-
**1D_235_240_I**	44926	18815	39	-	-	-	63171	39504	53	-	-	-
**1D_235_240_II**	83173	34926	40	-	-	-	70026	43753	44	-	-	-
**1C_240_245**	12850	5751	23	-	-	-	14594	8541	22	-	-	-
**1C_240_245_I**	98430	39486	32	-	-	-	96744	56324	35	-	-	-
**1C_240_245_II**	61986	25727	31	-	-	-	86763	47850	43	-	-	-
**1D_265_270**	10669	5995	19	-	-	-	13465	9607	17	-	-	-
**1D_270_275**	12301	6328	23	-	-	-	14636	8663	17	-	-	-
**1D_345_350**	16639	8181	20	-	-	-	11829	6684	13	-	-	-
**1E_350_355**	17160	8974	15	-	-	-	12037	6476	12	-	-	-

Supplementary Table 4. Sediment sample information. cm BD_S: cm below datum (this study). cm BD_T: cm below datum established by Toomey. Elev. m. ASL: Elevation in meters above sea level.

Sample name	Sequence	Depth (cm BD_T)	Elevation (m. ASL)	Best modelled age (cal BP)	95% uncertainty ranges (cal BP)	Time period
**HCS1**	A	118.5	664.705	9,328	9,606-9,101	Early Holocene
**HCS2**	A	121.5	664.675	9,626	9,931-9,464	Early Holocene
**HCS3**	A	124.5	664.645	9,931	10,258-9,807	Early Holocene
**HCS4**	A	127.5	664.615	10,246	10,574-10,121	Early Holocene
**HCS5**	A	130.5	664.585	10,566	10,872-10,431	Early Holocene
**HCS6**	A	133.5	664.555	10,886	11,167-10,743	Early Holocene
**HCS7**	A	136.5	664.525	11,203	11,462-11,055	Early Holocene
**HCS8**	A	139.5	664.495	11,513	11,749-11,360	Early Holocene
**HCS9**	A	142.5	664.465	11,812	12,016-11,656	YD
**HCS10**	A	145.5	664.435	12,097	12,278-11,940	YD
**HCS11**	A	148.5	664.405	12,362	12,517-12,203	YD
**HCS12**	A	151.5	664.375	12,602	12,727-12,433	Bølling-Allerød
**HCS13**	A	154.5	664.345	12,809	12,909-12,631	Bølling-Allerød
**HCS14**	A	157.5	664.315	12,972	13,054-12,788	Bølling-Allerød
**HCS15**	A	160.5	664.285	13,087	13,160-12,919	Bølling-Allerød
**HCS16**	A	163.5	664.255	13,159	13,235-13,016	Bølling-Allerød
**HCS17**	A	166.5	664.225	13,198	13,287-13,080	Bølling-Allerød
**HCS18**	A	169.5	664.195	13,215	13,329-13,108	Bølling-Allerød
**HCS19**	A	172.5	664.165	13,222	13,364-13,121	Bølling-Allerød
**HCS20**	A	175.5	664.135	13,230	13,411-13,127	Bølling-Allerød
**HCS22**	B	142	664.47	11,763	11,971-11,607	YD
**HCS24**	B	144	664.45	11,957	12,150-11,798	YD
**HCS26**	B	146	664.43	12,142	12,319-11,986	YD
**HCS28**	B	148	664.41	12,319	12,482-12,162	YD
**HCS29**	B	149	664.4	12,404	12,552-12,243	YD
**HCS30**	B	150	664.39	12,486	12,624-12,321	YD
**HCS31**	B	152	664.37	12,639	12,760-12,468	Bølling-Allerød
**HCS32**	B	153	664.36	12,710	12,821-12,538	Bølling-Allerød
**HCS34**	B	155	664.34	12,839	12,934-12,661	Bølling-Allerød
**HCS36**	B	157	664.32	12,948	13,033-12,766	Bølling-Allerød
**HCS38**	B	159	664.3	13,035	13,112-12,856	Bølling-Allerød
**HCS40**	B	161	664.28	13,101	13,176-12,936	Bølling-Allerød

Supplementary Table 5. Sequencing counts for sediment samples and sediment blanks.

sample name	trnL			rbcL		
	raw count	filtered count	unique reads	raw count	filtered count	unique reads
**HCS1**	49255	43318	9	124868	47150	4
**HCS2**	19060	15537	21	19428	9835	6
**HCS3**	75316	68112	4	29872	1493	1
**HCS4**	13210	11660	23	9088	3761	6
**HCS5**	99042	84209	24	101810	55341	6
**HCS6**	18650	15808	28	22558	13025	11
**HCS7**	91916	80524	16	89249	41080	4
**HCS8**	19284	15915	13	16380	4337	3
**HCS9**	52371	47523	6	110757	82202	2
**HCS10**	14891	12502	9	17786	7731	3
**HCS11**	61620	51300	9	41376	21793	5
**HCS12**	23592	19911	31	18598	10703	7
**HCS13**	48943	41471	13	41260	17170	8
**HCS14**	20337	16091	31	22694	9976	7
**HCS15**	44386	33599	21	65540	37276	3
**HCS16**	23188	16321	24	20799	2663	7
**HCS17**	42333	35188	19	35090	11171	3
**HCS18**	18465	12845	28	14304	6771	8
**HCS19**	43175	36378	22	45101	17095	6
**HCS20**	18394	14940	35	19041	7264	6
**HCS22**	5519	4385	24	9682	7066	7
**HCS24**	17276	14503	15	13165	9920	2
**HCS26**	18148	14042	8	5374	4538	3
**HCS28**	16721	14618	9	16754	12382	4
**HCS29**	15908	13860	11	16238	10028	3
**HCS30**	17858	15082	20	21668	14544	4
**HCS31**	17576	13610	33	16315	10753	7
**HCS32**	18801	16587	22	13563	9315	7
**HCS34**	17165	14783	29	16292	8606	10
**HCS36**	13742	9077	25	13314	5366	5
**HCS38**	28800	22278	38	21367	14530	5
**HCS40**	14044	9173	6	13491	10649	3
**EB1**	5	-	-	7323	3689	1
**EB2**	12379	9073	1	12167	8057	1
**EB3**	9	9	1	49323	40643	1

Supplementary Table 6. Mammal species identified. Only lowest taxonomic nodes detected are shown. Some taxonomic nodes were either upgraded (∧ ; family -> species) or downgraded (∨ ; species -> family) based on database coverage and species distribution. EH: Early Holocene, YD: Younger Dryas, BA: Bølling-Allerød, LGM: Last Glacial Maximum. (x) signifies that the genus was identified before, but that the taxa could not be resolved to species level by morphology. * Common laboratory contaminants. C: carnivore (of terrestrial vertebrates), I: Insectivore, H: Herbivore. Dietary preference was sourced from: https://animaldiversity.org.

Taxon	Common name	Found at HC before?	Extinct?	Diet	EH n=11	YD n=3	BA n=5	LGM n=17	Sed n=15
Lepus californicus	Black-tailed jackrabbit	X	-	H	8	-	4	-	-
Lepus townsendii	White-tailed jackrabbit	-	-	H	-	1	2	17	-
Sylvilagus sp.	Cottontail rabbit	X	-	H	9	3	5	17	1
Geomys texensis∧	Central Texas pocket gopher	(x)	-	H	-	-	-	1	-
Thomomys bottae	Botta's pocket gopher	(x)	-	H,I	-	-	-	1	-
Chaetodipus sp.	Pocket mouse	X	-	H,I	11	3	5	5	4
Synaptomys cooperi	Bog lemming	X	-	H,I	-	-	-	4	-
Microtus sp.∨	Vole	X	-	H,I	3	2	4	14	1
Neotoma floridana	Eastern woodrat	(x)	-	H,I	10	3	5	16	3
Neotoma lepida	Desert woodrat	(x)	-	H	3	-	3	6	-
Neotoma leucodon	White-toothed woodrat	(x)	-	H	7	2	-	3	-
Neotoma micropus	Southern Plains woodrat	(x)	-	H	5	-	-	-	-
Onychomys arenicola	Mearns's grasshopper mouse	(x)	-	I	9	-	-	-	2
Onychomys leucogaster	Northern grasshopper mouse	X	-	I	2	3	2	11	1
Peromyscus sp.	Deer mouse	X	-	H,I	11	3	4	17	3
Sigmodon sp.∨	Cotton rat	X	-	H,I	10	-	-	-	-
Cynomys sp.	Prairie dog	X	-	H,I	-	-	1	10	-
Ictidomys tridecemlineatus	Thirteen-lined ground squirrel	(x)	-	H,I	-	-	2	12	-
Marmota sp.	Marmot	-	-	H	-	-	1	5	-
Tamias canipes∧	Gray-footed chipmunk	-	-	H,I	-	-	-	1	-
Platygonus compressus	Flat-headed peccary	X	X	H	-	-	-	4	-
Antilocapra americana	Pronghorn	X	-	H	-	-	-	4	-
Bison sp.	Bison	X	-	H	-	-	3	5	-
Bootherium bombifrons	Helmeted muskox	-	X	H	-	-	-	3	-
Odocoileus sp.	Deer	X	-	H	1	1	2	7	-
Camelops hesternus	Yesterday’s camel	-	X	H	-	-	-	1	-
Unknown *Camelidae*∨	Camel	NA	X	H	-	-	-	1	-
Canis latrans	Coyote	X	-	C,I,H	1	-	-	4	-
Urocyon cinereoargenteus∧	Gray fox	X	-	C,H	-	-	2	-	-
Vulpes vulpes	Red fox	X	-	C,I,H	-	-	-	1	-
Mephitis sp.∨	Skunk	X	-	C,I,H	-	-	1	6	-
Spilogale sp.∨	Spotted skunk	X	-	C,I,H	-	-	4	-	-
Mustela frenata	Long-tailed weasel	X	-	C	-	-	-	1	-
Mustela nivalis	Least weasel	-	-	C,I	-	-	-	2	-
Procyon lotor	Racoon	X	-	C,I,H	1	-	-	-	-
Arctodus sp.∨	Short-faced bear	-	X	C	-	-	-	1	-
Ursus americanus	American black bear	X	-	C,I,H	-	-	-	4	-
Smilodon sp.∨	Saber-toothed cat	X	X	C	-	-	-	1	-
Lynx rufus	Bobcat	X	-	C	-	-	-	-	2
Panthera onca	Jaguar	X	-	C	-	-	-	1	5
Blarina carolinensis/hylophaga	Shrew	X	-	I	-	-	-	5	-
Scalopus aquaticus	Eastern mole	X	-	I	-	1	-	2	-
Notiosorex sp.	Shrew	X	-	I	-	-	-	-	1
Equus caballus/lambei/scotti∧	Caballine equid	X	X	H	-	-	1	5	-
Haringtonhippus francisci	New World stilt-legged horse	-	X	H	-	-	4	-	-
Tapirus sp.	Tapir	-	X	H	-	-	-	1	-
Tadarida brasiliensis	Mexican free-tailed bat	X	-	I	-	1	-	1	1
Eptesicus fuscus	Big brown bat	X	-	I	-	-	-	11	-
Myotis sp.	Mouse-eared bat	X	-	I	2	1	5	7	-
Perimyotis subflavus∧	Tricolored bat	X	-	I	-	1	1	4	-
*Homo sapiens**	Human				11	2	5	16	5
*Sus scrofa**	Domestic Pig				1	-	-	3	-
Bos sp.*	Cattle				1	1	2	1	1
Capra sp.*	Goat				1	2	4	-	2
Ovis sp.*	Sheep				1	-	-	-	-
Canis sp.*	Dog/wolf				2	-	-	4	-
Felis sp.*	Cat				-	-	-	2	-

Supplementary Table 7. Bird species identified. Only lowest taxonomic nodes detected are shown. Some taxonomic nodes were either upgraded (∧ ; family -> species) or downgraded (∨ ; species -> family) based on database coverage and species distribution. EH: Early Holocene, YD: Younger Dryas, BA: Bølling-Allerød, LGM: Last Glacial Maximum. (x) signifies that the genus was identified before, but that the taxa could not be resolved to species level by morphology. Habitat type is from https://www.allaboutbirds.org. Habitat type 'lakes and ponds', 'shorelines' and 'marshes' are all categorized as 'wetlands'. * Common laboratory contaminants.

taxon	Common name	Found at HC before?	Habitat type	EH (n=11)	YD (n=3)	BA (n=5)	LGM (n=17)
Anatidae	Ducks, geese, swans	X	Wetlands	-	-	2	10
Mergus sp.	Merganser	-	Wetlands	-	-	-	1
Anas sp.	Dabling ducks, pintails, teals, mallard	-	Wetlands	-	-	2	-
Aythya sp.	Diving ducks	-	Wetlands	-	-	1	-
Tadorna sp.	Shell ducks	-	Wetlands	-	-	-	3
Chordeiles minor	Common nighthawk	-	Grassland	-	-	-	1
Charadrius sp.$^{\vee}$	Plover	-	Grassland/Wetlands	-	-	-	1
Pluvialis sp.	Plover	-	Grassland/Wetlands	-	-	-	6
Leucophaeus sp.$^{\vee}$	Gull	-	Wetlands	-	-	-	1
Onychoprion sp.	Tern	-	Wetlands	-	-	-	5
Bartramia longicauda	Upland sandpiper	-	Grassland	-	-	1	7
Calidris sp.$^{\vee}$	Sandpiper	-	Grassland/Wetlands	-	-	-	2
Limosa sp.	Godwit	-	Wetlands	-	-	-	1
Gymnogyps sp.	Vulture, Condor	-	Scrub	-	-	1	1
Buteo swainsoni	Swainsons hawk	-	Grassland	1	-	-	1
Caracara cheriway	Northern crested caracara	-	Scrub	-	-	1	1
Callipepla sp.	Crested quail	(x)	Scrub/Grassland	-	-	1	-
Colinus virginianus$^{\wedge}$	Northern bobwhite	(x)	Grassland/open woodlands	3	1	4	3
Meleagris gallopavo	Wild turkey	X	Open woodland	3	-	5	1
Tympanuchus sp.	Prairie-chickens	X	Grassland	-	-	-	13
Porzana Carolina	Sora	-	Wetlands	-	-	-	1
Passeriformes	Passerines	X	N/A	1	1	5	15
Turdus sp.$^{\wedge}$	True trush	X	Open woodland	-	-	-	1
Sylvioidea	Swallows, larks	-	Wetlands/open woodlands	-	-	-	1
Passeroidea	Sparrow, finch, grackle	-	Grassland/open woodlands	1	-	-	-
Eremophila alpestris	Horned lark	-	Grassland	-	-	1	3
Bombycilla sp.	Waxwing	-	Forests/open woodlands	-	-	-	1
Corvus sp.$^{\wedge}$	Crows	X	Forests/open woodlands	1	-	2	3
Loxia sp.$^{\vee}$	Crossbill	-	Forests	-	-	1	-
Dumetella carolinensis$^{\wedge}$	Gray catbird	-	Open woodlands	-	-	-	1
Junco sp.$^{\vee}$	Junco	-	Forests/open woodlands	-	-	1	-
Sayornis sp.$^{\vee}$	Phoebe	X	Grassland/open woodlands	-	-	1	-
Tyrannus sp.	Kingbird	-	Grassland/open woodlands	-	-	-	2
Colaptes auratus	Northern flicker	-	Open woodlands	-	-	-	1
Podilymbus sp.$^{\vee}$	Grebe	-	Wetlands	-	-	1	1
Tyto alba	Barn owl	-	Grassland	2	-	-	-
Gallus gallus$^{*\wedge}$	Chicken	-	-	1	-	1	1

Supplementary Table 8. Reptiles, amphibians and fish species identified. Only lowest taxonomic nodes detected are shown. Some taxonomic nodes were either upgraded (∧ ; family -> species) or downgraded (∨ ; species -> family) based on database coverage and species distribution. EH: Early Holocene, YD: Younger Dryas, BA: Bølling-Allerød, LGM: Last Glacial Maximum.

taxon	Common name	Found at HC before?	EH (n=11)	YD (n=3)	BA (n=5)	LGM (n=17)
Testudinidae	Tortoise	-	-	1	2	-
Crotalus atrox^	Western diamondback rattlesnake	(x)	-	-	1	-
Crotalus viridis	Prairie rattlesnake	(x)	-	-	-	1
Cottus sp.	Sculpins	-	-	-	1	-
Ameiurus sp.ˇ	Bull heads	-	-	-	-	1
Scaphiopus couchii	Couch's spadefoot toad	X	-	-	3	-
Spea sp.ˇ	Western spadefoot toads	-	-	-	-	6
Anaxyrus cognatus	Great Plains toad	-	-	-	2	4
Anaxyrus woodhousii	Woodhouse's toad	X	1	-	2	10
Craugastor augusti^	Barking frog	X	3	3	4	2
Hyla sp.	Frog	-	-	-	1	-
Pseudacris clarkii^	Spotted chorus frog	(x)	-	-	1	-
Rana berlandieri^	Rio Grande leopard frog	X	-	-	3	4
Ambystoma sp.	Mole salamander	X	-	-	-	10

Supplementary Table 9. Vertebrate sequencing counts for Sediment samples and bulk bone blanks. Latin letters (A,B) specify separate extractions, whereas Roman numerals represent different PCR amplifications from the same extract. EB: Extraction blank, PB: PCR blank.

	**12S**			**16S**		
**sample name**	raw count	filtered count	unique reads	raw count	filtered count	unique reads
**HCS1**	54917	39758	1	59801	40410	8
**HCS5**	161389	106454	5	65162	40925	11
**HCS7**	115498	73540	7	52773	37481	19
**HCS11_A**	-	-	-	6091	5043	10
**HCS11_B**	53471	27146	1	62212	50869	5
**HCS12**	-	-	-	4843	4254	2
**HCS13_A_I**	-	-	-	5292	4557	7
**HCS13_A_II**	88727	38346	1	53326	42851	3
**HCS13_A_III**	56110	22605	2	65581	53687	2
**HCS13_B**	51	-	-	67326	55309	1
**HCS14**	-	-	-	7074	5932	6
**HCS15**	-	-	-	5152	4184	4
**HCS15**	345	-	-	63191	47204	1
**HCS17**	43	-	-	59992	46606	1
**HCS19**	37348	27032	1	-	-	-
**EB1**	-	-	-	12	-	-
**EB2**	-	-	-	-	-	-
**EB3**	3	-	-	12	-	-
**EB4**	-	-	-	-	-	-
**EB5**	10352	8242	1	10432	9462	1
**EB6**	-	-	-	3824	3171	3
**EB7**	138	-	-	37097	31510	1
**PB1**	3	-	-	39	30	2
**PB2**	-	-	-	8018	6802	2
**PB3**	63625	38912	2	11757	9266	2

Supplementary Table 10. Plant taxa identified. Only lowest taxonomic nodes detected are shown. Some taxonomic nodes were either upgraded (∧ ; family -> species) or downgraded (∨ ; species -> family) based on database coverage and species distribution. EH: Early Holocene, YD: Younger Dryas, BA: Bølling/Allerød.

Order	taxa	Common name	most likely candidate	Family detected by pollen?	Growth form	EH (n=8)	YD (n=9)	BA (n=15)
Apiales	*Cryptotaenia/Sanicula*	Honeywort, snakeroot		X	forb	-	-	10
	Apioideae	Umbellifers		X	forb	2	-	5
	Scandicinae	Sweet cicely	*Osmorhiza*	X	forb	4	1	11
Asterales	Anthemideae	Sagebrush	*Artemisia*	X	forb	5	1	15
	Aphanostephus	Dozedaisy		X	forb	-	3	1
	Thelesperma	Greenthread		X	forb	2	-	5
	Cirsium/Euonymus	Thistle	Cirsium	X	forb	-	1	5
Cupressales	*Cupressaceae*	Juniper	*Juniperus*	X	tree	5	5	5
Dipsacales	*Symphoricarpos*	Snowberry		-	shrub	-	1	6
Ericales	Sapotaceae	Milkwoods		-	tree	3	1	5
Malpighiales	*Euphorbia/Parthenocissus/Ambrosia*	Spurges	*Euphorbia*	X	tree/shrub	-	1	2
Fabales	*Gleditsia/Gymnocladus*	Honey locust/coffeetree		X	tree	3	2	12
	Mimosa	Sensitive plant		X	-	5	2	2
	Cercis	Red bud		X	tree	2	2	-
	Sophora	Sophora		X	tree/shrub	2	-	1
	Vicia	Vetches		X	herb	2	3	4
Cucurbitales	*Cucurbitaceae*	Cucurbits		-	forb	3	-	3
Fagales	*Juglans*	Walnut		X	tree	2	-	15
	Quercus	Oak		X	tree	6	8	13
Gentianales	*Galium*	Bedstraw		X	forb	4	-	8
	Stenaria/Houstonia	Diamond Flowers	*Stenaria*	X	forb	7	4	5
Lamiales	Oleeae	Ash	*Fraxinus*	X	tree	1	1	12
	Plantago	Plantain		-	forb	1	2	2
	Salvia	Sage		X	forb/shrub	1	3	7
Myrtales	Onagreae	Evening primroses	Oenothera	X	forb	-	1	2
Rosales	*Celtis*	Hackberry		X	tree	8	9	15
	Frangula	Buckthorn		X	shrub	-	1	2
	Parietaria	Pellitory		X	forb	1	1	3
	Morus	Mulberry		X	tree	2	6	13
Sapindales	*Toxicodendron* (Rhus complex)	Poison ivy		X	tree/shrub	2	5	14
	Ungnadia	Mexican buckeye		X	tree/shrub	4	-	-
Saxifragales	*Ribes*	Currant		X	tree/shrub	5	3	12
Solanales	*Solanoideae*	Nightshades		X	-	7	4	14
	Physalis	Ground cherries		X	forb	3	2	-
Vitales	*Cissus*	Treebine		X	vine	6	3	3
	Vitaceae	Grape family		X	tree/vine	-	-	7
Commelinales	*Commelina*	Dayflowers		-	forb	3	-	1
	Tradescantia	Spiderwort		-	forb	-	1	6
Poales	*Carex/Eleocharis*	Sedges		X	gramminoid	-	1	2
	PACMAD clade	-		X	gramminoid	4	1	12
	Bouteloua	Buffalo grass		X	gramminoid	-	-	3
	Stipeae	Needle grass		X	gramminoid	-	2	6
	Triticeae	-		X	gramminoid	1	2	10
	Hordeinae	-		X	gramminoid	1	-	4
	Melica	Melic grass		X	gramminoid	3	1	5

Supplementary Table 11. Primary radiocarbon dates from Cooke et al. 2003. *Samples were not included in the final age-depth model.

TMM catalog number	Depth (cm BD_T)	Median z (cm BD_T)	Material	C14 age (y BP)	Uncertanty (+/- σ)	Cal. max.* (y BP)	Cal. min.* (y BP)
41229-12115	15-20	17.5	gelatin	1500	60	1520	1300
41229-12118	25-30	27.5	gelatin	2330	60	2750	2150
41229-12083	51	51	charcoal	3190	70	3580	3240
41229-12117	60-65	62.5	gelatin	4000	60	4850	4250
41229-12099	76-78	77	humins	5400	70	6310	5990
41229-12162	90-95	92.5	gelatin	5320	60	6280	5930
41229-12164	105-110	107.5	gelatin	7700	80	8640	8350
41229-12166	120-125	122.5	gelatin	8630	60	9780	9490
41229-12080	145-150	147.5	gelatin	10310	70	12850	11650
41229-12075	155-160	157.5	liquified gelatin	11310	60	13800	13000
41229-12173	165-170	167.5	gelatin	11410	70	13800	13100
41229-N.D.	185-190	187.5	gelatin	11550	70	13900	13150
41229-12176	195-200	197.5	gelatin	12110	90	15350	13650
41229-12177	210-215	212.5	liquified gelatin	14400	80	17850	16650
41229-12073*	220-225	222.5	gelatin	12570	80	15550	14150
41229-12076	235-240	237.5	gelatin	14700	90	18250	17050
41229-12137	260	260	humic acid	15290	90	18950	17650
41229-12179*	270-275	272.5	gelatin	13940	100	17350	16150
41229-12180	295-300	297.5	gelatin	16240	100	20050	18650
41229-12181	300-305	302.5	gelatin	16620	110	20550	19150
41229-12183	315-320	317.5	gelatin	16510	100	20350	18950
41229-12131	338-343	340.5	humic acid	16610	110	20550	19050
41229-12184	345-350	347.5	gelatin	16770	100	20650	19250

Supplementary Notes

Supplementary Note 1. DNA preservation and other taphonomic biases

Unlike other caves in North America, Hall's cave exhibits exceptional DNA preservation across the chronosequence. There are several lines of evidence for a uniform level of DNA preservation throughout the sequence that we investigated: (1) Both the bulk bone and the sediment samples all contained amplifiable endogenous DNA. (2) As opposed to what would be expected in a setting of increasing DNA damage through time, diversity is positively correlated with sample age for bulk bone assays (Figure 3c) and while diversity drops in the Younger Dryas for sediment samples, it increases in the Pleistocene (Figure 4c). (3) The presence of certain taxa throughout the sequence, such as *Sylvilagus* sp. and *Peromyscus* sp., serves as taphonomic controls, illustrating that there is no detectable decrease in DNA preservation over time. (4) There is no evidence of systematic changes in the relative DNA content when comparing qPCR results across assays and sample type. As depicted in Supplementary Figure 1, the 16S assay does appear to display higher relative DNA concentrations at depths 100 to 150 cm BD_T, however, if this pattern represented variable DNA preservation in the cave, we would expect the same pattern to be present in the 12S assay, which is not the case. Similarly, for the sediment samples (Supplementary Figure 2), sequence A displays a small increase in DNA concentration around 140 cm BD_T, but this pattern is not reflected in sequence B. Hence, we do not find evidence of any significant changes in DNA preservation over time that would affect the interpretation of our results.

The depositional processes of a faunal assemblage, such as Hall's Cave, must be understood to correctly infer paleoenvironmental changes from each accumulation. In Hall's Cave, the taphonomic processes were thoroughly investigated by Toomey[24], who found that the assemblage was mainly accumulated by predation or bone gathering. Raptors, in particular large owls, were found to be an important contributor to the assemblage, preying mainly on small mammals the size of rabbits and smaller. Furthermore, both small and large mammalian carnivores contributed to the assemblage by dragging prey into the cave. In our data, the presence of lagomorphs and rodents in all time periods indicate that raptor accumulation most likely occurred throughout the sequence, while the disappearance of most carnivores and large mammals at the beginning of the Younger Dryas period indicates that accumulation by large carnivores diminished over time. This change is unlikely to be a result of a change in the shape of the cave entrance as large animals such as *Odocoileus* and *Canis latrans* are found in Holocene layers (though infrequently). Furthermore, as carnivores represents the top of the food chain, their disappearance is unlikely to be a result of a change in deposition but must reflect a change in the surrounding ecosystem. The disappearance of large herbivores at the onset of the Younger Dryas, on the other hand, could be linked to the disappearance of carnivores. Still, the continued presence of raptors in the cave suggests that the loss of diversity in frogs and reptiles in the Holocene reflects a loss of these species in the area surrounding the cave. Compared to the faunal assemblage, the plant data are less affected by taphonomic processes. As noted by Toomey, hackberry seeds might have been washed into the cave from the surrounding land surface, but other dispersal routes, such as wind or transportation on birds, insects and other animals would also have contributed to the plant assemblage[25]. However, as the depositional processes for the plant and animal assemblages are very different, the two assemblages serve as important validations of each other.

In combination, data from pollen, plytholiths, sedimentary DNA, bulk bone DNA and morphological bone identifications details how the landscape changed around Hall's Cave from the Pleistocene to the Holocene. While certain species groups could be affected by a change in depositional processes, it is very unlikely that all species are.

Supplementary Note 2. Sampling and laboratory contamination

To monitor contamination from sampling through to sequencing, we included non-template controls at each stage of laboratory processing. For both bulk bone and sediment samples, at least one extraction blank and one PCR-blank was included in each batch of sample preparation. For bulk bone samples, a total of 10 blanks were sequenced. From these we identified *Homo sapiens*, *Sus scrofa* and *Canis* sp., which were marked as laboratory or field contaminants and excluded from downstream data analysis. Moreover, although not identified in the controls, we identified a number of other common contaminants in the data. These include: *Gallus gallus*, *Bos* sp., *Ovis* sp., *Capra* sp. and *Felis* sp. Although some of these identifications could be from endogenous DNA (e.g. *Capra*), they are widely reported as common laboratory contaminants in ancient DNA studies[14,26,27] and were therefore marked as possible contamination and excluded from downstream analyses. Furthermore, the identification of turkey (*Meleagris gallopavo*) and caballine horse (*Equus caballus/lambei/scotti*) could represent contamination because these species are often associated with human everyday life (although rarely reported as contaminants in the literature). Hence, to confirm that these identifications represented endogenous DNA, we re-extracted and amplified the samples in which they were detected. Reassuringly, all re-processed samples confirmed their presence. Lastly, as we have marked *Canis* sp. as contamination in our data, the identification of *Canis latrans* could pose a problem. Hence, we confirmed that sequences assigned to *C. latrans*, were in fact distinguishable from the contaminant sequences from *Canis* (most likely *Canis lupus familiaris*) that we detected. In cases where reads could not be distinguished between different *Canis* species, the reads were marked as possible contamination and excluded from downstream analysis.

From the bulk bone samples, we did not find any evidence of cross contamination between samples, however, in one sample (HCB23_B) we identified significant contamination from fauna of New Zealand, which could stem from samples processed in the same laboratory for a different project. To identify the source of this contamination, we re-extracted and amplified the bone powder from HCB23_A and HCB23_B, which confirmed the presence of significant DNA contamination in the bone powder of both of these samples. Next, we returned to the original bulk bone samples, sampled and processed another 2x50 bones each. These samples contained only species from Texas and were comparable to other samples from the surrounding layers. Hence, we conclude that the bulk bone powder from the two subsamples of HCB23, was contaminated during the bone grinding stage. Most likely, this contamination stems from the reuse of a grinding pot that had not been cleaned properly. To confirm that this contamination event was a single incidence, we processed two 'grinding blanks' in which 15 mL of ultrapure water was run in the ball mill under the same configuration as the bulk bone samples. After grinding, the water sample was concentrated to 500 µL in an Amicon®Ultra-4 Centrifugal Filter (Millipore) and processed as a bulk bone sample. From these samples, we only identified background contamination from *Homo sapiens*.

This study includes samples that were excavated following strict ancient DNA guidelines (excavated for this study), and samples that were excavated solely for morphological analyses where no measures were taken to limit DNA contamination (Toomey et al. 1993). Hence, our dataset offers a unique opportunity to compare the level of contamination between the two. Not surprisingly, we found that the fossils excavated by Toomey exhibited a higher number of contaminant taxa (mean: 3.0 contaminant taxa per sample) than those excavated for this study (mean: 1.3 contaminant taxa per sample; Supplementary Figure 3). The level of contaminant taxa for the data excavated for this study is comparable to that of the controls (mean: 1.3 contaminant taxa per sample) and the sediment samples analysed with vertebrate assays (mean: 0.8 contaminant taxa per sample), indicating that the only source of contamination for these samples is background laboratory contamination. The higher level of contamination for the samples excavated by Toomey, on the other hand, suggests that excavations that do not follow ancient DNA guidelines have significantly increased risk of contamination. For example, the two contaminants *Felis* sp. and *Gallus gallus* are only detected in the Toomey samples. Fortunately, this level of contamination does not affect the interpretation of the data, as the common contaminants are easily distinguishable from the local fauna in Texas. This does, however, highlight the need for secondary authentication in metabarcoding studies where

domesticates are detected – in particular, in ancient DNA studies on samples excavated for other purposes than ancient DNA.

For the plant data, a total of three extraction blanks were sequenced. From these we identified three contaminant reads (taxa: *Cicer* sp., Brassicaceae, and 'no blast hit'), which were abundant in the extraction blanks, and present in low concentrations in some of the test samples—these reads were excluded from downstream analysis. Furthermore, in sediment extraction blank 3 we identify 9 copies of the *Celtis* read that is present in high numbers in all of the test samples. This most likely represents cross contamination during the PCR-reaction. However, this low level of cross contamination is unlikely to affect the sediment samples, as they all exhibit high concentrations of endogenous DNA. Extraction and PCR blanks are likely to overestimate cross contamination levels as low-level background contamination is more likely to amplify in samples with no endogenous DNA.

Supplementary References

1. Fick, S. E. & Hijmans, R. J. WorldClim 2: new 1-km spatial resolution climate surfaces for global land areas. *Int. J. Climatol.* **37**, 4302–4315 (2017).
2. Coolen, M. J. L. & Overmann, J. Analysis of Subfossil Molecular Remains of Purple Sulfur Bacteria in a Lake Sediment. *Appl. Environ. Microbiol.* **64**, 4513–4521 (1998).
3. Pollard, H. G., Colbourne, J. K. & Keller, W. (Bill). Reconstruction of Centuries-old Daphnia Communities in a Lake Recovering from Acidification and Metal Contamination. *Ambio* **32**, 214–218 (2003).
4. Poulain, A. J. *et al.* Microbial DNA records historical delivery of anthropogenic mercury. *ISME J.* **9**, 2541–2550 (2015).
5. Wooller, M. J., Gaglioti, B., Fulton, T. L. & Lopez, A. Post-glacial dispersal patterns of Northern pike inferred from an 8800 year old pike (Esox cf . lucius) skull from interior Alaska. *Quat. Sci. Rev.* **120**, 118–125 (2015).
6. Pedersen, M. W. *et al.* Postglacial viability and colonization in North America's ice-free corridor. *Nature* **537**, 45–49 (2016).
7. Pal, S., Gregory-Eaves, I. & Pick, F. R. Temporal trends in cyanobacteria revealed through DNA and pigment analyses of temperate lake sediment cores. *J. Paleolimnol.* **54**, 87–101 (2015).
8. Stager, J. C., Sporn, L. A., Johnson, M. & Regalado, S. Of Paleo-Genes and Perch: What if an "Alien" Is Actually a Native? *PLoS One* 1–9 (2015). doi:10.1371/journal.pone.0119071
9. Anderson-Carpenter, L. L. *et al.* Ancient DNA from lake sediments: bridging the gap between paleoecology and genetics. *BMC Evol. Biol.* **11**, 30 (2011).
10. Hofreiter, M., Mead, J. I., Martin, P. & Poinar, H. N. Molecular caving. *Curr. Biol.* **13**, 693–695 (2003).
11. Epp, L. S. *et al.* Lake sediment multi-taxon DNA from North Greenland records early post-glacial appearance of vascular plants and accurately tracks environmental changes. *Quat. Sci. Rev.* **117**, 152–163 (2015).
12. Hebsgaard, M. B. *et al.* 'The Farm Beneath the Sand'- an archaeological case study on ancient 'dirt' DNA. *Antiquity* **83**, 430–444 (2009).
13. Pedersen, M. W. *et al.* A comparative study of ancient environmental DNA to pollen and macrofossils from lake sediments reveals taxonomic overlap and additional plant taxa. *Quat. Sci. Rev.* **75**, 161–168 (2013).
14. Seersholm, F. V. *et al.* DNA evidence of bowhead whale exploitation by Greenlandic Paleo-Inuit 4000 years ago. *Nat. Commun.* (2016). doi:10.1038/ncomms13389
15. Porter, T. M. *et al.* Amplicon pyrosequencing late Pleistocene permafrost : the removal of putative contaminant sequences and small-scale reproducibility. *Mol. Ecol. Resour.* **13**, 798–810 (2013).
16. Wang, Y. *et al.* The southern coastal Beringian land bridge: cryptic refugium or pseudorefugium for woody plants during the Last Glacial Maximum? *J. Biogeogr.* **44**, 1559–1571 (2017).
17. Graham, R. W. *et al.* Timing and causes of mid-Holocene mammoth extinction on St. Paul Island, Alaska. *Proc. Natl. Acad. Sci.* **113**, 201604903 (2016).
18. Haile, J. *et al.* Ancient DNA reveals late survival of mammoth and horse in interior Alaska. *Proc. Natl. Acad. Sci. U. S. A.* **106**, 22352–22357 (2009).
19. Willerslev, E. *et al.* Fifty thousand years of Arctic vegetation and megafaunal diet. *Nature* **506**, 47–51 (2014).
20. Riaz, T. *et al.* ecoPrimers: inference of new DNA barcode markers from whole genome sequence analysis. *Nucleic Acids Res.* **39**, e145 (2011).
21. Taylor, P. G. Reproducibility of Ancient DNA Sequences from Extinct Pleistocene Fauna. *Mol. Biol. Evol* **13**, 283–285 (1996).
22. Poinar, H. N. *et al.* Molecular Coproscopy: Dung and Diet of the Extinct Ground Sloth. *Science (80-.).* **281**, 402–406 (1998).

23. Taberlet, P. *et al.* Power and limitations of the chloroplast trnL (UAA) intron for plant DNA barcoding. *Nucleic Acids Res.* **35**, (2007).
24. Toomey, R. S., Blum, M. D. & Valastro, S. Late Quaternary climates and environments of the Edwards Plateau, Texas. *Glob. Planet. Change* **7**, 299–320 (1993).
25. Hunt, C. O. & Fiacconi, M. Pollen taphonomy of cave sediments: What does the pollen record in caves tell us about external environments and how do we assess its reliability? *Quat. Int.* **485**, 68–75 (2017).
26. Seersholm, F. V. *et al.* Subsistence practices, past biodiversity, and anthropogenic impacts revealed by New Zealand-wide ancient DNA survey. *Proc. Natl. Acad. Sci.* 201803573 (2018). doi:10.1073/pnas.1803573115
27. Leonard, J. A. *et al.* Animal DNA in PCR reagents plagues ancient DNA research. *J. Archaeol. Sci.* **34**, 1361–1366 (2007).
