# Supplementary material for: Rapid range shifts and megafaunal extinctions associated with late Pleistocene climate change
Source: Nat Commun. 2020 Jun 2;11:2770. doi: 10.1038/s41467-020-16502-3 (PMC7265304; doi:10.1038/s41467-020-16502-3)
Supplement: Supplementary file 3 — Reporting Summary [file 41467_2020_16502_MOESM3_ESM.pdf]

nature research

Corresponding author(s): Frederik Seersholm

Last updated by author(s): 2020-4-16

Reporting Summary

Nature Research wishes to improve the reproducibility of the work that we publish. This form provides structure for consistency and transparency in reporting. For further information on Nature Research policies, see Authors & Referees and the Editorial Policy Checklist .

Statistics

For all statistical analyses, confirm that the following items are present in the figure legend, table legend, main text, or Methods section.

n/a	Confirmed	
☐	☒	The exact sample size (*n*) for each experimental group/condition, given as a discrete number and unit of measurement
☐	☒	A statement on whether measurements were taken from distinct samples or whether the same sample was measured repeatedly
☐	☒	The statistical test(s) used AND whether they are one- or two-sided<br>*Only common tests should be described solely by name; describe more complex techniques in the Methods section.*
☒	☐	A description of all covariates tested
☒	☐	A description of any assumptions or corrections, such as tests of normality and adjustment for multiple comparisons
☐	☒	A full description of the statistical parameters including central tendency (e.g. means) or other basic estimates (e.g. regression coefficient) AND variation (e.g. standard deviation) or associated estimates of uncertainty (e.g. confidence intervals)
☒	☐	For null hypothesis testing, the test statistic (e.g. *F*, *t*, *r*) with confidence intervals, effect sizes, degrees of freedom and *P* value noted<br>*Give P values as exact values whenever suitable.*
☒	☐	For Bayesian analysis, information on the choice of priors and Markov chain Monte Carlo settings
☒	☐	For hierarchical and complex designs, identification of the appropriate level for tests and full reporting of outcomes
☒	☐	Estimates of effect sizes (e.g. Cohen's *d*, Pearson's *r*), indicating how they were calculated

Our web collection on statistics for biologists contains articles on many of the points above.

Software and code

Policy information about availability of computer code

Data collection	Illumina MiSeq system
Data analysis	obitools v. 1.2.11<br>sumaclust v. 1.0.31<br>vsearch v. 2.6.0<br>blast+ v. 2.7.1<br>blast_getLCA v.1.0<br>vegan v2.4-5<br>ggplot2 v3.1.0<br>reshape2 v1.4.3<br>gridExtra v2.3<br>rworldmap v1.3-6

For manuscripts utilizing custom algorithms or software that are central to the research but not yet described in published literature, software must be made available to editors/reviewers. We strongly encourage code deposition in a community repository (e.g. GitHub). See the Nature Research guidelines for submitting code & software for further information.

Data

Policy information about availability of data

All manuscripts must include a data availability statement. This statement should provide the following information, where applicable:

- Accession codes, unique identifiers, or web links for publicly available datasets
- A list of figures that have associated raw data
- A description of any restrictions on data availability

Fastq files for all DNA sequencing data reported in this paper was deposited in the European Nucleotide Archive under study accession number

"PRJEB37627 [http://www.ebi.ac.uk/ena/data/view/PRJEB37627]"
The source data underlying Figs 3c, 4c, and Supplementary Figs 6, 9 and 17 are provided as a Source Data file. All databases used in this study are publicly available online:
"NCBI nt database" [ftp://ftp.ncbi.nlm.nih.gov/blast/db/nt*gz],
"UCSD Plants database" [https://plants.sc.egov.usda.gov/adv_search.html],
"The IUCN Red List of Threatened Species" [https://www.iucnredlist.org],
"WorldClim version 2" [https://biogeo.ucdavis.edu/data/worldclim/v2.1/base/wc2.1_10m_bio.zip]

Field-specific reporting

Please select the one below that is the best fit for your research. If you are not sure, read the appropriate sections before making your selection.

☐ Life sciences ☐ Behavioural & social sciences ☒ Ecological, evolutionary & environmental sciences

For a reference copy of the document with all sections, see nature.com/documents/nr-reporting-summary-flat.pdf

Ecological, evolutionary & environmental sciences study design

All studies must disclose on these points even when the disclosure is negative.

Study description	The study is a comparison of ancient DNA metabarcoding data based on bulk bone from a sedimentary profile in Hall's cave, Texas, spanning around 12,000 years.
Research sample	This research is based on data from 30 newly excavated bulk bone samples of approximately 100 bones each from throughout the excavation profile at Hall's Cave. Furthermore, 32 sediment samples was taken from three time periods (Bølling-Allerød, Younger Dryas and Early Holocene) to characterise vegetational changes during the Younger Dryas. Lastly, data from six bulk bone samples made from large fragmentary fossils excavated by Toomey in 1993, has been included in the study. However, as these samples are not readily comparable to the recently excavated samples, data form these samples have been excluded in quantitative comparisons (e.g. Fig. 3b and 3c.).
Sampling strategy	Bulk bone samples were collected by excavating sediment in approximately 3-cm levels, which were subsequently dry-sieved through 3mm and 1.5 mm sieves to obtain bulk bone material. In total, 110 levels were excavated for bulk bone material, yielding from 20 to over 300 bone fragments each. After excavation and sieving, levels yielding fewer than 100 bones were merged, to ensure that all samples could be subsampled to 100 bone fragments. This sample size was chosen based on unpublished comparisons of differently sized bulk bone samples.
Data collection	Data was collected on the Illumina MiSeq system.
Timing and spatial scale	Samples were collected from a single site. Samples cover a period of approximately 12,000 years, and was deposited between 20,000 and 8,000 years ago.
Data exclusions	Six bulk bone samples made from large fragmentary fossils excavated by Toomey in 1993, has been included in the study. However, as these samples are not readily comparable to the recently excavated samples, data from these samples have been excluded in quantitative comparisons (e.g. Fig. 3b and 3c.).
Reproducibility	Data collection was carried out in duplicates (e.g. for each layer two subsamples of 50 bone fragments each were analysed separately). The large overlap in species composition between duplicates confirms the reproducibility of the analyses.
Randomization	Samples were grouped into four distinct climate intervals for comparative analyses: (1) Last Glacial Maximum (ca. 20 to 14.7 ka CAL BP); (2) Bølling-Allerød (14.7 to 12.6 ka CAL BP); (3) Younger Dryas (12.6 to 11.7 ka CAL BP); and (4) Early Holocene (11.7 to 8 ka CAL BP)
Blinding	Not relevant as data acquisition was carried out by an automated DNA sequencing system.

Did the study involve field work? ☒ Yes ☐ No

Field work, collection and transport

Field conditions	Field work was carried out inside Hall's Cave
Location	Hall's Cave, Kerr county, Texas, U.S.A
Access and import/export	The excavations were on private land, therefore no state or federal permits were needed, however, accident waivers were signed with the Hall's family. Samples were imported to Australia on import permit IP15012450 issued by the Department of Agriculture, Fisheries & Forestry on September 15th 2015.
Disturbance	We removed about 1 m square to a depth of 3 meters, a volume representing approximately 0.1% of the remaining cave fill.

Reporting for specific materials, systems and methods

We require information from authors about some types of materials, experimental systems and methods used in many studies. Here, indicate whether each material, system or method listed is relevant to your study. If you are not sure if a list item applies to your research, read the appropriate section before selecting a response.

Materials & experimental systems

n/a	Involved in the study
☒	☐ Antibodies
☒	☐ Eukaryotic cell lines
☐	☒ Palaeontology
☒	☐ Animals and other organisms
☒	☐ Human research participants
☒	☐ Clinical data

Methods

n/a	Involved in the study
☒	☐ ChIP-seq
☒	☐ Flow cytometry
☒	☐ MRI-based neuroimaging

Palaeontology

Specimen provenance	All samples were obtained from a 4-meter thick section of the northern face of composite pit 1d/e in Hall's Cave in August and September 2016. The excavations were on private land, therefore no state or federal permits were needed, however, accident waivers were signed with the Hall's family. Samples were imported to Australia on import permit IP15012450 issued by the Department of Agriculture, Fisheries & Forestry on September 15th 2015.
Specimen deposition	The specimens have been deposited at the TRACE aDNA facility at Curtin University, Western Australia. Specimens are available for researchers upon request.
Dating methods	No new dates are provided

☒ Tick this box to confirm that the raw and calibrated dates are available in the paper or in Supplementary Information.
