# Supplementary material for: Rapid range shifts and megafaunal extinctions associated with late Pleistocene climate change
Source: Nat Commun. 2020 Jun 2;11:2770. doi: 10.1038/s41467-020-16502-3 (PMC7265304; doi:10.1038/s41467-020-16502-3)
Supplement: Supplementary file 4 — Description of Additional Supplementary Files [file 41467_2020_16502_MOESM4_ESM.pdf]

Description of Additional Supplementary Files

File name: Supplementary Data 1
File name: ASV table for vertebrate data, including information on taxonomic assignments for each read.

File name: Supplementary Data 2
File name: ASV table for plant data, including information on taxonomic assignments for each read.

File name: Supplementary Data 3
File name: Taxa identified, vertebrates. Counts indicate number of replicates in which each taxon was identified. *Common contaminants.

File name: Supplementary Data 4
File name: Taxa identified, plants. Counts indicate presence (1) or absence (0) of a taxon.
