# Supplementary material for: Rapid range shifts and megafaunal extinctions associated with late Pleistocene climate change
Source: Nat Commun. 2020 Jun 2;11:2770. doi: 10.1038/s41467-020-16502-3 (PMC7265304; doi:10.1038/s41467-020-16502-3)
Supplement: Supplementary file 2 — Peer Review File [file 41467_2020_16502_MOESM2_ESM.pdf]

Reviewers' comments:

Reviewer #1 (Remarks to the Author):

Review of Seersholm et al.: Rapid range shifts and megafaunal extinctions driven by late Pleistocene climate change

This is quite a data-rich manuscript that tracks the evolution of fauna and flora from a well-studied cave in central Texas that spans the time from peak cold of the last glaciation through the oscillating transition into the warmth of the early Holocene. Previous research at the cave had documented major changes in large vertebrate fauna, but not flora, and importantly provided a firm 14C chronology that showed a relatively rapid sedimentation rate providing high resolution for at least the key time period from 20 ka well into the Holocene. What is new here, is the novel application of aDNA tools using both bulk un-diagnostic bone material that preserves taxonic diagnostic aDNA, and sedaDNA from which plant taxa were identified. The local sourcing of sedaDNA provides a more authentic representation of local vegetation compared to pollen analysis that is in part compromised by long-distance transport of some pollen types. And the bulk bone aDNA document several key taxa not found by standard paleontological techniques.

A major goal of this study centers on the long-standing debate on the roles of climate vs human agency in the extinction of the North American megafauna and the large changes in extant floral and faunal elements across the US. A debate now over a century in duration. This study shows that both flora and fauna underwent dramatic changes in the transition from LGM to Holocene, but during the warmth of the early Holocene floral elements are re-established, whereas large vertebrates do not. The only change that might impact faunal recovery is the appearance of Homo sapiens for the first time between LGM and Holocene. The authors conservatively argue that there may have been a 1-2 climate/human agency punch that drove the megafauna to extinction. I can accept that, although the fact that floral elements appear to have effectively migrated with climate space change during the YD, and reappeared with the climate warming of the early Holocene, then why didn't the megafauna do the same thing, as their movements are far more efficient than plant dispersal.

From a technical standpoint, the ms demonstrates the utility of bulk-bone aDNA, which identified 7 taxa not recognized by paleontologists, although also missed quite a few taxa identified by fossil remains. In that regard, I urge Nat Comm to include Supplementary Figure 4 in the published ms, as it provides the most quantitative assessment of these two complimentary tools, and confirms the utility for both.

One of the most fascinating conclusions is that soil depth (burrowing animals) changed dramatically from a meter or more during LGM to a few cm by the early Holocene. This has impacts on both flora and fauna independent of temperatures, and suggests a first-order trend to aridity and aeolian deflation, with a result that transforms the flora and burrowing fauna. Although this has been argued for before based on Sr isoptopes, to see the impact on burrowing animals is quite exceptional, and provide greater detail as well as demonstrating the biotic consequnces.

General issues
Abstract Most of the abstract succinctly describe key elements of this study, except for the final sentence: "Our findings demonstrate that biotic shifts occurred rapidly and closely tracked fluctuating temperatures over the Pleistocene/Holocene boundary in central North America" Rather, the preceding two sentences of the abstract show that only the flora follow the standard deglacial climate zonations, whereas the large vertebrates do not, as they fail to recover in the early Holocene. I think readers will expect the human role in this study to be included in the abstract, which based on the Conclusions paragraph of the ms should say something like "climate change alone cannot explain the disappearance of the megafauna at the end of the Pleistocene" or some such text.

Dating Because geochronology is such an important element in a study like this, I think that the supplemental data must include the primary 14C data that all Quaternary studies are now expected to provide, including Lab ID, Sample Material, Conventional 14C age and uncertainty, and calibrated age and uncertainty. There are numerous tools to transform a series of dates and specific depths into a down-core age model. The authors chose a combinations of variables that gives very high weighting to each 14C age, resulting an age model with abrupt, high-magnitude shifts in sedimentation rate (Sup Fig 10). This may well be correct, but it would be useful to show an alternative version where the smoothing is much greater, and then indicate at what depth the breaks between the four chronostratigraphic occur (LGM, B/O, YD, Holocene). Because the ms does not argue for any within-unit patterns, the only key variable of interest is the depth of thise three chronostratigraphic breaks (14.7 ka, 12.9 ka, and 11.7 ka. It may well be that those depths are relatively insensitive to the variable means to reconstruct an age-depth model. It appears that only the 14.7 ka break might be sensitive.
And because there is an age model, I would like to see Figure 1 show the Halls Cave column presented in calibrated age rather than depth, especially since the GISP paleotemperature record is presented against time (and those ages are NOT calibrated ages (they are not based on 14C) so should be just labled years BP, or more correctly for the ice core Y2k (years before 2000 CE rather than 1950 CE).

Subdivision of the Late Quaternary The authors use the Greenland Ice Core data to define the subdivisions of the Late Quaternary. These subdivisions are derived from northern North Atlantic signals present in the ice core and across NW Europe, where they are clearly represented in proxy records in terms of both temperature and hydrology. Exactly how these units are represented in central Texas is MUCH less clear, and I would caution the authors from directly transferring European climate terms to Texas. For example, the authors state that the YD was a "return to cold and dry glacial conditions", whereas data from parts of Texas, New Mexico and Arizona suggests that the Younger Dryas is represented by proxies indicative of cooler but wetter conditions ( ). In addition Meltzer and Holliday (2010; World Prehist (2010) https://doi.org/10.1007/s10963-009-9032-4) argue that while the Greenland record records abrupt, dramatic hydro-temperature changes at the onset of the YD, climate changes on the Great Plains and in the Rocky Mountains, were less extreme A similar argument is developed by Holliday et al. (2011; doi:10.1016/j.quaint.2011.03.047).
Specific Points
1) cal BP is usually written in lower case "cal" not CAL
2) line 70: "temperatures rapidly returned to glacial conditions in the Northern Hemisphere12." This reference is for Greenland only, and as pointed out above, the YD climate of the southern great plains is not considered to be the same as the LGM. Some recognition needs to be given that the NW North Atlantic record for the LGM-Holocene transition is not applicable in an absolute sense for the Northern Hemisphere as a whole.
3) line 92 "approximately 100 bones each" change to "approximately 100 small non-diagnostic bones each"
4) line 105 Omit "For comparative analysis" at start of sentence as appears later in same
5) line 133 Omit "only" end of sentence
6) line 136 "other sources" change to "source other"
7) Line 139 "OTU" Spell out first time
8) Line 167 "separated the three time periods" There are 4 time periods
9) Line 168 "limited resolution" Do you mean "limited abundance"?
10) line 235 Delete "has"
11) line 236 The authors soil erosion evidence is certainly NOT identical to that of Cooke et al. (2003) which is based on Hall's Cave Sr isotope ratios and concludes "... a constant minimum rate of 11 cm/k.y.; this continuous phase of erosion ended ca. 5 ka". I think "consistent with" is far more accurate.
12) Line 268 "increased abundance of oak DNA" Abundance is usually difficult to determine for DNA analyses because the PCR amplifies variably. And the correct Supp Fig, is Fig. 9 not Fig, 8. Maybe they mean oak appears more frequently in YD levels than elsewhere?
13) lines 281-282 Ref 34 does not seem relevant to this statement

14) lines 297-298 "....evidence supporting dramatic climatic and ecological changes in central Texas after the end of the Last Glacial Maximum" I dont think you can say this. A problem here is that many of your vertebrate taxa go extinct, so that using ecological change as a straight forward proxy for climate is compromised. You might say "evidence supporting dramatic ecological change in central Texas between the LGM and Holocene, some of which is likely due to climate change, but in the same time period humans appear for the first time...."

15) line 306 "desertification" is defined in way that it cannot be used here. Find another word or words. And it is not clear that colder, drier conditions (which I think is what you mean) "caused" plant and animal diversity to decline. In other parts of Texas and adjacent states "black mats" that formed during YD suggest increased effective moisture (e.g. Harris Park (2017; https://doi.org/10.1016/j.yqres.2015.11.005 and papers by Vance Haynes and colleagues).

16) line 310 " decimated by megafaunal extinctions, did not recover" Statement OK, but we need you to define the time that this decimation occurred. This seems to me to be a very key point of this paper. Can you define a time within a specified uncertainty, when the aDNA indicates extinction had occurred?

17) line 316 "The fact that plant diversity recovered after the Younger Dryas but large vertebrates did not suggests that factors other...." I think this needs to be added to make it clear what you are basing you argument here on...

18) line 325 "...suggest that human hunting of large mammals, likely together with climate change...." I think the fact that you argue that the plants were able to migrate with climate change, and so recover in the Holocene leaves the reader to imagine that large grazing mammals could rather easily migrate with their preferred plant diet sources, hence climate change is not the "obvious" explanation for their extinction. I find the aDNA evidence to make a rather compelling case for only humans as the extinction source, but recognize that climate cannot be totally ruled out.

19) line 334 "a" not "an"

20) line 345 "Pollen records from other parts of central Texas suggest that the region was covered in conifer forests during full glacial times" Since you earlier point out that there is NO conifer aDNA and because conifer pollen is exceptionally efficiently dispersed by wind, I expected you to conclude the obvious: " Although pollen records from other parts of central Texas suggest that the region was covered in conifer forests during full glacial times, the lack of conifer aDNA in any of our samples suggests that conifer pollen was aeolian-derived from distand sources". And then modify subsequent text. This seems a very important contribution of this study.

21) lines 354-55 I would like to see this sentence expanded..what are the climate implications of this important conclusion

OVERALL

I strongly support publication of this ms in Nat Comms, with the modest suggested changes outlined above. It contains an exceptional mass of aDNA data, maybe unique in the combination of both plant and vertebrate reconstructions across one of the most climatically variable time periods in the Quaternary, and addresses one of the most compelling questions in Quaternary research: what was the impact of continent colonization by modern humans where humans had not previously been present.

Gifford Miller, Univ Colorado

Reviewer #2 (Remarks to the Author):

This is an important paper, with a richly detailed record of community diversity and turnover during a major period of environmental change (the last glacial maximum to early Holocene) and for a region with few paleoecological records. It is also a major demonstration of the potential for bulk-bone and sedimentary ancient DNA techniques for recovering ~species-level genetic signatures of many taxa. My comments below are generally minor to moderate, and are divided into three sections: 1) high-level comments, 2) line-by-line comments (* indicates more substantive comments), and 3) comments on the supplementary info.
MODERATE
1. Diversity and uneven sampling. Alpha richness efforts (such as the ones shown in Figs. 3, 4) are known to be highly sensitive to sampling effort: the more one looks, the more species one will find. The sampling effort per depth interval appears to be fairly constant (e.g. 2 x 50 bulk-bone replicates per sample) but the number of sampled layers differs among the four time periods (17 LGM, 6 B-A, 4 YD, 9 EH). So, for example, could the lower reported diversity during the YD simply be because sampling effort is half as large as for the Early Holocene? There are well-established approaches for correcting for sampling effort, via rarefaction analyses. The paper ideally would pursue a rarefaction analysis to correct for sampling effort and at minimum should note the

2. I recommend a figure (probably in supplementary information) that repeats the vertebrate diversity figure for different taxonomic subgroups, e.g. for birds, small mammals, megaherbivores, and reptiles and amphibians. Supp Table 7 suggests that the loss of bird diversity was particularly acute, but this is not apparent in Fig. 3.

3. The paper tends to be somewhat narrowly focused on its own data and a bit decontextualized from the prior literature on deglacial vegetation and climate dynamics in the central and eastern US. For example, the finding that plant diversity was largely I've suggested below a number of relevant papers.

4. The review of vegetation history in the paper would benefit from a closer comparison of the plant sedaDNA data to the pollen and phytolith data from Cordova and Johnson 2019, both in terms of representation of individual taxa and overall ecosystem interpretation. The current comparison is fairly cursory. Suggest merging L346-355 from Methods/Site Description into the main text.

5. Methods: Need to add a section describing the NMDS ordination. What is a taxonomy-independent approach? Why do the results in Fig. 3b differ from the top two rows in Supp Fig. 5a? Why do the results in Fig. 4b differ from the bottom two rows in Supp Fig 5a?

6. Paper would be strengthened by a summary figure (perhaps in Supplementary Info) that summarized the interpreted environmental history at Halls Cave. i.e. with an x-axis for time and then different horizontal plots indicating e.g. trends in temperature, soil depth, ecosystem type, and diversity.
LINE-BY-LINE COMMENTS (* indicates more substantive points)
L40-42: This is an oversell – there are *many* high-resolution fossil pollen records spanning the Younger Dryas, in eastern North America, Europe, and elsewhere, that are well able to characterize the pace and scale of biotic turnover. See e.g. Gonzales and Grimm et al. 2009 QR; Williams et al. 2002 Geology

L64: The dates of Widga et al. 2017 Boreas suggest a slightly later extinction date for mastodons, ca. 12.7 to 12.5 ka.
L72-74: Delete this sentence on ‘difficult to describe’ climatic events YD & BA or extend to review what is known. There is a massive paleoclimatic literature on both events, with respect to both proxy data and paleoclimatic models; these are very well-studied & understood climatic events. See e.g. Peter Clark et al. 2012 PNAS; Shakun & Carlson 2010 QSR; Shakun et al. 2012 Nature
L78: upper-case ‘central’ or lower-case ‘North’ for consistency
L82: Insert ‘cave’ before ‘sites’ – many lakes have very good YD records. For example, the ca. 20cm interval representing the YD at Hall’s Cave might be 1-2 m of sediment in many eastern US kettle lakes.
L86 delete hyphen from ‘turn-over’
L104: insert ‘while’ before ‘acknowledging’
L107-108: insert ‘in the Northern Hemisphere’ at the end of item 3; YD cooling is mainly a NH phenomenon
L115: which targeted time interval? Four are mentioned in the previous section.
L115: insert ‘species’ after ‘vertebrates’
L133: ‘only’ shows up twice here
L137: insert comma after ‘hair’
L141: delete comma after ‘Celtis’
L143: it->they
L145: significantly -> substantively (‘significantly’ implies a statistical test)
L148: form->from
*L148-151: Further sharpen by noting that Pinus is particularly known for being overrepresented in fossil pollen records, due to its high pollen productivity and dispersability. See e.g. Dawson et al. 2016 QSR. So this particular data-data mismatch is interesting but not particularly surprising.
L148-151: Also worth noting that in Cordova & Johnson 2019, Celtis is a rare type, whereas it is the most common type in the sedaDNA record.
Figure 1: The Great Lakes and other pro-glacial lakes are incorrectly color-coded, so that they appear to be part of the 9ka Laurentide ice sheet.
L154: CAL -> cal. Also fix in Figure 1 legend, axis titles, and elsewhere.
L155: Clarify that right panel shows air temperature *over Greenland* and is based on d18Oice.
L163: was consistent ->persisted
L164: what kind of ‘public reference databases’? Genomic?
L165: similar to what? Comparison is unclear.
L168: what kind of resolution? Taxonomic?
*L170 & Figure 3: If the extinct taxa were not included in the analysis, would there still be a net decline in diversity? Also, do the small and large mammals show different diversity trends? Suggest creating a few additional variants on panel Fig. 3c, either in main text or Supp Info.
L169-172: All these sentences are written in first person, which adds words and shifts the subject/action emphasis away from the actual findings to the scientists. For example, the second sentence could easily be rewritten as ‘For vertebrates, diversity significantly declined from the Pleistocene…’ This slight rewrite is both more word-sparing and strengthens the subject/verb pair from ‘we found’ to ‘diversity declined’…
L175: ‘the climate warmed’ -> ‘temperatures rose’
Figure 2: What a beautiful data visualization!
L187: ‘the temperature’ -> ‘Greenland temperature’
L191: delete ‘cold climate’
*L197, L454 and elsewhere: Avoid use of ‘tolerance.’ Use of geographic data can show the current realized niche limit of a species, but cannot directly speak to the true fundamental climatic tolerances of a species. So e.g. L197 replace ‘biological tolerances’ with ‘current realized climatic niches’ and L454 replace ‘Climate tolerance limits’ with ‘Climatic realized niches’ or ‘Climatic realized niche limits’.
L198: ‘refine inference of the climate’ – awk
L204: a notable -> substantial
Fig. 3/L214-219: Clarify – is this figure showing data from both Sequences A & B?

Fig. 4/L243-246: Remind reader that it's the trnL data being analyzed here.
L248-265: Need to add citation here to Cordova & Johnson 2019 and integrate this interpretation with the available pollen and phytolith record from this site.
L249: Clarify: is this detection based on bulk bone aDNA or traditional bone identification?
L253: woody plants -> woody plant cover [or density]
L269: delete extra 'species'
L273: lower case for 'Flowers'
L275: insert 'in' after 'increase'
L276: larger than what? Unclear comparison.
L286: Suggest also citing here Gill et al. 2009 Science for another example of Pleistocene megaherbivore-vegetation interactions in eastern North America
L288: For the non-analog taxa, suggest citing Graham et al. 1996 Science and Williams and Jackson 2007 Frontiers in Ecology and Environment. Russ Graham has many papers about the disharmonious (no-analogue) mammal assemblages of eastern North America
L313: Delete 'forever' – that's a very long time…
L314-318: Might note here that, in contrast with vertebrates, there is only one documented extinction of a plant species in eastern North America during the last deglaciation. See Jackson & Weng 1999 PNAS.
L335: Could broaden the time scale range by changing to 'centuries to tens of millennia'
L344: grassy->grassland
L353: the combination of 'e.g.' and 'i.e.' here is hard to follow. Suggest rewriting as 'direct vegetation indicators such as phytoliths vs. indirect indicators such as faunal remains'
*L344-355: This whole section belongs in the main paper, so that the sedaDNA intepretations of vegetation history can be integrated with other lines of evidence. This section also omits mention/discussion of Cordova & Johnson 2019 (ref 24); a major omission.
*L355: I'd be more cautious about the apparent interpretation here of LGM grasslands. The floral sedaDNA evidence doesn't extend to the LGM (Fig. 4), while the mammalian species occurrences (Figs. 2-3) could be interpreted as grassland to open woodland. Also the 'by end of the Pleistocene' implies a unidirectional change from Pleistocene to Holocene vegetation, while authors' data suggest a more complex pattern, with a YD reversal along the way. Suggest extending this by a sentence or two to be more precise about the inferred vegetation changes.
L362: Should the units here be yr/cm, not just 1/cm?
*L375: In Sampling section, describe the depth datums – see queries in Supp Info.
SUPPLEMENTARY INFO
Figure S1: What defines the zero depth datum? Specify. Also specify the collection unit (pit 1d/E?).
Figure S3: Please add the common-name equivalents for all taxa listed in legend.
Figure S4: Remind reader what the crosses after taxon names indicate.
Figure S5: Remind reader what the panel titles mean. Also clarify here that this is a 'taxonomy-independent approach' and explain what this means. This is alluded to in main text (163-165) and needs more detail here.
Figure S10: What is the Toomey datum, where is it, and how is it defined or recognized?
Recommend reversing x-axis. I'm a strong proponent of time running forward from left to right; this follows the western convention of reading words from left to right. Reversing Fig 10 would also align it with the convention followed in Figs. 3-4.
Table S1: What are the bottom seven units and why do they lack metadata?
Table S6: What is meant by 'upgrade' and 'downgrade'? And, what does the animaldiversity.org URL support? The dietary status of each taxon?
Table S7: birds->bird

Reviewer #3 (Remarks to the Author):

The authors present a nice data set from a very special, long, continuous deposit.

Overall a larger diversity (number of species) is reported for the older (Pleistocene) sample, however that sample also contained a larger number of subsamples from a longer time period. Although I am inclined to believe the pattern reported, I am not convinced that the presented analyses show that. It could be more appropriate to use a diversity index, or subsampling to make the different samples (time periods) more comparable.

Also regarding the sampling (pg 19, sup) "the disappearance of most carnivores and large mammals at the beginning of the Younger Dryas period indicates that accumulation by large carnivores diminished over time" how do you think the change in the way bones accumulated in the cave through time impacts the pattern observed? Could a change in usage of the cave by large carnivores explain the disappearance of mammals too large for an owl to catch? (without requiring a change in the local population? ie. Fig 3, lines 238-241; lines 276-282; lines 317-318- this is really hard to differentiate from change in mode of bone deposition)

Lines 148-151 re Pinus- it sounds as if there is a pollen record including Pinus in the cave- is this correct? If so, one could expect to find DNA from Pinus (from the pollen) regardless of whether it grew adjacent to the cave or the pollen was dispersed potentially long distances. Please clarify.

The habitat modeling is a nice complement to the story.

Lines 416-419- put final concentrations of reagents (final molarity, number of units, etc) in total volume.

General remarks to reviewers

We would like to thank the reviewers for their time - their comments and suggestions have helped us improve the paper significantly. We have addressed all of the comments below and hope that the revised version of the manuscript is suitable for publication in Nature communications.

For easier reading, we have copied all reviewer comments into this letter (blue text color). In the main manuscript, we have highlighted changes in yellow.

Lastly, please note that we have updated the genetic reference database (blast database) to take into account any species that have been added to the database recently. This resulted in the identification of bog lemming (*Synaptomys cooperi*), northern crested caracara (*Caracara cheriway*) and true trush (*Turdus* sp.). All datasets and figures were updated to include these species.

Response to reviewer #1 comments

This is quite a data-rich manuscript that tracks the evolution of fauna and flora from a well-studied cave in central Texas that spans the time from peak cold of the last glaciation through the oscillating transition into the warmth of the early Holocene. Previous research at the cave had documented major changes in large vertebrate fauna, but not flora, and importantly provided a firm 14C chronology that showed a relatively rapid sedimentation rate providing high resolution for at least the key time period from 20 ka well into the Holocene. What is new here, is the novel application of aDNA tools using both bulk un-diagnostic bone material that preserves taxonic diagnostic aDNA, and sedaDNA from which plant taxa were identified. The local sourcing of sedaDNA provides a more authentic representation of local vegetation compared to pollen analysis that is in part compromised by long-distance transport of some pollen types. And the bulk bone aDNA document several key taxa not
found by standard paleontological techniques.

A major goal of this study centers on the long-standing debate on the roles of climate vs human agency in the extinction of the North American megafauna and the large changes in extant floral and faunal elements across the US. A debate now over a century in duration. This study shows that both flora and fauna underwent dramatic changes in the transition from LGM to Holocene, but during the warmth of the early Holocene floral elements are re-established, whereas large vertebrates do not. The only change that might impact faunal recovery is the appearance of Homo sapiens for the first time between LGM and Holocene. The authors conservatively argue that there may have been a 1-2 climate/human agency punch that drove the megafauna to extinction. I can accept that, although the fact that floral elements appear to have effectively migrated with climate space change during the YD, and reappeared with the climate warming of the early Holocene, then why didn't the megafauna do the
same thing, as their movements are far more efficient than plant dispersal.

From a technical standpoint, the ms demonstrates the utility of bulk-bone aDNA, which identified

7 taxa not recognized by paleontologists, although also missed quite a few taxa identified by fossil remains. In that regard, I urge Nat Comm to include Supplementary Figure 4 in the published ms, as it provides the most quantitative assessment of these two complimentary tools, and confirms the utility for both.
We agree that Supplementary Figure 4 is an important comparison to make from a technical point of view. However, as our main focus of this manuscript is the biological interpretations of the data, we prefer to keep this figure in the Supplementary Information. Furthermore, there is a great deal of information (genera names) in Supplementary Figure 4, that is already presented in Figure 2.

If the editor agrees with reviewer 1, we are of course more than willing to include the figure in the main text.

One of the most fascinating conclusions is that soil depth (burrowing animals) changed dramatically from a meter or more during LGM to a few cm by the early Holocene. This has impacts on both flora and fauna independent of temperatures, and suggests a first-order trend to aridity and aeolian deflation, with a result that transforms the flora and burrowing fauna. Although this has been argued for before based on Sr isoptopes, to see the impact on burrowing animals is quite exceptional, and provide greater detail as well as demonstrating the biotic consequnces.

General issues
Abstract Most of the abstract succinctly describe key elements of this study, except for the final sentence: "Our findings demonstrate that biotic shifts occurred rapidly and closely tracked fluctuating temperatures over the Pleistocene/Holocene boundary in central North America" Rather, the preceding two sentences of the abstract show that only the flora follow the standard deglacial climate zonations, whereas the large vertebrates do not, as they fail to recover in the early Holocene. I think readers will expect the human role in this study to be included in the abstract, which based on the Conclusions paragraph of the ms should say something like "climate change alone cannot explain the disappearance of the megafauna at the end of the Pleistocene" or some such text.
Corrected. The last sentence of the had been reworded to better address the human role:

> *Our findings demonstrate that while climate change affected the local ecosystem in Texas over the Pleistocene/Holocene boundary, it cannot alone explain the disappearance of the megafauna at the end of the Pleistocene.*

Dating Because geochronology is such an important element in a study like this, I think that the supplemental data must include the primary 14C data that all Quaternary studies are now expected to provide, including Lab ID, Sample Material, Conventional 14C age and uncertainty, and calibrated age and uncertainty.
Corrected. We have included Supplementary Table 12 with this information.

There are numerous tools to transform a series of dates and specific depths into a down-core age model. The authors chose a combinations of variables that gives very high weighting to each 14C age, resulting an age model with abrupt, high-magnitude shifts in sedimentation rate (Sup Fig 10). This may well be correct, but it would be useful to show an alternative version where the

smoothing is much greater, and then indicate at what depth the breaks between the four chronostratigraphic occur (LGM, B/O, YD, Holocene). Because the ms does not argue for any within-unit patterns, the only key variable of interest is the depth of thise three chronostratigraphic breaks (14.7 ka, 12.9 ka, and 11.7 ka. It may well be that those depths are relatively insensitive to the variable means to reconstruct an age-depth model. It appears that only the 14.7 ka break might be sensitive.

To test age-depth models with greater smoothing we analysed the dates using both *Clam* and *Bacon* in R. These two approaches gave very similar results. Since we have no reason to believe that there were frequent abrupt changes in sedimentation rates, we have decided to use the age-depth model produced by Clam for the analyses.

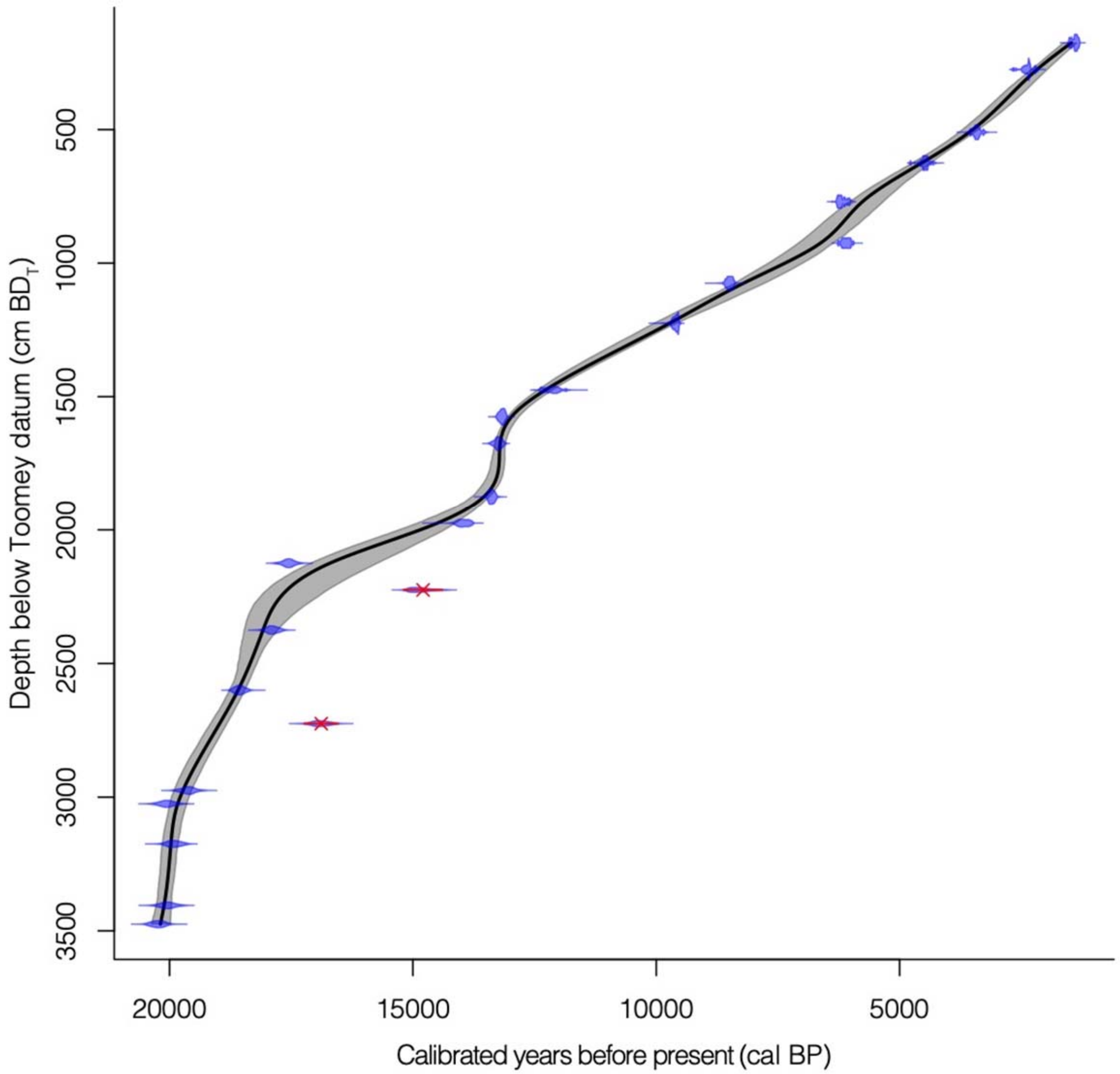

The use of a different age-depth model changes the breaks at 14.7, 12.9 and 11.7 slightly. However, as the sampling is sparse around 14.7 ka, no samples are grouped differently around this break. For the Younger Dryas interval, we have decided to use the base of the rancholabrean mat (151 cm) as the definition of the onset of the YD in our study - as in Bourne et al. (2016). Using the new age-depth model, the onset of the YD at 151 cm is dated to 12.6 ka (12,692- 12,396 ka) – slightly younger than the conventional dates for the onset of the YD (12.9 ka). This apparent time lag between the cooling over Greenland and a biological response in Texas was also reported for pollen records in Crystal Lake. However, the difference could also be explained by uncertainties in the age-depth model or contamination with humic acids from younger overlying layers. We have clarified this in the text:

> *Similar to many North American geoarchaeological sites, the profile in Hall's Cave have a clearly visible "rancholabrean mat" or organic rich layer associated with the Younger Dryas (see Supplementary Photo 1)[15]. We define the base of this layer (151 cm BD_T) as the onset of the YD. Based on the current age-depth model, 151 cm BD_T dates to 12.6 ka cal BP (12,692- 12,396 ka cal BP) - slightly younger than the conventional dates for the onset of the YD at 12.9 ka cal BP. This apparent time lag between the cooling over Greenland and a biological response in Texas was also reported for pollen records in Crystal Lake[28]. However, the difference could also be explained by uncertainties in the age-depth model or contamination with humic acids from younger overlying layers.*

And because there is an age model, I would like to see Figure 1 show the Halls Cave column presented in calibrated age rather than depth, especially since the GISP paleotemperature record is presented against time (and those ages are NOT calibrated ages (they are not based on 14C) so should be just labled years BP, or more correctly for the ice core Y2k (years before 2000 CE rather than 1950 CE).
As suggested we have updated Figure 1 to show samples plotted against calibrated age. Furthermore, we have changed the Y-axis label from "*Age (ka cal BP)"* to "*Age (ka)"* and specified the difference in dating methods between GISP2 and our data in the legend of Figure1:

> ***Figure 1. Sampling location and stratigraphy. a)*** *Location of Hall's Cave in North America, with the continental ice sheets and mountain glaciers shown at 18,000 and 9,000 cal BP[2].* ***b)*** *Left panel, sample ages at Hall's Cave for bulk bone samples (circles) and sediment samples (triangles) based on calibrated ages (ka cal BP). Middle panel, ambient temperature over Greenland based on $\delta^{18}O$ values, dated by annual accumulation layer counting (years before Y2k; Greenland Ice Sheet Project 2 - GISP2[3,4]). Right panel, time period sectioning.*

Subdivision of the Late Quaternary The authors use the Greenland Ice Core data to define the subdivisions of the Late Quaternary. These subdivisions are derived from northern North Atlantic signals present in the ice core and across NW Europe, where they are clearly represented in proxy records in terms of both temperature and hydrology. Exactly how these units are represented in central Texas is MUCH less clear, and I would caution the authors from directly transferring European climate terms to Texas. For example, the authors state that the YD was a "return to cold and dry glacial conditions", whereas data from parts of Texas, New Mexico and Arizona suggests that the Younger Dryas is represented by proxies indicative of cooler but wetter conditions ( ). In addition Meltzer and Holliday (2010; World Prehist (2010) https://doi.org/10.1007/s10963-009-

9032-4) argue that while the Greenland record records abrupt, dramatic hydro-temperature changes at the onset of the YD,
climate changes on the Great Plains and in the Rocky Mountains, were less extreme A similar argument is developed by Holliday et al. (2011; doi:10.1016/j.quaint.2011.03.047).
We agree that the text does not appropriately discuss the caveats of using GISP2 data for a study in Texas. To address this we have added a brief paragraph on the current debate on the severity of the YD in North America:

> *However, current estimates of the amplitude of temperature fluctuations during the late Quaternary relies on data from ice cores in Greenland, which is not readily translated to central North America. For example, the severity of the YD climate change in North America is debatable and the event has been described both as "near glacial conditions"[14] and as a period with mean annual temperatures no more than ~5°C cooler than present[15]. Furthermore, the effect of seasonality during the Younger Dryas is not accounted for with traditional proxies for mean annual temperatures and the apparent cooling during the YD can represent an increased seasonality with cold winters but relatively warm summers[16].*

Furthermore, we have rephrased the description of the Younger Dryas in the first paragraph of the results section which now reads:

> *Younger Dryas (12.6 to 11.5 ka cal BP), a short and abrupt cooling in the Northern Hemisphere*

Specific Points

1) cal BP is usually written in lower case "cal" not CAL
Corrected throughout the manuscript and supplementary information.

2) line 70: "temperatures rapidly returned to glacial conditions in the Northern Hemisphere12." This reference is for Greenland only, and as pointed out above, the YD climate of the southern great plains is not considered to be the same as the LGM. Some recognition needs to be given that the NW North Atlantic record for the LGM-Holocene transition is not applicable in an absolute sense for the Northern Hemisphere as a whole.
Corrected, we have rephrased this to:

> *[…] when temperatures rapidly dropped in the Northern Hemisphere[12]*

…and expanded on the caveats of using the GISP2 data as outlined above.

3) line 92 "approximately 100 bones each" change to "approximately 100 small non-diagnostic bones each"
Corrected.

4) line 105 Omit "For comparative analysis" at start of sentence as appears later in same
Corrected.

5) line 133 Omit "only" end of sentence
Corrected.

6) line 136 "other sources" change to "source other"
Corrected.

7) Line 139 "OTU" Spell out first time
“OTU” was changed to “ASV” (amplicon sequence variant) throughput the manuscript during the review process. ASV is now spelled out on line 139.

8) Line 167 "separated the three time periods" There are 4 time periods.
The plant sampling was focused on the Younger Dryas and only covers the youngest three intervals (B-A, YD, Early Holocene).

9) Line 168 "limited resolution" Do you mean "limited abundance"?
Here we are trying to address the high genetic similarity across quite different species in the RbcL assay. This means that the RbcL assay is not as good at distinguishing species as the trnL assay is. To clarify we have rephrased to:

> *[…] primarily due to the limited taxonomic resolution of the assay*

10) line 235 Delete "has"
Corrected.

11) line 236 The authors soil erosion evidence is certainly NOT identical to that of Cooke et al. (2003) which is based on Hall's Cave Sr isotope ratios and concludes "... a constant minimum rate of 11 cm/k.y.; this continuous phase of erosion ended ca. 5 ka". I think "consistent with" is far more accurate.
Corrected.

12) Line 268 "increased abundance of oak DNA" Abundance is usually difficult to determine for DNA analyses because the PCR amplifies variably. And the correct Supp Fig, is Fig. 9 not Fig, 8. Maybe they mean oak appears more frequently in YD levels than elsewhere?
Yes, thanks for noticing the error. As some of the figures in the supplementary information have been rearranged, the figure is now Supplementary Figure 5.

We agree that abundance is not easily quantified using metabarcoding in a single sample. The observation was based on the abundances of common reads across all samples in Supplementary Figure 5 (previously: Supplementary Figure 9). To be conservative, we have omitted the statement:

> *The Younger Dryas is characterised by the absence of many key warm-climate species such as sagebrush (Anthemideae), bedstraw (Galium), and walnut.*

13) lines 281-282 Ref 34 does not seem relevant to this statement
We agree, the reference was meant as an example of a trophic collapse in North America. The reference has been moved to the following sentence: “Similar collapses have been documented in modern ecosystems[34,35].”

14) lines 297-298 "....evidence supporting dramatic climatic and ecological changes in central Texas after the end of the Last Glacial Maximum" I dont think you can say this. A problem here is that many of your vertebrate taxa go extinct, so that using ecological change as a straight forward proxy for climate is compromised. You might say "evidence supporting dramatic ecological change in central Texas between the LGM and Holocene, some of which is likely due to climate change, but in the same time period humans appear for the first time...."

Thanks for the suggestion. We have rephrased to:

> […] *we find multiple lines of evidence supporting dramatic ecological change in central Texas between the LGM and Holocene.*

15) line 306 "desertification" is defined in way that it cannot be used here. Find another word or words. And it is not clear that colder, drier conditions (which I think is what you mean) "caused" plant and animal diversity to decline. In other parts of Texas and adjacent states "black mats" that formed during YD suggest increased effective moisture (e.g. Harris Park (2017; https://doi.org/10.1016/j.yqres.2015.11.005 and papers by Vance Haynes and colleagues).

Corrected. Sentence was rephrased to:

> *At ~12.6 cal ka, the abrupt change in climate to dry and cool conditions during the Younger Dryas coincides with both plant and animal diversity to decline;*

16) line 310 " decimated by megafaunal extinctions, did not recover" Statement OK, but we need you to define the time that this decimation occurred. This seems to me to be a very key point of this paper. Can you define a time within a specified uncertainty, when the aDNA indicates extinction had occurred?

What can be said, is that the youngest now extinct species in our data is *Haringtonhippus francisci* from 153 cm dated to 12.8 – 12.5 ka. This fits well with the current accepted dates for the extinction horizon between 12.5-13.0 ka. We have added this observation to the text:

> *At ~12.6 ka cal BP, the abrupt change in climate to dry and cool conditions during the Younger Dryas coincides with a decline in both plant and animal diversity. Many animal species, such as burrowing mammals, wetland taxa, and large mammals disappeared permanently from the area during this time, and the last of the now extinct species to disappear from the record (Haringtonhippus francisci) was detected at 153 cm (12.7 ka cal BP), at the onset of the YD.*

17) line 316 "The fact that plant diversity recovered after the Younger Dryas but large vertebrates did not suggests that factors other...." I think this needs to be added to make it clear what you are basing you argument here on...

Corrected.

18) line 325 "...suggest that human hunting of large mammals, likely together with climate change...." I think the fact that you argue that the plants were able to migrate with climate change, and so recover in the Holocene leaves the reader to imagine that large grazing mammals could rather easily migrate with their preferred plant diet sources, hence climate change is not the "obvious" explanation for their extinction. I find the aDNA evidence to make a rather compelling

case for only humans as the extinction source, but recognize that climate cannot be totally ruled out.
This is something that have been discussed passionately among the co-authors. We are aware that this is the conservative interpretation. The current wording is what we could all agree on.

19) line 334 "a" not "an"
Corrected.

20) line 345 "Pollen records from other parts of central Texas suggest that the region was covered in conifer forests during full glacial times" Since you earlier point out that there is NO conifer aDNA and because conifer pollen is exceptionally efficiently dispersed by wind, I expected you to conclude the obvious: "Although pollen records from other parts of central Texas suggest that the region was covered in conifer forests during full glacial times, the lack of conifer aDNA in any of our samples suggests that conifer pollen was aeolian-derived from distant sources". And then modify subsequent text. This seems a very important contribution of this study.
We agree. This has been addressed in a new section on the correlation between plant aDNA and other proxies:

> *Pinus pollen is known for being overrepresented in fossil pollen records due to its high pollen productivity and dispersability[2], while sedaDNA is local in origin[3]. Furthermore, previous results have demonstrated that pollen is essentially devoid of chloroplast DNA[4] explaining the absence of Pinus DNA despite the presence of its pollen. Hence, although pollen records from Hall's Cave and other parts of central Texas suggest that the region was covered in conifer forests during full glacial times, the lack of conifer aDNA in any of our samples suggests that coniferous taxa was not present in the local area but was aeolian-derived from distant sources.*

21) lines 354-55 I would like to see this sentence expanded..what are the climate implications of this important conclusion
Corrected. As outlined above, this section has been moved to the main text and expanded:

> *Despite differences in proxies analysed, our ancient DNA results generally agrees with the trends observed in other paleovegetational records from Hall's Cave (e.g., faunal remains[17], pollen[29], phytoliths[40] and strontium isotopes[19]). The pollen record from Hall's Cave indicate that the vegetation during full glacial conditions was characterised by scattered trees with herbaceous vegetation dominated by C_3 grasses. The pattern of increased woody plant cover during the Bølling-Allerød discussed above is also reflected in the pollen record with a peak in arboreal pollen at 14 ka cal BP followed by a sharp drop at the beginning of the Younger Dryas[29]. In agreement with these results, Joines (2011)[40] found evidence of open woodlands or savannahs during the LGM that transitioned into forests during the Bølling-Allerød. Lastly, although not precisely dated, Boriack bog and Gause Bog[41] in central Texas shows a similar trend of decreasing arboreal pollen during the end of the Pleistocene. As noted by Cordova and Johnson these patterns of a landscape transitioning from an open grassland to a vegetation with an increase in broadleaf trees, suggests an increase in effective moisture during the Bølling-Allerød[42].*

OVERALL

I strongly support publication of this ms in Nat Comms, with the modest suggested changes outlined above. It contains an exceptional mass of aDNA data, maybe unique in the combination of both plant and vertebrate reconstructions across one of the most climatically variable time periods in the Quaternary, and addresses one of the most compelling questions in Quaternary research: what was the impact of continent colonization by modern humans where humans had not previously been present.

Gifford Miller, Univ Colorado

Response to reviewer #2 comments

This is an important paper, with a richly detailed record of community diversity and turnover during a major period of environmental change (the last glacial maximum to early Holocene) and for a region with few paleoecological records. It is also a major demonstration of the potential for bulk-bone and sedimentary ancient DNA techniques for recovering ~species-level genetic signatures of many taxa. My comments below are generally minor to moderate, and are divided into three sections: 1) high-level comments, 2) line-by-line comments (* indicates more substantive comments), and 3) comments on the supplementary info.

MODERATE

1. Diversity and uneven sampling. Alpha richness efforts (such as the ones shown in Figs. 3, 4) are known to be highly sensitive to sampling effort: the more one looks, the more species one will find. The sampling effort per depth interval appears to be fairly constant (e.g. 2 x 50 bulk-bone replicates per sample) but the number of sampled layers differs among the four time periods (17 LGM, 6 B-A, 4 YD, 9 EH). So, for example, could the lower reported diversity during the YD simply be because sampling effort is half as large as for the Early Holocene?

This is unlikely, as each count in the boxplots in figures 3 and 4 represent the total number of species detected in one pool (2x50 bones). Ie. for the YD the boxplot represents 4 observations, while the early Holocene represents 9 observations, but each observation is represented by the same number of bones (100). We have clarified this in the figure, by adding ‘n=’ under each time period.

There are well-established approaches for correcting for sampling effort, via rarefaction analyses. The paper ideally would pursue a rarefaction analysis to correct for sampling effort and at minimum should note the

To address this, we generated species accumulation curves using the R-package vegan for both the plant data and bulk bone data. As depicted in Supplementary Figures 7 and 9 (or see below) the pattern of alpha diversity is consistent with that from Figures 3 and 4.

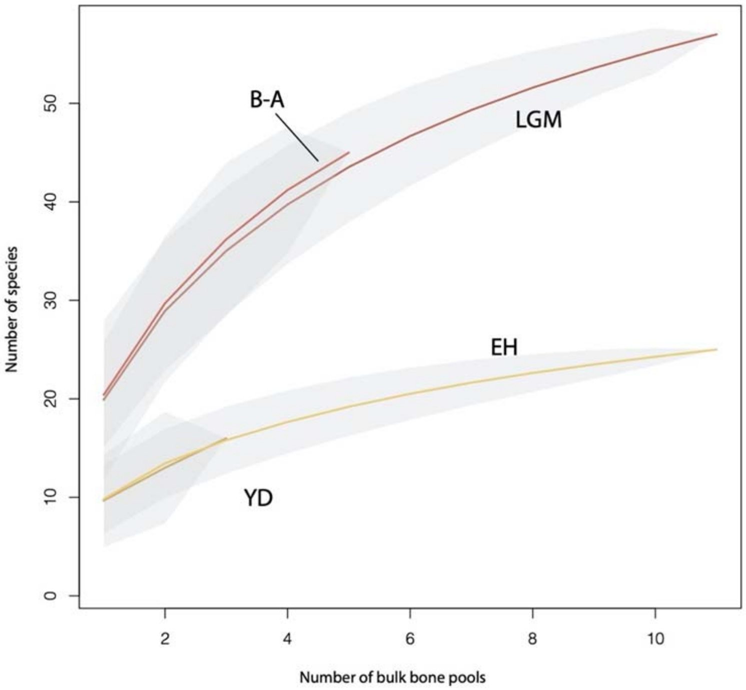

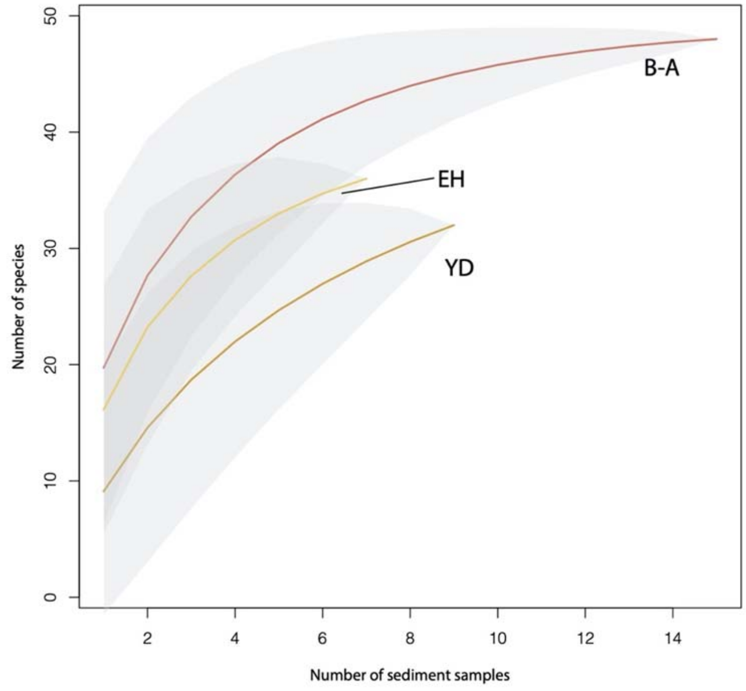

2. I recommend a figure (probably in supplementary information) that repeats the vertebrate diversity figure for different taxonomic subgroups, e.g. for birds, small mammals, megaherbivores,

and reptiles and amphibians. Supp Table 7 suggests that the loss of bird diversity was particularly acute, but this is not apparent in Fig. 3.

We agree. It was not appropriately addressed whether the net loss in alpha diversity could be explained by the disappearance of only one group of species. As suggested, we have generated a supplementary figure that explores this (Supplementary Fig. 8; or see below). As depicted in the figure, the general pattern of diversity loss over the B-A/YD boundary is present in all groups, except for small mammals. We have addressed this in the text:

> *This pattern of species loss is also present when characterising different taxonomic subgroups separately. Alpha diversity loss over the B-A/YD boundary is present in birds, reptiles and frogs, mammalian carnivores and large mammalian herbivores, but is absent in small mammals (Supplementary Fig. 8).*

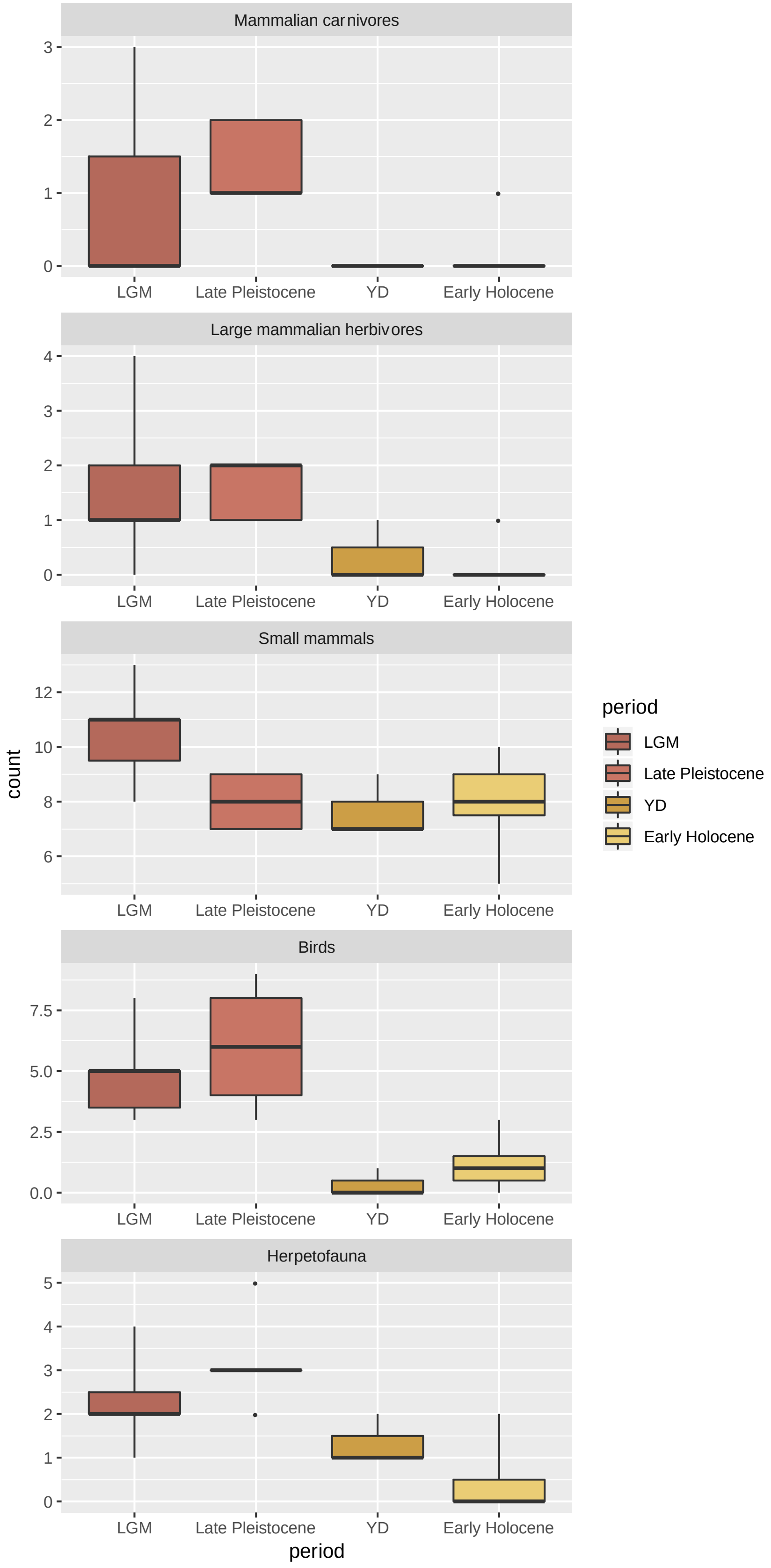
Mammalian carnivores
Large mammalian herbivores
Small mammals
Birds
Herpetofauna
count
period
LGM
Late Pleistocene
YD
Early Holocene
0
1
2
3
4
5
6
8
10
12
0.0
2.5
5.0
7.5

3. The paper tends to be somewhat narrowly focused on its own data and a bit decontextualized from the prior literature on deglacial vegetation and climate dynamics in the central and eastern US. For example, the finding that plant diversity was largely I've suggested below a number of relevant papers.

Corrected. We have added a paragraph where we correlate our data with with other proxies from Hall's Cave and other parts of central Texas:

> *Despite differences in proxies analysed, our ancient DNA results generally agrees with the trends observed in other paleovegetational records from Hall's Cave (e.g., faunal remains[17], pollen[29], phytoliths[40] and strontium isotopes[19]). The pollen record from Hall's Cave indicate that the vegetation during full glacial conditions was characterised by scattered trees with herbaceous vegetation dominated by C_3 grasses. The pattern of increased woody plant cover during the Bølling-Allerød discussed above is also reflected in the pollen record with a peak in arboreal pollen at 14 ka cal BP followed by a sharp drop at the beginning of the Younger Dryas[29]. In agreement with these results, Joines (2011)[40] found evidence of open woodlands or savannahs during the LGM that transitioned into forests during the Bølling-Allerød. Lastly, although not precisely dated, Boriack bog and Gause Bog[41] in central Texas shows a similar trend of decreasing arboreal pollen during the end of the Pleistocene. As noted by Cordova and Johnson these patterns of a landscape transitioning from an open grassland to a vegetation with an increase in broadleaf trees, suggests an increase in effective moisture during the Bølling-Allerød[42].*
>
> *In the pollen data, the YD is characterised by an increase in sagebrush (Artemisia) and a decrease in arboreal pollen. In our data, the decrease in arboreal taxa is reflected by the disappearance of Fraxinus and Juglans during this period. However, the increased abundance of sagebrush contradicts the DNA record, where sagebrush disappears in the YD. Nevertheless, the general trend of increased denudation on the Edwards plateau during this period is evident using both proxies. For example, Cordova and Johnson[29] suggested that the disappearance of Juglans sp. which grows in deep soil, could reflect increased erosion in the area at the onset of the YD, in agreement with our interpretation of soil cover thickness.*

4. The review of vegetation history in the paper would benefit from a closer comparison of the plant sedaDNA data to the pollen and phytolith data from Cordova and Johnson 2019, both in terms of representation of individual taxa and overall ecosystem interpretation. The current comparison is fairly cursory. Suggest merging L346-355 from Methods/Site Description into the main text.

Corrected. We have merged L346-355 with the main text, and added a section comparing the plant aDNA data with the pollen record. Furthermore, we have added a column Supplementary Table 11 indicating if each taxa was detected by pollen (at family level). The new plant aDNA section reads:

> ***Ancient plant DNA compared to other paleoecological records***
>
> *Our sedaDNA plant record generally agrees with previous palynological results from Hall's Cave[29] when comparing the most abundant taxa from both approaches. All of the taxa detected by the most common ASVs in this study (Supplementary Fig. 5) were also detected by pollen, although at different taxonomic levels. Stenaria, for example, which was commonly detected in our study, was identified at family level (Rubiaceae) in the pollen*

data. Similarly, of the ten most abundant taxa identified by pollen, only Pinus and Chenopodiaceae was not identified in the DNA data (Supplementary Table 11). However, the abundances of taxa identified varies widely between the two approaches. Hackberry (Celtis), for example, is identified as the most common read in all sedaDNA samples but is rare in the pollen record. Additionally, the absence of Pinus DNA directly contradicts the pollen record, where Pinus is the most abundant taxa. This discrepancy is likely a result of the different nature of the proxies examined. Pinus pollen is known for being overrepresented in fossil pollen records due to its high pollen productivity and dispersability[37], while sedaDNA is local in origin[30]. Furthermore, previous results have demonstrated that pollen is essentially devoid of chloroplast DNA[22] explaining the absence of Pinus DNA despite the presence of its pollen. Hence, although pollen records from Hall's Cave and other parts of central Texas suggest that the region was covered in conifer forests during full glacial times, the lack of conifer aDNA in any of our samples suggests that coniferous taxa was not present in the local area but was aeolian-derived from distant sources. Nevertheless, as discussed below the overall patterns of vegetational change from the Pleistocene to the Holocene is consistent between the two approaches.

5. Methods: Need to add a section describing the NMDS ordination. What is a taxonomy-independent approach? Why do the results in Fig. 3b differ from the top two rows in Supp Fig. 5a? Why do the results in Fig. 4b differ from the bottom two rows in Supp Fig 5a?

This is because of the different input data used for the figures. This was not clear in the text. While Figures 3 and 4 are based on tables of taxa identified, Supplementary Figure 6 (previously: Supplementary Figure 5) is based on tables of DNA reads identified (ASVs). As expected, we see a similar pattern for the two approaches, but they are not exactly the same. This is because two different DNA reads can be assigned to the same species if they are similar enough. Furthermore, a fraction of the reads (~10%) does not have a match in the database at all. Hence, these reads cannot be assigned to any taxonomic level but they can still contribute to the clustering of the NMDS using the taxonomy independent approach. We have addressed this by adding a new section on statistical analyses in the Methods section:

Statistical analyses

Alpha diversity analyses were carried out on the datasets of identified taxa (Supplementary Tables 13 and 14), excluding contaminants and redundant taxa (e.g. Onychomys sp. was excluded as Onychomys arenicola, and Onychomys leucogaster was detected). NMDS analyses were carried out in R using the Vegan package (https://cran.r-project.org/web/packages/vegan/index.html), based on the same data sets as the alpha diversity estimates. One sample (HCS3) was excluded from the NMDS plot for plants as this sample only contained a single taxon (Celtis). Ordination analyses using a taxonomy independent approach (Supplementary Fig. 6) were carried out as described above using the ASV tables as input.

6. Paper would be strengthened by a summary figure (perhaps in Supplementary Info) that summarized the interpreted environmental history at Halls Cave. i.e. with an x-axis for time and then different horizontal plots indicating e.g. trends in temperature, soil depth, ecosystem type, and diversity.

Thanks for the suggestion. We have added an overview figure (Supplementary Fig. 12; or see below) with a general interpretation of the past climate and ecology around the cave.

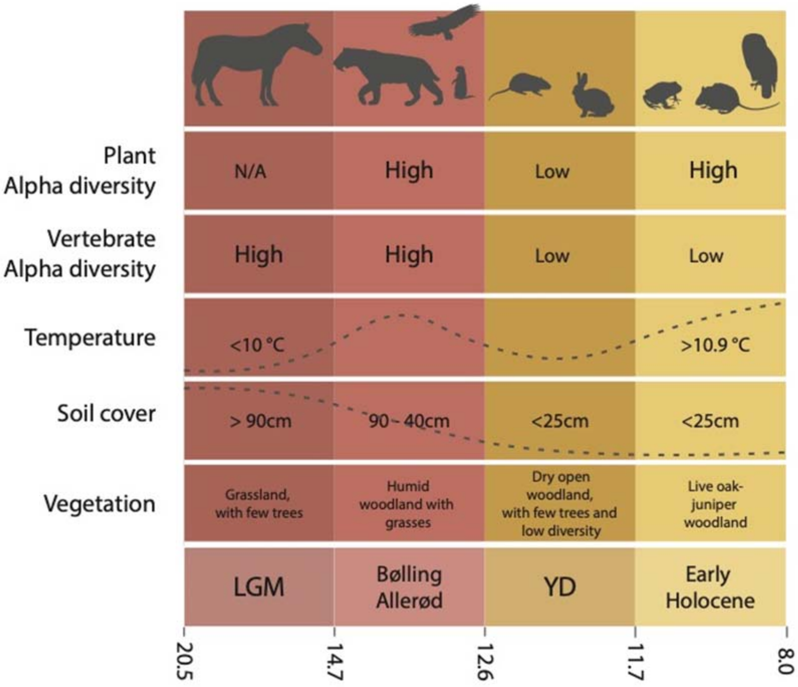

LINE-BY-LINE COMMENTS (* indicates more substantive points)

L40-42: This is an oversell – there are *many* high-resolution fossil pollen records spanning the Younger Dryas, in eastern North America, Europe, and elsewhere, that are well able to characterize the pace and scale of biotic turnover. See e.g. Gonzales and Grimm et al. 2009 QR; Williams et al. 2002 Geology

Corrected. We have rephrased the sentence to:

> *However, the pace and scale of biotic turnover in response to both the Younger Dryas cold period and subsequent Holocene rapid warming have been challenging to assess because of the scarcity of well dated fossil and pollen records that covers this period.*

L64: The dates of Widga et al. 2017 Boreas suggest a slightly later extinction date for mastodons, ca. 12.7 to 12.5 ka.

Thanks for the reference. We have updated the text to reflect the dates from Widga et al:

> *[…] by approximately 12.5-13.0 ka cal BP*[8,9].

L72-74: Delete this sentence on ‘difficult to describe’ climatic events YD & BA or extend to review what is known. There is a massive paleoclimatic literature on both events, with respect to both proxy data and paleoclimatic models; these are very well-studied & understood climatic events. See e.g. Peter Clark et al. 2012 PNAS; Shakun & Carlson 2010 QSR; Shakun et al. 2012 Nature.

We agree. The sentence was deleted and a paragraph elaborating on the current debate on the YD severity in North America was added:

However, current estimates of the amplitude of temperature fluctuations during the late Quaternary relies on data from ice cores in Greenland, which is not readily translated to central North America. For example, the severity of the YD climate change in North America is debated and the event has been described both as "near glacial conditions"[1] and as a period with mean annual temperatures no more than ~5°C cooler than present[5]. Furthermore the effect of seasonality during the Younger Dryas is not accounted for with traditional proxies for mean annual temperatures and the apparent cooling during the YD can be represent an increased seasonality with cold winters but relatively warm summers[6].

L78: upper-case 'central' or lower-case 'North' for consistency
Corrected.

L82: Insert 'cave' before 'sites' – many lakes have very good YD records. For example, the ca. 20cm interval representing the YD at Hall's Cave might be 1-2 m of sediment in many eastern US kettle lakes.
Corrected.

L86 delete hyphen from 'turn-over'
Corrected.

L104: insert 'while' before 'acknowledging'
Corrected.

L107-108: insert 'in the Northern Hemisphere' at the end of item 3; YD cooling is mainly a NH phenomenon
Corrected.

L115: which targeted time interval? Four are mentioned in the previous section.
Here, we meant the entire interval analysed. We have reworded the sentence to:

[…] high level of vertebrate animal diversity at Hall's Cave using bulk bone metabarcoding.

L115: insert 'species' after 'vertebrates'
Corrected.

L133: 'only' shows up twice here
Corrected.

L137: insert comma after 'hair'
Corrected.

L141: delete comma after 'Celtis'
Corrected.

L143: it->they
Corrected.

L145: significantly -> substantively ('significantly' implies a statistical test)
Corrected.

L148: form->from
Corrected.

*L148-151: Further sharpen by noting that Pinus is particularly known for being overrepresented in fossil pollen records, due to its high pollen productivity and dispersability. See e.g. Dawson et al. 2016 QSR. So this particular data-data mismatch is interesting but not particularly surprising.
Corrected. This was addressed in the new section 'Ancient plant DNA compared to other paleoecological records':

> *This discrepancy is likely a result of the different nature of the proxies examined. Pinus pollen is known for being overrepresented in fossil pollen records due to its high pollen productivity and dispersability[2], while sedaDNA is local in origin[3].*

L148-151: Also worth noting that in Cordova & Johnson 2019, Celtis is a rare type, whereas it is the most common type in the sedaDNA record.
Corrected. This was addressed in the new section 'Ancient plant DNA compared to other paleoecological records':

> *Hackberry (Celtis) for example which is identified as the most common read in all sedaDNA samples is a rare type in the pollen record.*

Figure 1: The Great Lakes and other pro-glacial lakes are incorrectly color-coded, so that they appear to be part of the 9ka Laurentide ice sheet.
We thank the reviewer for noticing this. It has been corrected.

L154: CAL -> cal. Also fix in Figure 1 legend, axis titles, and elsewhere.
Corrected.

L155: Clarify that right panel shows air temperature *over Greenland* and is based on d18Oice.
Corrected.

L163: was consistent ->persisted
Corrected.

L164: what kind of 'public reference databases'? Genomic?
Yes, genomic. We have clarified this in the manuscript. The text now reads:

> […] *which controls for uneven representation of species in the genomic reference database used (see Methods; Supplementary Fig. 6).*

L165: similar to what? Comparison is unclear.
We have rephrased the text to clarify:

> *For plants, NMDS based on taxa revealed a clustering by time period ($P < 0.001$, 999 permutations), similar to that observed for vertebrates.*

L168: what kind of resolution? Taxonomic?
Yes. Corrected.

*L170 & Figure 3: If the extinct taxa were not included in the analysis, would there still be a net decline in diversity?
We tested this in the figure below. Compared to the full data set, the number of extinct taxa is very low, and removing them does not change the plot significantly:

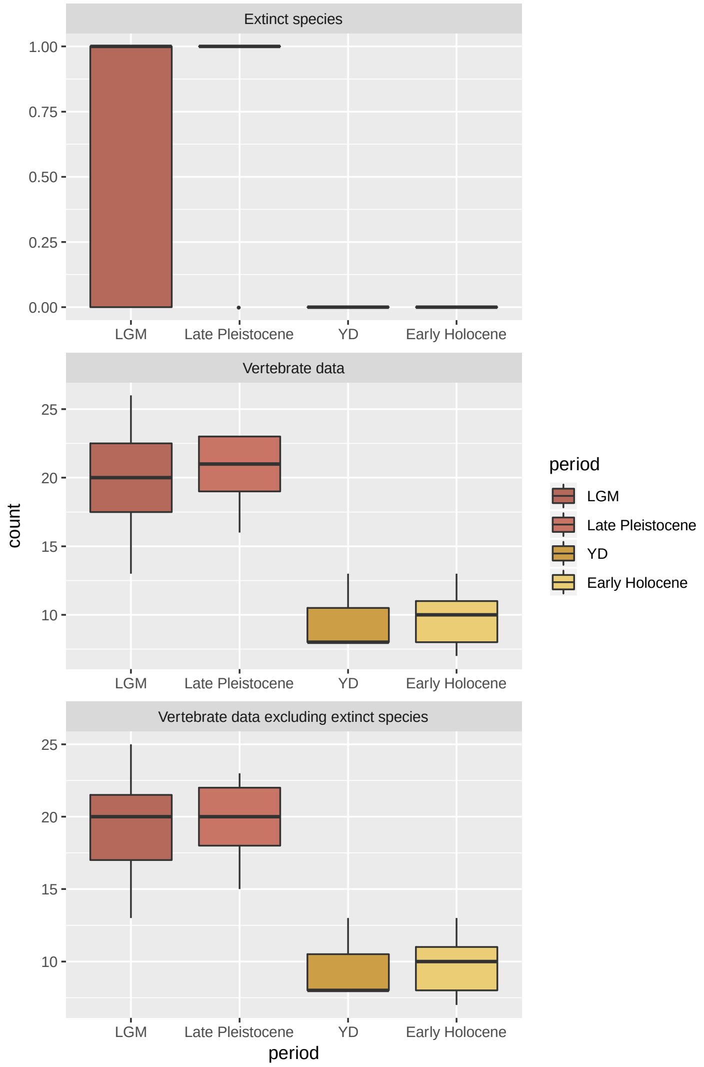

Also, do the small and large mammals show different diversity trends? Suggest creating a few additional variants on panel Fig. 3c, either in main text or Supp Info.
We agree, it is highly relevant to look at these patterns more closely. We have addressed this above, as this was also requested by reviewer one.

L169-172: All these sentences are written in first person, which adds words and shifts the subject/action emphasis away from the actual findings to the scientists. For example, the second sentence could easily be rewritten as 'For vertebrates, diversity significantly declined from the

Pleistocene…' This slight rewrite is both more word-sparing and strengthens the subject/verb pair from 'we found' to 'diversity declined'…
Corrected. The text now reads:

> *The alpha diversity of plants and animals within each layer displayed different patterns of species loss and recovery over time (Fig. 3c and 4c). For vertebrates, diversity significantly declined from the LGM and Bølling-Allerød time periods (mean=19.6, SD=4.0) to the YD and Early Holocene (mean=9.5, SD=1.8; Supplementary Fig. 7). This pattern of species loss is also present when characterising different taxonomic subgroups separately. Alpha diversity loss over the B-A/YD boundary is present in birds, reptiles and frogs, mammalian carnivores and large mammalian herbivores, but is absent in small mammals (Supplementary Fig. 8). Similarly, for plant species, diversity declined from the Bølling-Allerød (mean=15.0, SD=3.74) to the Younger Dryas (mean=7.5, SD=3.1).*

L175: 'the climate warmed' -> 'temperatures rose'
Corrected.

Figure 2: What a beautiful data visualization!
Thanks!

L187: 'the temperature' -> 'Greenland temperature'
Corrected.

L191: delete 'cold climate'
Corrected.

*L197, L454 and elsewhere: Avoid use of 'tolerance.' Use of geographic data can show the current realized niche limit of a species, but cannot directly speak to the true fundamental climatic tolerances of a species. So e.g. L197 replace 'biological tolerances' with 'current realized climatic niches' and L454 replace 'Climate tolerance limits' with 'Climatic realized niches' or 'Climatic realized niche limits'.
We agree. It has been corrected throughout the manuscript and the SI.

L198: 'refine inference of the climate' – awk
Rephrased to:

> *[…] we are able to infer past temperatures around Hall's Cave.*

L204: a notable -> substantial
Corrected.

Fig. 3/L214-219: Clarify – is this figure showing data from both Sequences A & B?
Yes. We have clarified this in the figure legend:

> ***a)*** *Detection of select indicator species through time. Includes data from sequences A and B sorted by age.*

Fig. 4/L243-246: Remind reader that it's the trnL data being analyzed here.

Corrected. The first lines of the legend now reads:

> ***Figure 4. Plant diversity through time.*** *Record is based on sedaDNA data of two short chloroplast assays (trnL-gh and rbcL).*

L248-265: Need to add citation here to Cordova & Johnson 2019 and integrate this interpretation with the available pollen and phytolith record from this site.
Corrected. As described above we have added the section 'Ancient plant DNA compared to other paleoecological records'.

L249: Clarify: is this detection based on bulk bone aDNA or traditional bone identification?
Corrected. The sentence now reads:

> *Turnover in fauna detected in our bulk bone record […]*

L253: woody plants -> woody plant cover [or density]
Changed to *woody plant cover*

L269: delete extra 'species'
Corrected.

L273: lower case for 'Flowers'
Corrected.

L275: insert 'in' after 'increase'
Corrected.

L276: larger than what? Unclear comparison.
Changed to *For large mammals (>30kg)* […]

L286: Suggest also citing here Gill et al. 2009 Science for another example of Pleistocene megaherbivore-vegetation interactions in eastern North America
Thanks for the very relevant reference. It has been cited at the end of the paragraph.

L288: For the non-analog taxa, suggest citing Graham et al. 1996 Science and Williams and Jackson 2007 Frontiers in Ecology and Environment. Russ Graham has many papers about the disharmonious (no-analogue) mammal assemblages of eastern North America
Thanks for the references, they have been added to the text.

L313: Delete 'forever' – that's a very long time…
Good point! Forever was deleted.

L314-318: Might note here that, in contrast with vertebrates, there is only one documented extinction of a plant species in eastern North America during the last deglaciation. See Jackson & Weng 1999 PNAS.
Thanks for the suggestion. We have rephrased the text to include this point:

While 34 genera of large mammals went extinct during the late quaternary[2], there is only one documented example of extinction in plants. Similarly, our data shows that plant diversity recovered after the Younger Dryas, while the diversity in large mammals did not. This suggests that factors other than climate, including the appearance of humans in the region, may have contributed to the permanent local loss of large mammal diversity.

L335: Could broaden the time scale range by changing to 'centuries to tens of millennia'
Good idea, the text now reads:

Such multidisciplinary approaches are increasingly employed to provide suites of complementary proxies that better quantify our reconstructions of ancient climates, past biodiversity, extinctions, and biotic shifts over centuries to millennia.

L344: grassy->grassland
Corrected.

L353: the combination of 'e.g.' and 'i.e.' here is hard to follow. Suggest rewriting as 'direct vegetation indicators such as phytoliths vs. indirect indicators such as faunal remains'
Corrected.

*L344-355: This whole section belongs in the main paper, so that the sedaDNA intepretations of vegetation history can be integrated with other lines of evidence. This section also omits mention/discussion of Cordova & Johnson 2019 (ref 24); a major omission.
We agree. As described above, this have been addressed in the section 'Ancient plant DNA compared to other paleoecological records'.

*L355: I'd be more cautious about the apparent interpretation here of LGM grasslands. The floral sedaDNA evidence doesn't extend to the LGM (Fig. 4), while the mammalian species occurrences (Figs. 2-3) could be interpreted as grassland to open woodland. Also the 'by end of the Pleistocene' implies a unidirectional change from Pleistocene to Holocene vegetation, while authors' data suggest a more complex pattern, with a YD reversal along the way. Suggest extending this by a sentence or two to be more precise about the inferred vegetation changes.
We agree. This paragraph have been rephrased and moved to the main text. Furthermore, we have elaborated on the plant data interpretations, and compared these to existing data as described above.

L362: Should the units here be yr/cm, not just 1/cm?
The unit for k_0 is events per cm. The interpolation rate ($2cm^{-1}$) specifies how often the model should output data (i.e. twice per centimeter). However, as we have updated the age-depth model as per reviewer one's suggestions these variables are not relevant anymore.

*L375: In Sampling section, describe the depth datums – see queries in Supp Info.
Until formal UTM coordinates were established at Hall's Cave in August 2016, (see figure below) excavation data were referenced to Rickard Toomey's definition of 0 cm BD. Horizontal (X-Y) positions of excavation pits were relative to a nail in cave wall limestone, Point Number 850 (figure below). In August 2016, funding through Dr. Timothy Rowe, Department of Geological

Sciences, University of Texas- Austin, enabled the professional surveying company, Urban Civil LLC, New Braunfels, Texas to emplace three bronze benchmarks outside the cave and nine internal control points (ICP 11-19) within the cave. The global coordinates of Toomey's wall nail were also established to enable previous excavations to be located with the UTM system. All paleontological, geological, and archaeological excavations starting in August 2016 and afterwards have been done using Total Station readings from ICP markers within Hall's Cave.

The elevation of the Toomey 0-cm Datum (cm BD_T) is 665.890 m ASL. Coordinates for the secondary, nail datum are N. 3333866.182 m, E. 448166.974 m, 667.63 m ASL. Presently, a minimum of 388 cm of stratified sediments are known, although identification of absolute bedrock limestone is uncertain. The most diagnostic stratigraphic marker is the base of the Younger Dryas (YD), which is approximately horizontal across the North-South, A-B plane designated as the cave's principal geologic section (Figs. 1, 3). The YD base's elevation is 664.380 m ASL on the A-B plane, 151 cm below the Toomey datum.

We compared our excavation notes with the UTM Benchmark data established by Urban Civil, LLC, August 2016. From this comparison, we realized that our sample depths were recorded according to Toomey's datum, and not the modern surface. We have corrected this error throughout the manuscript. With the new age-depth model implemented as per reviewers 1's suggestion, this does not change the data substantially.

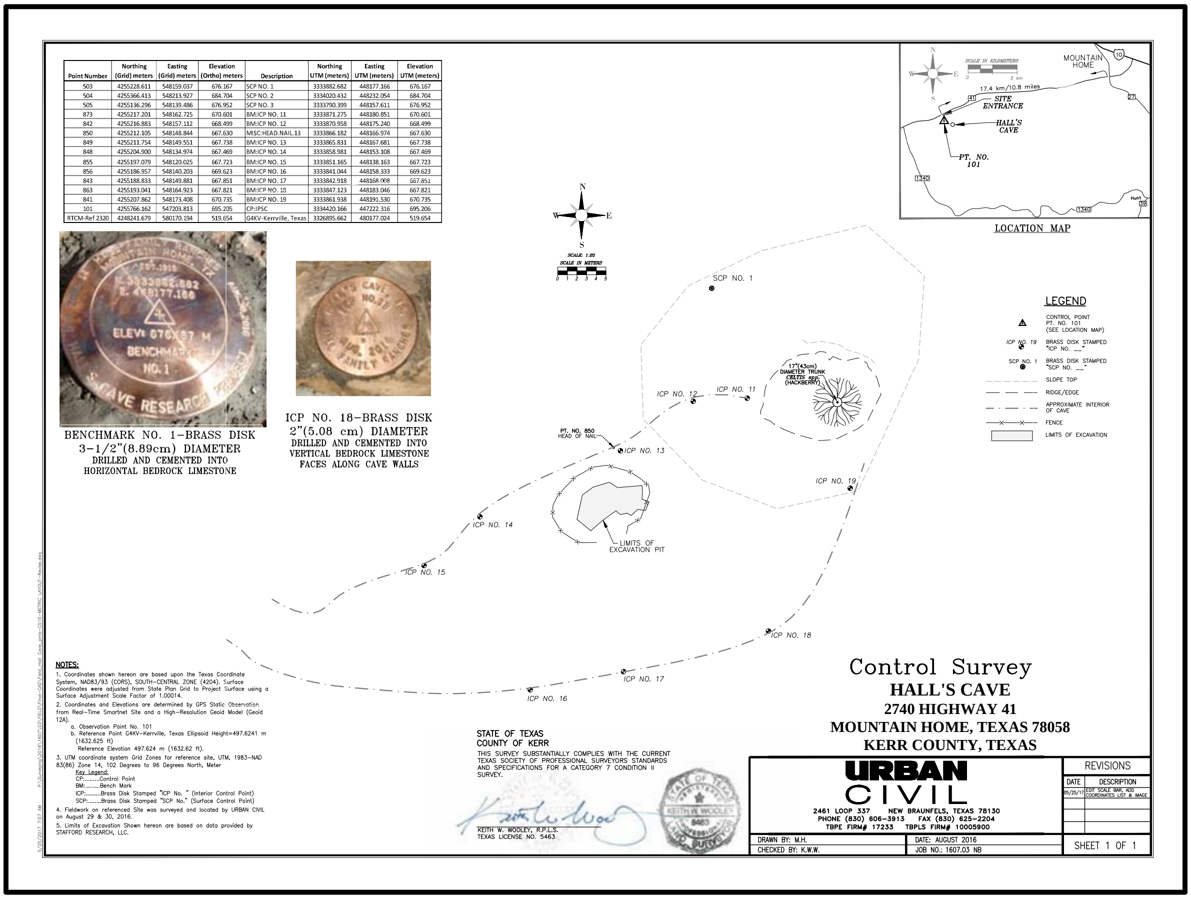

We have clarified the depth datum used in the sampling section:

Sample depths were recorded as cm below the zero datum established by Toomey (cm BD_T). To better enable future researchers to correlate new and old data collected at the cave, we have included absolute elevation for each sample in Supplementary Table 1 and 4, from UTM Benchmark data established by Urban Civil, LLC, in August 2016.

SUPPLEMENTARY INFO

Figure S1: What defines the zero depth datum? Specify. Also specify the collection unit (pit 1d/E?).

See above for the definition of the zero depth datum.

The September 2016 aDNA excavations were a 1 m X 0.5 m unit located on the eastern edge of 1990-2015 excavations. To clearly identify the location of the eastern face of excavation pit 1d/E, we have included a schematic with the layout of the existing pit and highlighted the face excavated for this study (Supplementary Figure 14 or see below).

Layout of Composite Pit I

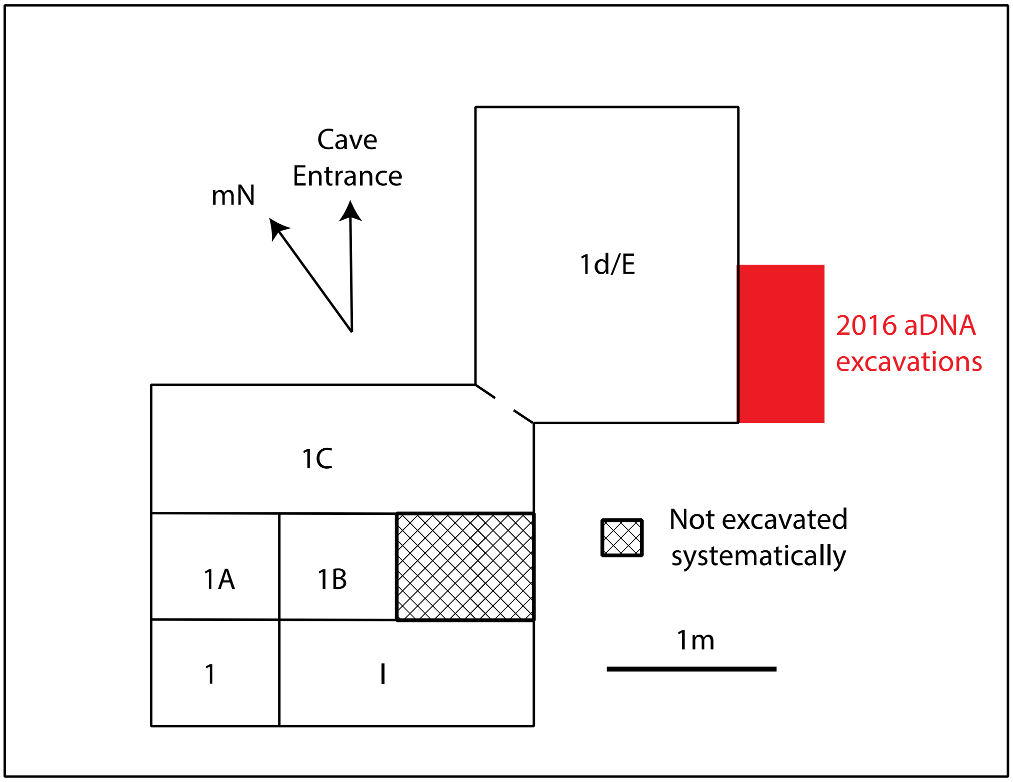

Figure S3: Please add the common-name equivalents for all taxa listed in legend.

Corrected.

Figure S4: Remind reader what the crosses after taxon names indicate.

Corrected. The last line of the legend reads:

Crosses (†) indicate extinct genera.

Figure S5: Remind reader what the panel titles mean. Also clarify here that this is a 'taxonomy-independent approach' and explain what this means. This is alluded to in main text (163-165) and needs more detail here.
We have elaborated on the approach in the legend:

> ***Supplementary Figure 6. Taxonomy-independent ordination analysis based on ASV diversity.*** *The two upper panels represent bulk bone data analysed with mitochondrial assays targeting vertebrates (Mam16S and 12Sv5; subsampled to 7247 reads per sample), whereas the two lower panels represent sediment samples analysed with chloroplast assays targeting plants (rbcL and trnL; subsampled to 5374 reads per sample). As opposed to figures 3b and 4b in the main text which are based on the taxonomic record inferred from the DNA data, these ordination analyses are based on ASVs (amplicon sequence variants). Hence, this approach compares DNA sequences across samples without assigning these to taxa first. Accordingly, unknown sequences without matches in the database can be taken into account.*

Figure S10: What is the Toomey datum, where is it, and how is it defined or recognized?
See above.

Recommend reversing x-axis. I'm a strong proponent of time running forward from left to right; this follows the western convention of reading words from left to right. Reversing Fig 10 would also align it with the convention followed in Figs. 3-4.
Corrected.

Table S1: What are the bottom seven units and why do they lack metadata?
They are the six bulk bone samples made from large fragmentary fossils excavated by Toomey in 1993. These were analysed as a pilot study before systematic sampling took place. There is a question-mark in column one as the number of bones analysed in these samples were recorded as 'approximately 100 bone fragments'. We have updated the entry in the table to '~100'. The absence of data in column two, three and four reflects that the samples were excavated by Toomey who used a different datum. We have not modelled an age from sample 1C_240_245 which was excavated from pit 1c and not composite pit 1d/E. However, as noted in the methods section, this sample was excavated from 240-245 cm below Toomey's datum (cm BD_T), approximately 100 cm below the onset of the Younger Dryas. Thus, we can confidently assign this sample to *'Last Glacial Maximum'*. (Please note that the Toomey samples were excluded from the diversity comparisons in figure 3b and 3c.)

We have clarified this in the legend:

> *The last six samples represent large fragmentary fossils excavated by Toomey in 1993. As these samples were not excavated as part of this study there is no information on excavation interval or depth measured from the datum line of this study. Furthermore, the exact number of bones analysed for these samples is not known as they were recorded as 'approximately 100 bone fragments'.*

Table S6: What is meant by 'upgrade' and 'downgrade'? And, what does the animaldiversity.org URL support? The dietary status of each taxon?
We have elaborated on this in the legend. It now reads:

> ***Supplementary Table 7. Mammal species identified.*** *Only lowest taxonomic nodes detected are shown. Some taxonomic nodes were either upgraded (∧; family -> species) or downgraded (∨; species -> family) based on database coverage and species distribution. EH: Early Holocene, YD: Younger Dryas, BA: Bølling-Allerød, LGM: Last Glacial Maximum. (x) signifies that the genus was identified before, but that the taxa could not be resolved to species level by morphology. * Common laboratory contaminants. C: carnivore (of terrestrial vertebrates), I: Insectivore, H: Herbivore. Dietary preference was sourced from: https://animaldiversity.org.*

Table S7: birds->bird
Corrected.

Response to reviewer #3 comments

Reviewer #3 (Remarks to the Author):

The authors present a nice data set from a very special, long, continuous deposit.

Overall a larger diversity (number of species) is reported for the older (Pleistocene) sample, however that sample also contained a larger number of subsamples from a longer time period. Although I am inclined to believe the pattern reported, I am not convinced that the presented analyses show that. It could be more appropriate to use a diversity index, or subsampling to make the different samples (time periods) more comparable.
We agree. To address this, we have included species accumulation curves for both plants and animals. Using these approaches, we find similar patterns as those represented in Figures 3 and 4. Please see our response to Reviewer 2's "moderate" comments 1 and 2 for a detailed answer.

Also regarding the sampling (pg 19, sup) "the disappearance of most carnivores and large mammals at the beginning of the Younger Dryas period indicates that accumulation by large carnivores diminished over time" how do you think the change in the way bones accumulated in the cave through time impacts the pattern observed? Could a change in usage of the cave by large carnivores explain the disappearance of mammals too large for an owl to catch? (without requiring a change in the local population? ie. Fig 3, lines 238-241; lines 276-282; lines 317-318- this is really hard to differentiate from change in mode of bone deposition)
We agree that this was not properly addressed in the text. We have added a paragraph elaborating on the matter:

> *Furthermore, as carnivores represents the top of the food chain, their disappearance is unlikely to be a result of a change in deposition but must reflect a change in the surrounding ecosystem. The disappearance of large herbivores at the onset of the Younger Dryas, on the other hand, could be linked to the disappearance of carnivores. Still, the continued presence of raptors in the cave suggests that the loss of diversity in frogs and reptiles in the Holocene reflects a loss of these species in the area surrounding the cave. Compared to the faunal assemblage, the plant data are less affected by taphonomic processes. As noted by Toomey, hackberry seeds might have been washed into the cave from the surrounding land surface, but other dispersal routes, such as wind or transportation on birds, insects and other animals would also have contributed to the plant assemblage[25]. However, as the depositional processes for the plant and animal assemblages are very different, the two assemblages serve as important validations of each other.*
>
> *In combination, data from pollen, plytholiths, sedimentary DNA, bulk bone DNA and morphological bone identifications details how the landscape changed around Hall's Cave from the Pleistocene to the Holocene. While certain species groups could be affected by a change in depositional processes, it is very unlikely that all species are.*

Lines 148-151 re Pinus- it sounds as if there is a pollen record including Pinus in the cave- is this correct? If so, one could expect to find DNA from Pinus (from the pollen) regardless of whether it grew adjacent to the cave or the pollen was dispersed potentially long distances. Please clarify.

This discrepancy is likely a result of the absence of chloroplast DNA in pollen (see Willerslev et al. 2003, Science). We have clarified this in the text:

> *Furthermore, previous results have demonstrated that pollen is essentially devoid of chloroplast DNA*[4] *explaining the absence of Pinus DNA despite the presence of its pollen.*

The habitat modeling is a nice complement to the story.

Thanks!

Lines 416-419- put final concentrations of reagents (final molarity, number of units, etc) in total volume.

Corrected.

REVIEWERS' COMMENTS:

Reviewer #1 (Remarks to the Author):

I have read through the authors responses to my comments on 229399 (Rapid range shifts and megafaunal extinctions driven by late Pleistocene climate change) and am satisfied with all responses with only one minor disagreement as follows: My point 18) on the sentence in line 325 "...suggest that human hunting of large mammals, likely together with climate change...." in which I felt that a major point of the ms was to demonstrate that plant ecosystems were able to migrate efficiently and without extinction to the imposed rapid climate change of the Younger Dryas, whereas large grazing animals dependent on those ecosystems failed to do so, and hence became extinct. I think most objective readers would conclude these data provide strong evidence that climate was not the primary cause of megafaunal extinction, although I recognize that climate cannot be totally ruled out as an ancillary pressure. The authors admitted that this was a hotly debated point amongst themselves and that "The current wording is what we could all agree on".

I am comfortable leaving the text as written, in particular because the authors have included the addition of the following sentence in the abstract "Our findings demonstrate that while climate change affected the local ecosystem in Texas over the Pleistocene/Holocene boundary, it cannot alone explain the disappearance of the megafauna at the end of the Pleistocene."
As long as that sentence appears in the abstract I am confortable with the ms text as revised

Gifford Miller University of Colorado Boulder

Reviewer #2 (Remarks to the Author):

It was a pleasure to read this revised paper and I'm also pleased to report that all of my major comments on the original manuscript have been satisfactorily addressed during revision. It's an important paper and I very much look forward to seeing it in print. Cheers, Jack Williams, UW-Madison

A few minor comments:
L64: reverse to 13.0-12.5, to follow convention of older-younger
L75: be more geographically precise than 'North America' – North America is big and the YD likely manifested differently in different areas.
L73-79: Also, my group has two papers recently out that are relevant to this section. They present deglacial temperature reconstructions for north-central North America (Ohio), albeit with fairly high uncertainty (Watson et al., 2018, Fastovich et al., 2020).
L83: decapitalize 'North'
L95: 'Early Holocene' is not a formally defined time period (yet) so change to 'early Holocene'
L128-134: Some switching of verb tense in this paragraph; check here and elsewhere for consistency of tense.
L151: were resident -> resided
L210-211: suggest rephrasing from 'the Greenland temperature fluctuations that occurred over the Pleistocene-Holocene boundary' to 'Greenland temperature fluctuations during the Pleistocene-Holocene transition'

L322: agrees->agree
L324: hyphenate 'full glacial'
REFERENCES
Fastovich, D., Russell, J.M., Jackson, S.T., Williams, J.W., 2020. Deglacial temperature controls on no-analog community establishment in the Great Lakes Region. Quaternary Science Reviews 234, 106245.

Watson, B.I., Williams, J.W., Russell, J.W., Jackson, S.T., Shane, L.C.K., Lowell, T.V., 2018. Temperature variations in the southern Great Lakes during the last deglaciation: Comparison between pollen and GDGT proxies. Quaternary Science Reviews 182, 78-92.

Reviewer #3 (Remarks to the Author):

Abstract (last sentence), Line 383-386: this is overstated. The large mammals couldn't come back because they had gone globally extinct, not just locally extinct. The habitat requirements of small mammals are simply less than large mammals. It is possible that they went extinct with the "help" of humans, but it is not necessarily so.

REVIEWERS' COMMENTS

Reviewer #1 (Remarks to the Author):

I have read through the authors responses to my comments on 229399 (Rapid range shifts and megafaunal extinctions driven by late Pleistocene climate change) and am satisfied with all responses with only one minor disagreement as follows: My point 18) on the sentence in line 325 "...suggest that human hunting of large mammals, likely together with climate change...." in which I felt that a major point of the ms was to demonstrate that plant ecosystems were able to migrate efficiently and without extinction to the imposed rapid climate change of the Younger Dryas, whereas large grazing animals dependent on those ecosystems failed to do so, and hence became extinct. I think most objective readers would conclude these data provide strong evidence that climate was not the primary cause of megafaunal extinction, although I recognize that climate cannot be totally ruled out as an ancillary pressure. The authors admitted that this was a hotly debated point amongst themselves and that
"The current wording is what we could all agree on".

I am comfortable leaving the text as written, in particular because the authors have included the addition of the following sentence in the abstract "Our findings demonstrate that while climate change affected the local ecosystem in Texas over the Pleistocene/Holocene boundary, it cannot alone explain the disappearance of the megafauna at the end of the Pleistocene."
As long as that sentence appears in the abstract I am confortable with the ms text as revised

Gifford Miller University of Colorado Boulder

We hope that the edits suggested by the editor is a satisfactory compromise.

Reviewer #2 (Remarks to the Author):

It was a pleasure to read this revised paper and I'm also pleased to report that all of my major comments on the original manuscript have been satisfactorily addressed during revision. It's an important paper and I very much look forward to seeing it in print. Cheers, Jack Williams, UW-Madison

A few minor comments:
L64: reverse to 13.0-12.5, to follow convention of older-younger
Corrected.

L75: be more geographically precise than 'North America' – North America is big and the YD likely manifested differently in different areas.
Corrected to *on the Great Plains*

L73-79: Also, my group has two papers recently out that are relevant to this section. They present deglacial temperature reconstructions for north-central North America (Ohio), albeit with fairly high uncertainty (Watson et al., 2018, Fastovich et al., 2020).
Thanks for the references. We have cited Watson et al. in the results section as an example of pollen records that show a delayed response to the YD-cooling over Greenland.

L83: decapitalize 'North'

Corrected.

L95: ‘Early Holocene’ is not a formally defined time period (yet) so change to ‘early Holocene’
Corrected.

L128-134: Some switching of verb tense in this paragraph; check here and elsewhere for consistency of tense.
Corrected.

L151: were resident -> resided
Corrected.

L210-211: suggest rephrasing from ‘the Greenland temperature fluctuations that occurred over the Pleistocene-Holocene boundary’ to ‘Greenland temperature fluctuations during the Pleistocene-Holocene transition’
Corrected - thanks for the suggestion.

L322: agrees->agree
Corrected.

L324: hyphenate ‘full glacial’
Corrected.

REFERENCES

Fastovich, D., Russell, J.M., Jackson, S.T., Williams, J.W., 2020. Deglacial temperature controls on no-analog community establishment in the Great Lakes Region. Quaternary Science Reviews 234, 106245.
Watson, B.I., Williams, J.W., Russell, J.W., Jackson, S.T., Shane, L.C.K., Lowell, T.V., 2018. Temperature variations in the southern Great Lakes during the last deglaciation: Comparison between pollen and GDGT proxies. Quaternary Science Reviews 182, 78-92.

Reviewer #3 (Remarks to the Author):

Abstract (last sentence), Line 383-386: this is overstated. The large mammals couldn’t come back because they had gone globally extinct, not just locally extinct. The habitat requirements of small mammals are simply less than large mammals. It is possible that they went extinct with the “help” of humans, but it is not necessarily so.

We hope that the edits suggested by the editor is a satisfactory compromise.
